# The E3 ubiquitin ligase HECTD3 controls immune colonic inflammation by restricting MyD88 activation and signaling

**DOI:** 10.1172/jci.insight.185437

**Published:** 2026-09-24

**Authors:** Shamima Islam, Shahnewaj Mannan, Ashley Zuniga, Upasana Parthasarathy, Valeriu B. Cismasiu, Leonardo Silvane, Sayan Chakraborty, Divya Priyanka Talada, Raghwendra Pratap Singh, Tomas Zelenka, Amreen Naveen, Sephra Chyanne Vickers, Michael G.M. Grant, Jonathan J. Cho, Theodore Drashansky, Alexander J. Kwiatkowski, Xintong Liu, Ross Tomaino, Olga A. Guryanova, Mariola J. Ferraro, Sang Yong Kim, Christian Jobin, Benjamin G. Keselowsky, Hongmin Li, Paulo C. Rodriguez, Martina Molgora, Amer A. Beg, Timothy I. Shaw, Zheng Ruan, Lixin Wan, Dorina Avram

**Affiliations:** 1 Department of Immunology, H. Lee Moffitt Cancer Center and Research Institute, Tampa, Florida, USA.; 2 Department of Anatomy and Cell Biology, College of Medicine, University of Florida, Gainesville, Florida, USA.; 3 Department of Molecular Oncology, H. Lee Moffitt Cancer Center and Research Institute, Tampa, Florida, USA.; 4 J. Crayton Pruitt Family Department of Biomedical Engineering, University of Florida, Gainesville, Florida, USA.; 5 Department of Pharmacology and Toxicology, R. Ken Coit College of Pharmacy, Department of Chemistry and Biochemistry, College of Science & College of Medicine, The BIO5 Institute, The University of Arizona, Tucson, Arizona, USA.; 6 Taplin Mass Spectrometry Facility, Harvard Medical School, Boston, Massachusetts, USA.; 7 Department of Pharmacology and Therapeutics and; 8 Department of Microbiology and Cell Science, IFAS, University of Florida, Gainesville, Florida, USA.; 9 Department of Pathology, NYU School of Medicine, New York, New York, USA.; 10 Division of Gastroenterology, Hepatology, and Nutrition, Department of Medicine, University of Florida, Gainesville, Florida, USA.; 11 Department of Biostatistics and Bioinformatics, H. Lee Moffitt Cancer Center and Research Institute, Tampa, Florida, USA.; 12 Department of Biochemistry & Molecular Biology, Thomas Jefferson University, Philadelphia, Pennsylvania, USA.

**Keywords:** Immunology, Inflammation, Inflammatory bowel disease, Ubiquitin-proteosome system

## Abstract

Ubiquitination is an important posttranslational modification associated with essential cellular processes. Here we show that deletion of E3 ubiquitin ligase HECTD3 — germline or in hematopoietic compartment, including in Cd11c^+^ cells — causes severe disease and increased production of proinflammatory cytokines in a murine model of ulcerative colitis (UC). HECTD3 mRNA levels were found reduced in colonic tissues of patients with UC, which highlights a potential role for HECTD3 in regulating inflammatory responses in UC. We identified MyD88, a central adaptor in the TLR/IL-1R signaling and inflammatory response, as a target for HECTD3 ubiquitination. We demonstrate that HECTD3 ubiquitinates MyD88 through K27-linked Poly-Ub chains in a nondegradative manner. HECTD3-KO GM-CSF–bone marrow–derived cells treated with the TLR4 ligand LPS produced more proinflammatory cytokines, showed elevated phosphorylation of NF-κB and IRAK4, as well as increased association of MyD88 with IRAK4, demonstrating that HECTD3 controls MyD88/IRAK4/NF-κB axis. Inhibition of MyD88 activity rescued colitis severity in the HECTD3-KO mice, including the elevated proinflammatory cytokine production. Thus, our results establish MyD88 as a target for HECTD3 nondegradative polyubiquitination and restriction of innate immune colonic inflammation.

## Abstract 2

Our study reveals how HECTD3 controls MyD88-driven immune signaling in the colon, helping limit excessive inflammation and protect against colitis.

## Graphical abstract

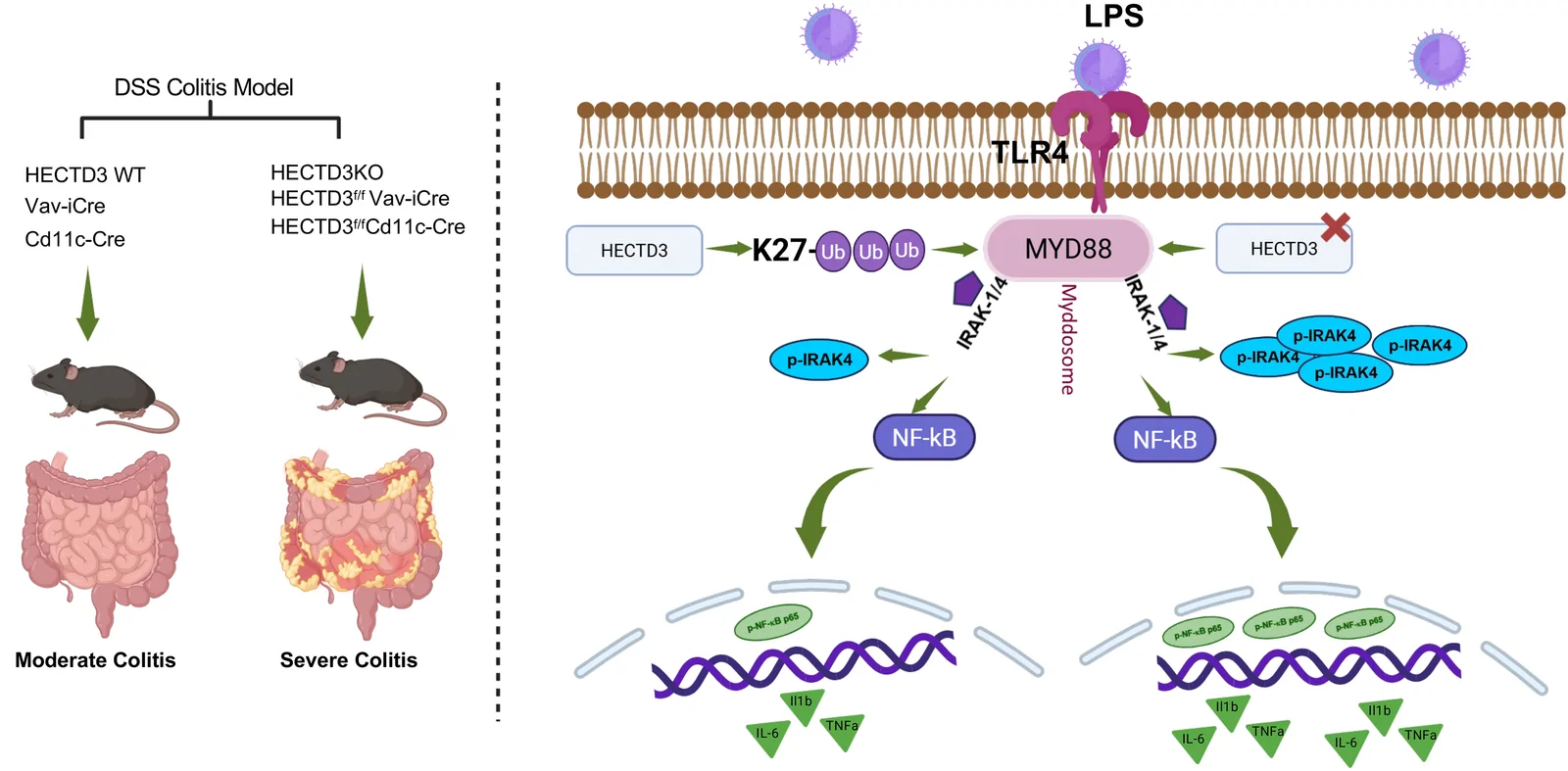

## Introduction

Ubiquitination is an important posttranslational modification that involves covalent attachment of 1 or more ubiquitin molecules to proteins, targeting them for proteasomal degradation, or conversely, facilitating association with other proteins, cellular relocation, or signaling responses (1, 2). Alteration in the ubiquitin system results in numerous diseases, including cancer, autoimmunity, and inflammation (3). Ubiquitination is initiated by E1 Ub activating enzymes, followed by E2 Ub-conjugating enzymes, and finalized by one of the over 600 E3 Ub ligases, that ultimately confers substrate specificity (4–7). The Homologous to E6-AP Carboxyl Terminus (HECT) E3 Ub ligases have intrinsic catalytic activity within the HECT domain (6, 8, 9). HECTD3 is a member of the HECT E3 ligase family that contains, in addition to the catalytic HECT domain at the C terminus, a substrate-recognizing destruction of cyclin (DOC) domain at the N terminus (10). Early studies in cell lines implicated HECTD3 in resistance to cisplatin-induced apoptosis and increased cancer cell survival through ubiquitination of caspases 8 and 9 (11–13). In addition, HECTD3 was shown to regulate glioblastoma tumorigenesis by ubiquitinating PARP1, promoting EGFR signaling (14).

Related to HECTD3’s role in immunity, we found that HECTD3 ubiquitinates STAT3 via K27 chains and MALT1 via both K27 and K29 chains in a nondegradative manner, and its deletion ablated Th17-dependent neuroinflammation in experimental autoimmune encephalomyelitis (EAE) (15). Other studies show that HECTD3 deficiency accelerated the clearance of RNA viruses and decreased inflammation by interfering with the function of double-stranded RNA-dependent protein kinase R (PKR) (16). Traf3 was identified as a target of HECTD3 in antibacterial response of macrophages (17), and recently, HECTD3 deletion was found to exacerbate disease severity in an NLRP3 inflammasome-dependent gouty arthritis model, however independent of the E3 Ub ligase activity (18).

In this study, we show that deletion of HECTD3 — germline or in hematopoietic compartment, including in Cd11c^+^ cells — led to heightened severity of dextran sodium sulfate–induced (DSS-induced) colitis, with elevated levels of the proinflammatory cytokines. We identified MyD88, a central adaptor in the TLR/IL-1R signaling pathways (19–22), as a target for HECTD3 nondegradative ubiquitination through K27-linked poly-Ub chains. LPS-treated HECTD3-KO GM-CSF–bone marrow–derived (GM-CSF-BMD) cells showed increased MyD88-IRAK4 interaction and phosphorylation of IRAK4 and NF-κB, suggesting increased activity of the MyD88/NF-κB axis. Further supporting our observations, administration of a MyD88 inhibitor alleviated disease severity and the elevated proinflammatory cytokines in HECTD3-KO mice colons. Highlighting a potential role for HECTD3 in regulating inflammatory responses in UC, *HECTD3* mRNA levels were found reduced in colonic tissues of patients with UC compared with disease-free tissue.

## Results

### HECTD3 mRNA is reduced in inflamed colonic mucosa of patients with UC and in several innate immune populations in mice with DSS colitis.

Analysis of publicly available data of patients with UC (23) pointed to a reduction of *HECTD3* mRNA levels in inflamed UC tissue versus normal tissue (Supplemental Figure 1A; supplemental material available online with this article; https://doi.org/10.1172/jci.insight.185437DS1). Further analysis of publicly available mouse transcriptomics datasets from mice with colitis following DSS treatment versus healthy mice (24) show that *Hectd3* mRNA is broadly expressed across multiple immune cell populations, including T cells, B cells, monocytes, macrophages, cDC1, inflammatory cDC2 (inf-cDC2) (25) and monocyte-derived dendritic cells (moDCs) (Supplemental Figure 1B). Interestingly, *Hectd3* expression was reduced in inf-cDC2, moDCs, and monocytes but not in T or B cells of mice with colitis (Supplemental Figure 1B), pointing to a potential role of HECTD3 in these innate populations in the context of intestinal inflammation. We conducted RNA-seq on Cd11c^+^ cells from intraepithelial (IE) compartment of HECTD3-KO versus WT mice with DSS-induced colitis. We did not include the myeloid cells, also with reduced *Hectd3* mRNA in colitis, given that this population was diminished in DSS colitis versus normal conditions (Supplemental Figure 1B). Pathway analyses show that IE Cd11c^+^ cells from HECTD3-KO mice have enrichment of gene sets associated with proinflammatory cytokines, leukocyte activation, innate immunity, TLR signaling, and pathways associated with positive regulation of NF-κB activity, similarly to pathways upregulated in patients with UC (Supplemental Figure 1C).

### HECTD3-KO mice develop severe DSS-induced colitis driven by the hematopoietic compartment.

To test the hypothesis that reduction of HECTD3 potentially contributes to colonic inflammation, we investigated the effect of HECTD3 deletion in DSS-induced colitis, which has been used as a murine model for the human UC (26, 27). Following DSS treatment (Figure 1A), HECTD3-KO mice showed significantly more weight loss and worsened histopathological scores compared with identically treated WT mice (Figure 1, B–D). To demonstrate the implication of the hematopoietic compartment in the more severe disease of HECTD3-KO mice, we conducted BM chimeras with donors derived from HECTD3-KO mice versus WT mice (Supplemental Figure 2). Our results show that WT recipients reconstituted with HECTD3-KO BMs display more weight loss and worsened histopathological scores compared with WT recipients reconstituted with WT BMs (Figure 2, A–C). Further demonstrating the contribution of the hematopoietic compartment, WT mice reconstituted with BMs from HECTD3^fl/fl^Vav-iCre mice showed more weight loss and worsened histopathological scores compared with mice reconstituted with BMs from Vav-iCre mice (Figure 2, D–F). HECTD3 was efficiently deleted in splenic germline and Hectd^fl/fl^Vav-iCre mice (Supplemental Figure 3, A and B). Thus, these experiments together demonstrate that germline deletion of HECTD3 causes increased colitis in the DSS model, and the hematopoietic compartment plays a central role in the disease.

### Proinflammatory cytokines are elevated in the colon of HECTD3-KO mice with DSS-induced colitis, predominantly in IE Cd11b^+^Cd11c^+^ cells.

We further evaluated proinflammatory cytokines in the colons of DSS-treated HECTD3-KO mice compared with WT and found that IL-1b, IL-6, and TNF-α were elevated in HECTD3-KO mice colons compared with WT (Figure 1E). To identify the immune cell populations responsible for increased cytokine production, we performed FACS analysis of immune cells isolated from the IE compartment. This analysis showed that the HECTD3-KO IE Cd11b^+^Cd11c^+^ cells exhibited increased levels of the proinflammatory cytokines IL-b, IL-6, and TNF-α compared with WT (Figure 1F). It was particularly the MHCII^hi^CD26^hi^CD64^–^ population, previously defined as inf-cDC2s (25), that shows consistent increase of the proinflammatory cytokines in the HECTD3-KO mice, and to a less extent the CD26^med^CD64^hi^ moDCs (Supplemental Figure 4, A–D). The frequencies of these populations, as well as of other immune populations, including B and T cells, were similar in the colon of HECTD3-KO and WT mice with colitis (Supplemental Figure 4E).

### Deletion of HECTD3 in Cd11c^+^ cells worsens DSS-induced colitis.

Given the elevated proinflammatory cytokine production observed in colonic Cd11c^+^ cells — particularly in inf-cDC2s and, at some extent, in moDCs — we next investigated whether Cd11c-dependent deletion of HECTD3, contributes to the aggravated colitis. We generated BM chimeras with BMs derived from Hectd3^fl/fl^Cd11c-Cre or Cd11c-Cre donor mice. Following DSS treatment, mice reconstituted with Hectd3^fl/fl^Cd11c-Cre BMs showed significantly greater body weight loss and more severe histopathological damage compared with those reconstituted with Cd11c-Cre BMs (Figure 2, G–I). In addition, Hectd3^fl/fl^Cd11c-Cre mice showed increased body weight loss and disease severity compared with Cd11c-Cre WT controls (Figure 2, J–L). Removal of HECTD3 was efficient in Hectd3^fl/fl^Cd11c-Cre BMD cells, ex vivo differentiated with GM-CSF (Supplemental Figure 3C).

FACS analysis further showed increased levels of the proinflammatory cytokines IL-1b, IL-6, and TNF-α in the colonic IE Cd11b^+^Cd11c^+^ cells from Hectd3^fl/fl^Cd11c-Cre mice with DSS-induced colitis, compared with Cd11c-Cre WT control mice — including in the inf-cDC2s population but not in CD26^med^CD64^hi^ moDCs (Figure 2M and Supplemental Figure 5, A–D) — similarly to the germline Hectd3-KO mice. These results demonstrate that deletion of HECTD3 in CD11c^+^ cells exacerbates DSS-induced colitis, and it is the inf-cDC2s population that shows elevated proinflammatory cytokines.

### Identification of MyD88 as a potential target for HECTD3.

Given the link of the disease phenotype with Cd11c-dependent depletion of HECTD3 and the increase in proinflammatory cytokines, we set up a system to generate ex vivo Cd11c^+^ cells derived from BM and then exposed them to inflammatory conditions to identify potential targets for HECTD3 ubiquitination in an unbiased manner, focusing on proteins associated with inflammatory pathways. Specifically, we differentiated BM cells in the presence of GM-CSF (GM-CSF-BMD cells). Such treatment results in enrichment of Cd11c^+^ cells, believed to resemble inflammatory DCs (28). Further GM-CSF-BMD cells were treated with fecal extracts from mice with DSS-induced colitis to expose them to the inflammatory conditions associated with the disease.

The protein extracts from HECTD3 KO and WT GM-CSF-BMD cells were digested by trypsin and were then enriched for peptides with the K-ε-GG remnants using anti-K-ε-GG antibodies before being subjected to mass spectrometry to identify differentially ubiquitinated peptides.

Following LC-MS/MS analysis and a global-scaling normalization, fold-change of normalized intensities were calculated. The ubiquitinated peptides were further filtered in WT-treated cells versus HECTD3-KO–treated cells, and further versus WT- and KO-untreated cells. The enriched ubiquitinated peptide-matched proteins were further subjected to pathway enrichment analysis using EnrichR (29). Ten pathways were identified in REACTOME database (Figure 3A), which were further clustered based on a Jaccard distance matrix using the pathway connectivity R shiny application (30). The analysis revealed 4 major clustered pathways: Innate Immune System, Infectious Disease, Cytokine Signaling, and Stress Response (Figure 3B). MyD88 was present in 3 of the 4 major pathway clusters: Innate Immune System, Infectious Disease, and Cytokine Signaling. Three peptides belonging to MyD88 were identified, one with the remnant K-ε-GG at K103 (R.LLELLALLDREDILK#ELK.S, identified 2 times) and located in the Myd88 death domain (DD), and a peptide with 2 K-ε-GG remnants at K119 and K127 (K.YLGK#QQNQESEK#PLQVAR.V), located in the intermediate domain (Figure 3, C and D). Given the central role of MyD88 in innate inflammatory response as a critical hub of signal integration and transduction (31–33), and the link with the 3 proinflammatory cytokines elevated in the colon of HECTD3-KO mice with colitis, we decided to focus on Myd88 and investigate the effect of HECTD3 on MyD88 ubiquitination.

### HECTD3 interacts with MyD88 in GM-CSF-BMD cells and promotes its polyubiquitination.

Given that MyD88 plays a critical role in signaling downstream of TLR4 and identification of enriched ubiquitinated MyD88 peptides in samples treated with fecal extracts, likely enriched in LPS, we treated GM-CSF-BMD cells with the TLR4 ligand LPS and conducted affinity purification of ubiquitinated proteins with Tandem Ubiquitin Binding Entities–agarose (TUBE-agarose). The results show enrichment of ubiquitinated MyD88 in LPS-treated GM-CSF-BMD WT versus HECTD3-KO cells (Figure 4A). Despite reduction in MyD88 ubiquitination in HECTD3-KO GM-CSF-BMD cells, levels of total MyD88 were the same between HECTD3-KO and WT GM-CSF-BMD cells and increased equally following LPS treatment (Figure 4B). Coimmunoprecipitation experiments show that endogenous HECTD3 and MyD88 associate in LPS-treated GM-CSF-BMD cells (Figure 4C). Thus, these results demonstrate that HECTD3 associates with MyD88 in LPS-treated GM-CSF-BMD cells and that MyD88 is polyubiquitinated in a HECTD3-dependent manner. *Absence of HECTD3 in GM-CSF-BMD cells increases NF-κB activation and proinflammatory cytokine production*. MyD88 serves as an adaptor that transmits signals initiated through TLRs, including TLR4, promoting NF-κB activation (19–22). We thus evaluated the phosphorylation of NF-κB p65 (P-Ser536) and found that LPS-treated HECTD3-KO GM-CSF-BMD cells exhibited higher p65 phosphorylation compared with WT control (Figure 4D). Additionally, the proinflammatory cytokines IL-1b, IL-6, and TNF-α were elevated in the LPS-treated HECTD3-KO GM-CSF-BMD cells compared with WT controls (Figure 4E), consistent with our observations in the colons of HECTD3-KO mice, including in the IE Cd11c^+^ cells. Thus, these results demonstrate that the absence of HECTD3 results in elevated activation of NF-κB and increased proinflammatory cytokine production in LPS-treated GM-CSF-BMD cells.

### HECTD3 directly ubiquitinates MyD88.

To examine in detail the HECTD3-dependent MyD88 polyubiquitination, we first generated HECTD3-KO HEK293T cells by CRISPR editing (Supplemental Figure 6). These cells were then transfected with Flag-MyD88 alone, Xpress-HECTD3 alone, or both Flag-MyD88 and Xpress-HECTD3. Western blot analysis demonstrated that anti-Xpress antibody specifically detected the ~100 kDa Xpress-HECTD3 protein and did not recognize the Flag-MyD88 (of ~35 kDa), whereas the anti-Flag antibody detected only the ~35 kDa Flag-MyD88 protein, but not the Xpress-HECTD3 (of ~100 kDa) (Supplemental Figure 7, A and B), demonstrating the absence of antibody cross-reactivity, similarly to what has been published with these antibodies (34-38).

Next HECTD3-KO and WT HEK293T cells were transfected with Flag-MyD88 and His-Ub, and the results show that, in the absence of HECTD3, MyD88 ubiquitination was reduced (Figure 5A). However, ectopic expression of WT HECTD3 in HECTD3-KO HEK293T cells restored MyD88 polyubiquitination, whereas the catalytically inactive HECTD3 C823A had a reduced effect (Figure 5, B and C). In addition, HECTD3 WT, but not the C823A mutant, promoted MyD88 ubiquitination in an in vitro ubiquitination system (Figure 5D).

In the ubiquitin remnant profiling, we identified K-ε-GG remnants at MyD88 K103, K119, and K127. We thus generated K→R mutants at each of these lysine residues to further evaluate HECTD3-dependent ubiquitination of MyD88. The results show reduction in ubiquitination for all K→R mutants, with the most pronounced effect at K103R (Figure 5E). Thus, these results collectively demonstrate that HECTD3 directly promotes ubiquitination of MyD88.

### HECTD3 promotes nondegradative polyubiquitination of MyD88 via K27 chains.

We first asked whether HECTD3-dependent polyubiquitination of MyD88 is associated with proteasomal degradation. Treatment with the proteasomal inhibitor MG132 did not affect MyD88 levels or its ubiquitination in cells transfected with HECTD3 (Supplemental Figure 8, A and B); however, it did promote an increase in MCL1, known to be ubiquitinated and targeted for proteasomal degradation (Supplemental Figure 8A). Thus, these results suggest that HECTD3-dependent ubiquitination does not result in MyD88 targeting for proteasomal degradation.

We next sought to determine the type of polyubiquitin chains that HECTD3 promotes on MyD88. Employing ubiquitin mutants that allow only specific lysine linkages, we found that the K27-only mutant was sufficient to promote ubiquitination of MyD88 in the presence of HECTD3 at similar levels as WT construct (Figure 6A). Conversely, HECTD3-mediated polyubiquitination was remarkably decreased in transfection with Ub-K27R mutant (Figure 6B). Collectively, these results show that HECTD3 mediates nondegradative polyubiquitination of MyD88 through K27-linked polyubiquitin chains.

### MyD88 and HECTD3 interact through the MyD88 DD and HECTD3 DOC domain.

We further investigated the association between MyD88 and HECTD3 in HEK293 cells following their transfection. The transfected full-length Xpress-HECTD3 associated with full-length Flag-MyD88 (Figure 7A), similarly to what we observed for the endogenous proteins (Figure 4C). MyD88 has 3 main domains: the N terminal domain, which comprises the DD (aa 54–109), important for the recruitment of IRAK family members and signal transduction; the C-terminal Toll–IL-1 receptor domain (TIR) (aa 159–296), and the intermediate domain (ID) (aa 110–155), which separates them (20, 39–42) (Figure 7B). Removal of the MyD88 DD domain, but not of the ID or of the TIR domains, abrogated the association with full-length HECTD3 (Figure 7C), while removal of the HECTD3 DOC domain, but not of the other domains, abolished the association of HECTD3 with MyD88 (Figure 7D). Thus, these results demonstrate that MyD88 and HECTD3 interact, and the interaction occurs through the MyD88 DD domain and the HECTD3 DOC domain.

### Absence of HECTD3 increases MyD88-IRAK4 association and IRAK4 phosphorylation.

MyD88 downstream signaling is initiated by MyD88 association with IRAK4 through interactions predominantly mediated by their DD domains. IRAK4 is activated by autophosphorylation, which further promotes activation of IRAK1 and IRAK2 and the assembly of the Myddosome, which further promotes the downstream signal transduction, ultimately activating NF-κB (43–49). A crystal structure of the heteromeric human MyD88-IRAK4-IRAK2 DD domain complex is available, consisting of 6 MyD88, 4 IRAK4, and 4 IRAK2 DDs (50). Directly mapping the identified mouse MyD88 ubiquitylation sites (K103, K119, and K127) onto this structure is challenging because K103 is not conserved between mouse and human, and K127 is outside of the resolved region of the structure. To address this, we generated a structural model by superimposing the AlphaFold-predicted mouse MyD88 DD domain onto the human MyD88 in Protein Data Bank (PDB) (ID: 3MOP) (50, 51). The 3 lysine residues (K103, K119, and K127) ubiquitylated by HECTD3 are highlighted (Figure 8A). MyD88 DD forms 2 types of interfaces in the helical assembly: the MyD88-MyD88 interface and the MyD88-IRAK4 interface. K103 is involved in both interfaces. Ubiquitylation of K103 by HECTD3 is anticipated to disrupt complex formation and diminish signaling output. K119 and K127, located within the MyD88-MyD88 interface, are also critical. Since the MyD88 helical assembly serves as a scaffold for IRAK4 and IRAK2 recruitment, ubiquitylation at K119 and K127 by HECTD3 is expected to negatively affect IRAK4/2 kinase recruitment and activation. Therefore, our structural modeling suggests that K103, K119, and K127 are crucial within the DD domain interface of the MyD88/IRAK4/IRAK2 complex, with their ubiquitylation likely leading to reduced NF-κB activation. In support of this hypothesis, coimmunoprecipitation experiments show that MyD88-IRAK4 interaction was more pronounced and the phosphorylation of IRAK4 was increased in LPS-treated HECTD3-KO GM-CSF-BMD cells compared with WT (Figure 8, B–D).

We further generated an experimental system in HEK293T cells to test the effect of HECTD3 on the interaction of MyD88 with IRAK4. HECTD3-KO HEK293T cells were cotransfected with Flag-MyD88 and Myc-IRAK4, as well as with TLR4 together with the adaptor MD-2 and CD14, to have all components necessary for MyD88-IRAK4 pathway activation, as previously described (52, 53). In addition, either WT HECTD3 or the catalytically inactive C823A HECTD3 mutant were transfected. Transfection with WT HECTD3, but not with the C823A HECTD3 mutant, reduced the association of IRAK4 with MyD88 (Figure 8, E and F).

These results together demonstrate that absence of HECTD3 increases MyD88 association with IRAK4 and IRAK4 phosphorylation.

### Inhibition of MyD88 activity rescued increased DSS-induced colitis severity in HECTD3-KO mice, including the proinflammatory cytokines.

To determine to what extent the elevated MyD88 activation is responsible for the increased DSS-induced colitis severity in HECTD3-KO mice, we treated mice with DSS-induced colitis with the MyD88 inhibitor NBP2–29328 peptide (MyD88 I) or the control antennapedia peptide (CP) (Figure 9A). The results show that MyD88 I, but not the CP, improved the weights and restored the disease scores of the HECTD3-KO mice to the level of WT (Figure 9, B–D). In addition, the levels of the proinflammatory cytokines IL-1b, IL-6, and TNF-α were dampened to the levels of WT mice in the HECTD3-KO mice treated with the MyD88 I but not with the CP (Figure 9E). These results together demonstrate that MyD88 plays a central role in the elevated colitis severity of the HECTD3-KO mice.

## Discussion

In this study, we found that germline deletion of HECTD3, as well as hematopoietic deletion, including Cd11c-dependent deletion, resulted in increased DSS-induced colitis severity, resulted in increased DSS-induced colitis severity. The increased disease severity in HECTD3-KO mice was associated with increased production of the proinflammatory cytokines (IL-1b, IL-6, and TNF-α), particularly by the colonic CD26^hi^CD64^–^ inf-cDC2s cells. The MHCII^hi^CD26^hi^CD64^–^ population was previously defined as inf-cDC2s in respiratory viral infection and shown to have increased ability to present antigens, while the CD26^med^CD64^hi^ moDCs lack that ability (25).

HECTD3 deficiency has been previously associated with enhanced host defense against intracellular bacterial infections, an effect mediated through type I IFN response via K63-linked polyubiquitination of TRAF3 (17). It was also shown that HECTD3 inhibits NLRP3 inflammasome activation by disrupting the interaction between NEK7 and NLRP3, independent of its E3 ligase activity (18). Here, we identified MyD88 in an unbiased manner as a target for nondegradative ubiquitination of HECTD3. Using ubiquitin remnant proteomics coupled with stringent filtering to enrich for HECTD3-dependent ubiquitination events, MyD88 emerged as a strong candidate among other inflammatory signaling proteins. Notably, 2 independent ubiquitinated peptides mapping to the death and intermediate domains of MyD88 were detected. The localization of these modifications within domains required for IRAK recruitment provided a mechanistic basis for the observed increase in MyD88/IRAK4 complex formation and downstream NF-κB activation in HECTD3-KO cells. Furthermore, the inflammatory cytokine signature observed in colitic HECTD3-KO mice and activated GM-CSF-BMD cells was highly consistent with enhanced MyD88-dependent signaling. The unbiased identification of MyD88 by ubiquitin remnant profiling was subsequently validated through biochemical, genetic, and pharmacological approaches, including in mice, establishing MyD88 as a major functional substrate through which HECTD3 restrains intestinal inflammation.

These findings were further supported by experiments demonstrating that absence of HECTD3 in GM-CSF-BMD cells treated with LPS increased not only the proinflammatory cytokines and phosphorylation of NF-κB, but also phosphorylation of IRAK4 and its association with MyD88, demonstrating that HECTD3 constrains inflammation through control of MyD88 and downstream signal transduction through IRAK4. Importantly, inhibition of MyD88 rescued the increased disease severity of HECTD3-KO mice, as well as the colonic proinflammatory cytokines. MyD88 deficiency has been shown to result in complex outcomes, sometimes conflicting. Initial studies show that MyD88-KO mice developed more severe DSS-induced colitis, suggesting that MyD88 signaling plays a protective role in maintaining intestinal homeostasis and mucosal integrity during DSS-induced injury. Loss of MyD88 signaling impaired innate immune protective mechanisms in the gut, leading to exacerbated intestinal inflammation and tissue damage, suggesting that appropriate innate immune activation through MyD88 is essential for controlling colitis severity and maintaining mucosal integrity (54). However, it was further demonstrated that blocking MyD88 signaling with an inhibitor prevented colitis-associated colorectal cancer (55). Additionally, this MyD88 inhibitor was recently successfully used in a special delivery system to treat DSS-induced colitis, blocking colonic inflammation (56). In addition, inhibition of IRAK4, which is downstream of MyD88 in the signaling cascade, was shown to abrogate colitis-induced neoplasm (57).

Furthermore, MyD88-KO mice were shown to be resistant to *Heligmosomoides polygyrus* infection. Additionally, absence of Myd88 protected Foxp3-deficient mice from skin, lung, and gut disease but not systemically (58, 59). Moreover, cell-specific MyD88 deletion confers protection in certain cells. Keratinocyte-specific deletion of MyD88 limits skin inflammation, and macrophage-specific deletion reduces hepatic injury and fibrosis, while myofibroblast-specific KO attenuates liver tumorigenesis and fibrosis (60–62). Notably, in the colitis model associated with IL-10 deficiency, MyD88 is required for disease development, as IL-10^–/–^ MyD88^–/–^ double-knockout mice, as well as mice with cell-specific MyD88 deletion in myeloid or Cd11c^+^ cells, do not develop spontaneous colitis, demonstrating that loss of MyD88 can protect from intestinal inflammation (34). Thus, MyD88 activation and downstream signaling mechanisms and their effects are complex. Excessive or cell-specific MyD88 signaling can drive pathogenic inflammation in specific cells, including myeloid and Cd11c^+^ cells, and its targeted inhibition can reduce it.

In this study, we propose that HECTD3 modulates MyD88 activation and signaling. Our results show that HECTD3-dependent nondegradative polyubiquitination of MyD88 occurs at K103, K119, and K127, all of which located in the MyD88 DD and participating in distinct interface interactions. Structural modeling suggests that HECTD3-dependent polyubiquitination at K103 may directly interfere with the interaction with IRAK4. HECTD3-dependent polyubiquitination at K119 and K127, located at the MyD88-MyD88 interface, may impair MyD88 self-assembly and is expected to also negatively affect IRAK4/2 recruitment and activation. In support of such model, in the absence of HECTD3, we observed increased MyD88-IRAK4 association and elevated phosphorylation of IRAK4 in LPS-treated HECTD3-KO BMD cells.

A recent structural study showed that HECTD3 preferentially uses UBE2D family and forms short polyubiquitin chains, predominantly K11-linked and, to a lesser, extent K48 (63). Based on this, Huber et al. hypothesized that HECTD3 may function as a “seed” ubiquitin ligase, initiating ubiquitin modifications that are subsequently extended by additional ubiquitin ligases. In addition, the study analyzed in depth the in vitro ubiquitination of PARP1 by K48-linked ubiquitin chains, even though HECTD3-dependent ubiquitination of PARP1 was shown by a previous study to be K63-chain mediated and not K48 (14). Consistent with observations that HECTD3 generates short ubiquitin chains, our in vitro ubiquitination assay produced short ubiquitinated MyD88 species. In addition, the cell-based assays also revealed relatively short molecular-weight ubiquitinated forms that were K27-linked, similar to our previous findings for STAT3 and MALT1 (15).

MyD88 was previously shown to be polyubiquitinated at residues located in the TIR domain, namely at K231 and K262, by the Smurf E3 ligases, which caused its degradation downstream of TGF-β signaling, dampening the TLR proinflammatory signaling (64). MyD88 was also found to be ubiquitinated and targeted for proteasomal degradation by the E3 ubiquitin ligase Speckle-type BTB-POZ protein (SPOP). SPOP deletion in mice resulted in systemic inflammation and hyperinflammatory-like syndrome. It was proposed that SPOP restricts MyD88-dependent inflammation through its targeting for proteasomal degradation (65). Importantly, HECTD3-dependent ubiquitination of MyD88 does not trigger its proteasomal degradation, despite the effect on dampening inflammation. Other ubiquitin ligases have been found to regulate MyD88 and control inflammation, including Cbl-b (66), Nrdp1 (67, 68), and Smurf1 (69), while the deubiquitinases CYLD, OTUD4, and USP3 were found to remove K63-linked polyubiquitination on MyD88 (70–72).

Our results on the role of HECTD3 on MyD88 ubiquitination and control of inflammation associated with DSS-induced colitis are in contrast to the amelioration of EAE and CD4^+^ T cell–associated inflammation in the absence of HECTD3 (15), suggesting that HECTD3 acts in a cell type– and tissue-specific manner to exert its roles. In pathogenic Th17 cells, HECTD3 ubiquitinated Malt1 and Stat3 in a nondegradative manner, promoting differentiation of pathogenic Th17 cells.

In summary, our results show that HECTD3 exerts a protective effect by diminishing the colonic innate immune inflammation through ubiquitination of MyD88 in a nondegradative manner, reducing the association with IRAK4 and dampening MyD88 downstream signaling.

## Methods

### Sex as a biological variable.

We are presenting data only for the male mice, as they exhibit a more consistent response to DSS-induced colitis compared with the female mice, as previously published (73). For generation of GM-CSF-BMD cells, both males and females were used.

### Mice.

HECTD3-KO mice were previously described (15). These mice have been crossed on C57BL/6 for 10 generations. The WT control mice derived from heterozygous HECTD3-KO/WT mice.

HECTD3^fl/fl^ mice were generated using CRISPR-Cas9 system with guide RNAs designed by Sial (Sigma-Aldrich) based on a predictive algorithm (74). Three guide RNAs were cloned into the U6-gRNA-only transfection plasmid and cotransfected with Cas9 to a pool of cells from a model cell line, tested for targeting efficiency and further tested via Surveyor assay. The most active gRNA was further manufactured. The microinjection was conducted into 150 C57BL/6 zygotes (100 ng/μL Cas9 mRNA, 50 ng/μL single guide RNAs (sgRNA/sgRNA) and 50 ng/μL of donor (ssDNA). Thirty-six pups were born and genotyped, and we identified 1 founder with F/wt (floxed/wild-type) at the 5′ loxP site, and F/F at the 3′ loxP site. The founder was further crossed to generate HECTD3^fl/fl^ mice and backcrossed on C57BL/6 for over 5 generations.

The validated gRNA sequence UpHECTD3 is TTTTAGTCACACGCCCCCTAGG; the validated gRNA sequence DownHECTD3 is GGACATCCGCTCAATACCCAGG. The Donor Oligo Sequence (Key: LoxP cassette, *EcoRI restriction enzyme sequence*, Coding sequence, CRISPR gRNA target sequence, Left Homology, Right Homology) is 5′ aaggggtcattctaccaaaca ttttagtcacacgccccct ATAACTTCGTATAATGTATGCTATACGAAGTTAT *GAATTC* agg atagttgaacaatttttataagatttctcaagcctgg 3′; the Donor Oligo Sequence (Key: LoxP cassette, *EcoRI restriction enzyme sequence*, Coding sequence, CRISPR gRNA target sequence, Left Homology, Right Homology) is 5′ gttcttccagaggcttggcat ggacatccgctcaataccc ATAACTTCGTATAATGTATGCTATACGAAGTTAT *GAATTC* agg ctttaccagcagccaaactactgtcttctggatcata 3′.

HECTD3^fl/fl^ mice were crossed with Vav-iCre (strain no. 008610, The Jackson Laboratory) and CD11cCre (strain no. 007567, The Jackson Laboratory). WT controls used in the current version have been Vav-iCre or CD11cCre, respectively. In DSS-colitis experiments, only male mice aged 8–14 weeks, were used, given that they show a more consistent phenotype. In ex vivo differentiation BM cells with GM-CSF, both males and females were used.

### Generation of BM chimeric mice.

Recipient WT CD45.1 mice (4–5 weeks old) were treated with antibiotic water containing sulfamethoxazole/trimethoprim for 2 weeks prior to BM transplantation and maintained on sulfamethoxazole/trimethoprim for 2 weeks after transfer. One day before transplantation, recipient mice were lethally irradiated with 2 doses of 500 rads separated by a 4-hour recovery interval. BM cells were harvested from donor mice (5–6 weeks old): HECTD3 KO, HECTD3^fl/fl^ Vav-iCre, and HECTD3^fl/fl^ Cd11c-Cre and the corresponding WT, Vav-iCre, and Cd11c-Cre controls. Each CD45.1 recipient mouse was reconstituted with 1 × 10^7^ BM cells. Experiments were conducted 8 weeks after reconstitution

### Acute DSS colitis.

In total, 1.5% Dextran Sulfate Sodium (DSS) (catalog 160110), MP Biomaterials) was administered in drinking water for 7 days, with the exception of the MyD88 inhibitor (MyD88 I) experiment when the administration was for 10 days. Following DSS treatment, mice were switched to regular drinking water and then euthanized on day 10 or 11 for the MyD88 I experiment. MyD88 I and the antennapedia control (CP) (NBP2-29328, Novus Biologicals) were i.p. injected (100 μM) on days 3, 5, 7, and 10 (75).

### Histological analysis.

Colon tissue specimens were collected, flushed with PBS, and cut longitudinally. Colons were then “Swiss rolled” (76) and fixed overnight at 4°C in 10% formalin (catalog HT501128, Sigma-Aldrich), embedded in paraffin, and stained with H&E. Image analysis was performed using Aperio Image scope, ImageJ (NIH), and Leica Biosystems. A Histopathological scoring system was adapted and modified from the previous study (77), using the following criteria: Score 1 = no damage, no goblet cell loss, no inflammatory cell infiltrate; Score 2 = minimal damage, minimal goblet cell loss, minimal inflammatory cell infiltrates; Score 3 = moderate damage, moderate goblet cell loss and moderate cell infiltration; and Score 4 = Extensive damage, extensive goblet cell loss, extensive inflammatory cell infiltrates.

### Multiplex immunoassay of colon explants.

Colons were harvested, flushed with PBS, and cut longitudinally, and the most distal colon 2 cm piece was resuspended in lysis buffer (150 mM NaCl, 1% NP-40, and 20 mM Tris), containing protease inhibitors, and homogenized with 1 mm diameter disruption beads in a Benchmark Beadbug microtube homogenizer at 400 rpm for 45 seconds, 3 times. Lysates were centrifuged at 10,000*g* for 10 minutes at 4°C, and supernatants were collected. Cytokine levels were analyzed using the multiplex Immunoassay (Multiplex Mouse Cytokine/Chemokine Array 32-Plex; Eve Technologies) and normalized to mg of protein.

### Flow cytometry analysis of cytokines in the colonic IE compartment.

To detect intracellular cytokines, 250 μg of Brefeldin A (BioLegend, 420601) was injected per mouse, 4 hours before euthanasia. The harvested colon specimens were flushed with PBS, cut longitudinally, and then washed in 0.5% DTE solution at 37°C with agitation at 220 rpm for 20 minutes twice. Percoll separation (44%/66%) was used to isolate IE immune cells. Cells were stained for CD45.2, CD11b, CD11C, F4/80, MHCII, CD86, CD26, CD64, TCRβ, and B220 (Supplemental Table 2), before being fixed and permeabilized with eBioscience FOXP3//Transcription Factor Fixation and /Permeabilization buffer (Invitrogen,00-5521-00) and stained for IL-1b, IL-6, and TNF-α (Supplemental Table 2). Data were acquired on the Cytek Aurora Flow cytometer and analyzed by FlowJo (TreeStar).

### Cells.

HEK293T cells (CRL-3216, ATCC) were grown in DMEM (Cytiva DMEM/High glucose, catalog SH3024301) plus 10% FBS and 1% penicillin-streptomycin. *E*. *coli* strain MAX Efficiency DH5α competent cells (catalog 18258012, Invitrogen, Thermo Fisher Scientific) were used for transformation and preparation of plasmid.

### CRISPR/Cas9 KO of HECTD3 in HEK293 cell line.

The guide RNA (gRNA) sequence CATCAACCGCCGTCTTGCCA targeting human HECTD3 exon 10 was cloned into the lentiCRISPR v2 (78) (catalog 52961, Addgene). Lentiviruses were generated using 293T packaging cells following the protocol “Lentivirus production” (DOI: 10.21769/BioProtoc.39). HEK293T cells were transduced, and single-cell clones were isolated following puromycin selection and further screened by WB analysis for HECTD3.

### Vector constructs.

HIS-Ub WT, HIS-Ub K-only (K6, K11, K27, K29, K33, K48, K63, K0), and K27R mutant constructs were previously generated as described (79). pcDNA3.1-Xpress-HECTD3 WT and -C823A mutant were generated by deletion of the 6xHis from the corresponding pcDNA3.1/HisC HECTD3 WT and C823A mutant clones (15). pCMV2-Flag-MyD88 was purchased from Addgene (plasmid no. 13093) and used for generation of domain deletion mutants by site directed mutagenesis. HECTD3 domain deletion mutants were previously described (15). For MyD88-IRAK4 interaction experiments, mouse IRAK4 c-Myc (plasmid no. MG57925-CM) from Sino Biological, pMSCV-IRES-GFP-MD2 (F126A)-HA (plasmid no.133067), pcDNA3-CD14 (plasmid no. 13645), and pcDNA3-TLR4-YFP (plasmid no. 13018) were obtain from Addgene.

### Site-directed mutagenesis for point mutation and truncation.

The Q5 Site-directed mutagenesis Kit (catalog E0554S, New England Biolabs) was used to generate all mutants according to the manufacturer’s protocol. The primers used are listed in Supplemental Table 1.

### Transfection.

Transfection of HECTD3-KO and WT HEK293T cells was performed using Polyethylenimine (PEI), Polysciences (catalog 23966-2). In brief, cells were seeded in 10 cm plates at 60%–70% confluence. Plasmid DNA was diluted with serum-free DMEM, and 3 volumes of PEI (1 μg/μL) was added followed by incubation at room temperature for 20 minutes. The DNA-PEI complexes were then added to each 10 cm plate dropwise.

### Protein extraction.

Protein extraction was performed as previously described (15) in the presence of P8340 protease inhibitor cocktail (catalog P8340, MilliporeSigma), 1 mM NaF phosphatase inhibitor, 1 mM Na3VO4·2H2O phosphatase inhibitor, 1 mM β-glycerophosphate phosphatase inhibitor, cOmplete EDTA-free protease inhibitor cocktail (catalog 11873580001, Roche Diagnostics), and the deubiquitinases inhibitor PR-619 (catalog SI9619, LifeSensors). For His-Ub Ni-NTA pull-down (PD) experiments, 90% of the cells were resuspended in Buffer A (6M Guanidine hydrochloride, 0.1M Na_2_HPO_4_/NaH_2_PO_4_, 10 mM imidazole) followed by ultrasonication (ampl 50%) for 30 seconds. The remaining 10% (input) was resuspended in lysis buffer (50 mM Tris-Cl [pH 7.4], 150 mM NaCl, 1% Triton X-100, 1 mM EDTA) supplemented, followed by ultrasonication for 15 seconds. Both fractions were centrifuged at 15,700*g* for 15 minutes at 4°C and supernatants were collected.

### Ni-NTA PD.

In brief, 40 μL HisPur Ni-NTA (catalog 88221, Thermo Scientific) slurry was incubated with 800 μL protein lysate, obtained as above in, at room temperature for 2–3 hours with rotation. Then the slurry was washed twice with Buffer A (6M guanidine hydrochloride, 0.1M Na_2_HPO_4_/NaH_2_PO_4_, 10 mM imidazole), followed by 2 more washes with Buffer TI (25 mM Tris-HCl [pH 6.8], 20 mM imidazole) and buffer A, 3:1 mixture, and a final wash with buffer TI only. The PD-ed proteins were then eluted from the slurry by boiling with urea sample buffer (120 mM Tris HCl [pH 6.8], 4%SDS, 4M Urea, 20% Glycerol) and were subjected to immunoblot analysis.

### Ubiquitinated protein enrichment.

In total, 1 μg of protein extract from BMD cells was enriched for ubiquitinated proteins with TUBE2 Agarose (catalog UM402, LifeSensors), following the manufacturer’s protocol.

### Immunoblot (Western blot) analysis.

Protein samples were separated by 10%–12% sodium dodecyl sulfate-polyacrylamide gel electrophoresis (SDS-PAGE) under reducing conditions and subsequently transferred onto 0.2 μm polyvinylidene difluoride (PVDF) membranes. Membranes were blocked with 5% skim milk in Tris-buffered saline containing 0.1% Tween-20 (TBS-T) for 1 hour at room temperature and then incubated overnight at 4°C with the following primary antibodies: IRAK4 (1:1,000, catalog 4363), phospho-IRAK4 (Thr345/Ser346, 1:1,000, catalog 11927), MyD88 (1:1,000, catalog 4283), NF-κB p65 (1:1,000, catalog 8242S), phospho-NF-κB p65 (Ser536, 1:1,000, catalog 3033S), Lamin A/C (1:2,000, catalog 4777S), GAPDH (1:1,000, catalog 2118L), and MCL-1 (1:1,000, catalog 4572) from Cell Signaling Technology, as well as HECTD3 (1:2,000, catalog A304-924A, Bethyl Laboratories). Following 3 washes with TBS-T, membranes were incubated with HRP-conjugated secondary antibodies (1:4,000) for 1 hour at room temperature with gentle agitation. Secondary antibodies included Peroxidase AffiniPure Goat Anti-Mouse IgG (catalog 115-035-003), Goat Anti-Rabbit IgG (catalog 111-035-003), and Goat Anti-Rat IgG (catalog 112-035-003) (Jackson ImmunoResearch). After additional washes with TBS-T and TBS, protein bands were detected using Lumi-Light Western Blotting Substrate (Roche) or SuperSignal West Femto Maximum Sensitivity Substrate (Thermo Fisher Scientific). Chemiluminescent signals were captured using a ChemiDoc Touch Gel Imaging System (Bio-Rad Laboratories) and analyzed with Image Lab software (Bio-Rad). In some experiments, membranes were stripped using Restore Western Blot Stripping Buffer and reproved with additional antibodies.

### Immunoprecipitation.

Immunoprecipitation was performed as previously described (15), using Protein A Sepharose (PAS) CL-4B (catalog 17096303, GE Healthcare Bio-Sciences) and MyD88 (catalog 4283, Cell Signaling Technology), anti-Xpress antibody (catalog R910-25, Invitrogen, Thermo Fisher Scientific), or anti-Flag M2 antibody (catalog F1804, MilliporeSigma).

### In vitro ubiquitination of Flag-MyD88.

In vitro Ubiquitination was performed on purified Flag-MyD88 from transfected HEK293 cells. Flag-MyD88 was purified on ANTI-FLAG M2 Affinity Gel (45-A2220, MilliporeSigma). Approximately 150 ng Flag-MyD88 was incubated with 5 μM E1 enzyme (catalog E-306-050, R&D systems), 25 μM E2 enzyme (Recombinant Human UbcH5a/UBE2D1) (catalog E2-616-100 R&D systems), and 10 μM purified GST-HECTD3 WT or GST-HECTD3823A mutant, in E3 Ligase Reaction buffer (50 mM HEPES [pH 8.0], 50 mM NaCl, 1 mM TCEP), plus 1.17 mM Ubiquitin (catalog U-110-01M, R&D systems) and 100 mM Mg-ATP solution (catalog U-110-01M, R&D systems). The reaction was conducted at 37°C for 60 minutes. Ubiquitin conjugation profiles were analyzed by immunoblot analysis. GST-HECTD3 WT and GST-HECTD3823A, cloned in PGEX-6P-1, were expressed in Rosetta (DE3) cells. The proteins were purified on Glutathione Sepharose 4B, eluted in the presence of reduced glutathione, and further added to the reaction.

### In vitro GM-CSF-BMD cell differentiation.

BM cells were isolated from femurs of HECTD3-KO and WT mice and cultured in RPMI 1640 (Gibco) media with 10% FBS, 1% penicillin-streptomycin, and 25 ng GM-CSF/mL. The media were refreshed on day 3. On day 7, cells were treated with 100 ng/mL of LPS (*E*. *coli* O111:B4, L2630 Sigma-Aldrich) for 3 hours or with fecal extracts overnight.

### Fecal extracts.

Feces were collected on day 9 after DSS treatment. Equal amounts of feces (1–2 pellets) from both WT and HECTD3-KO–treated mice were combined and resuspended in 500 μL of sterile PBS, plus 200 μL of 1.0 mm silica beads (Thermo Fisher Scientific, NC9847287) and further homogenized on the bead beater (Benchmark Scientific, D1030) at 4000 rpm for 90 seconds. After homogenization, the samples were spun down, and the supernatants were collected, mixed with sterile glycerol, and frozen at –80°C.

### Ubiquitin remnant profiling, LC-MS/MS), and pathway analysis of ubiquitinated proteins.

Ubiquitin Remnant profiling of protein lysates from BMD-cells was performed using the PTMScan Ubiquitin Remnant Motif (K-ε-GG) Kit (catalog 5562, Cell Signaling Technology) according to manufacturer’s protocol. The samples were analyzed by LC-MS/MS at the Harvard Taplin Mass Spectrometry Facility. Peptide sequences and protein identities were determined by matching protein databases with the acquired fragmentation pattern using Sequest (Thermo Fisher Scientific) (80). For ubiquitination searches, a mass addition of 114.04293 Da on lysine residues was included.

Peptide intensities within each protein were derived from the raw data, and average peptide intensities were calculated for each peptide across technical replicates. A global-scaling normalization was applied, and fold changes of the normalized intensities were then calculated.

Next we filtered the enrichment of ubiquitinated peptides (1.5-fold and higher) in the WT-treated versus WT-untreated samples. Further filtering was applied based on a difference in log2 fold change greater than 2 between treated WT/KO and untreated WT/KO, resulting in 1,289 peptides matched to 724 proteins. These were further subjected to pathway enrichment analysis using EnrichR in the REACTOME gene set database (https://reactome.org/; accessed 2024/03/15). The top 10 pathways were further clustered based on the pathway connectivity based on a Jaccard distance matrix (30).

### Data availability.

The RNA-seq data generated in this study have been deposited in the NCBI Gene Expression Omnibus (GEO) under accession no. GSE280228 and are publicly available at: https://www.ncbi.nlm.nih.gov/geo/query/acc.cgi?acc=GSE280228

All graphs presented in this study and their corresponding source data are provided in the Supporting Data Values file. Uncropped Western blot images are available in the file Hectd3_manuscript_uncropped_WB.pptx. The Graphical abstract was created using BioRender.

### Statistics.

For single comparisons, unpaired 2-tailed Student’s *t* test was used. For multiple comparisons, 2-way ANOVA followed by Šídák’s or Tukey’s multiple-comparisons test was used, as specified in the corresponding figure legend. Unless otherwise specified, *P* ≤ 0.05 was considered significant.

### Study approval.

All mouse studies were approved by the IACUCs of University of Florida, University of South Florida, and Moffitt Cancer Center.

## Author contributions

Conceptualization was contributed by SI, SM, AZ, UP, VBC, PCR, MM, CJ, MJF, JJC, OAG, AAB, ZR, LW, HL, TIS, and DA. Methodology was contributed by SI, SM, AZ, UP, JJC, AJK, LS, VBC, MJF, SYK, RT, MGMG, XL, HL, ZR, TIS, and DA. Investigation was contributed by SI, SM, AZ, UP, JJC, DPT, TD, TZ, AJK, LS, VBC, MJF, SYK, RT, AN, and ZR. Visualization was contributed by SI, SM, AZ, UP, SC, RPS, SCV, DPT, JJC, VBC, MJF, SYK, RT, TIS, ZR, and DA. Funding acquisition was contributed by CJ, OAG, BGK, HL, and DA. Data analysis was contributed by SI, SM, AZ, UP, JJC, VBC, MJF, SYK, RT, TIS, ZR, and DA. Data curation was contributed by SI, SM, AZ, UP, RT, TIS, ZR, and DA. Writing was contributed by SI, SM, AZ, UP, VBC, ZR, MM, MJF, JJC, AAB, ZR, LW, TIS, and DA.

## Conflict of interest

The authors have declared that no conflict of interest exists.

## Funding support

This work is the result of NIH funding, in whole or in part, and is subject to the NIH Public Access Policy. Through acceptance of this federal funding, the NIH has been given a right to make the work publicly available in PubMed Central.

- NIH R01AI067846 to DA and HL.
- R01DK121831 to OG.
- Moffitt Cancer Center Grant P30CA076292 to DA and TIS.
- Merit Society award to DA and Miles for Moffitt to DA and LW.

## Supplementary Material

Supplemental data

Unedited blot and gel images

Supporting data values

## Figures and Tables

**Figure 1:**
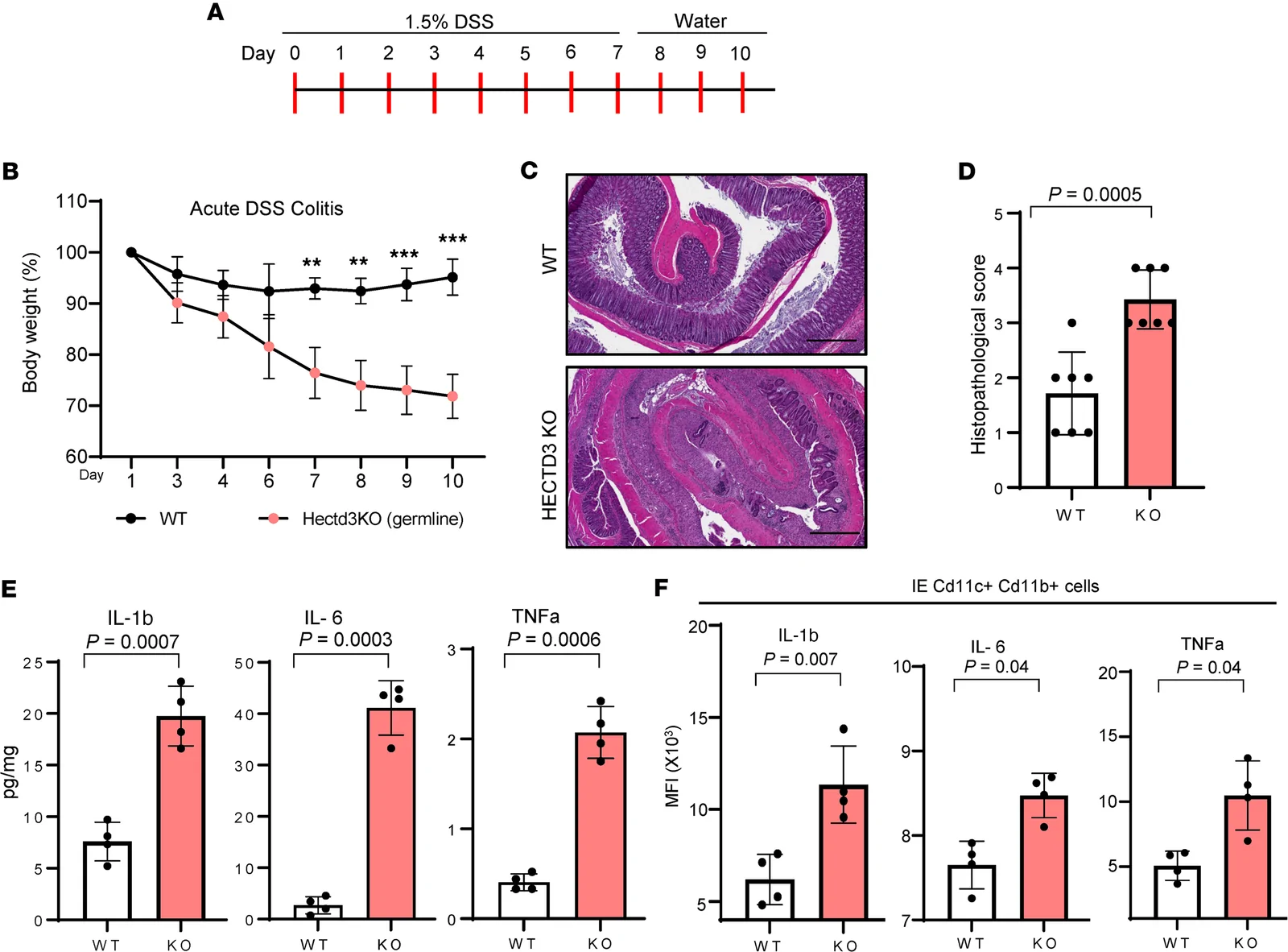
HECTD3-KO mice develop more severe disease in the DSS-induced colitis model. (**A**) Experimental model for acute DSS-induced colitis. Mice are treated with 1.5% DSS for 7 days followed by 3 days of recovery. On day 10, mice are euthanized for analysis. (**B**) Percent body weight changes from the initial weight of HECTD3-KO and WT mice following DSS treatment (*n* = 7/group). **P < 0.001; ***P < 0.0005. (**C** and **D**) Representative H&E staining (**C**) and histopathological scores based on criteria presented in Methods (*n* = 7/group) (**D**). Scale bar: 300 μm. (**E**) Multiplex cytokine (IL-1b, IL-6, TNF-α) analysis of colonic samples from HECTD3-KO and WT mice treated with DSS (*n* = 4). (**F**) Mean fluorescence intensity (MFI) of the indicated cytokines (IL-1b, IL-6, TNF-α) by FACS analysis in the Cd11c^+^Cd11b^+^ cells from the colon intraepithelial (IE) fraction of HECTD3-KO and WT mice treated with DSS (*n* = 4/group). In **B**, *P* values were determined by 2-way ANOVA followed by Šídák’s multiple-comparison test. For **D**–**F**, *P* values were determined by unpaired 2-tailed Student’s *t* test. Data are shown as mean ± SEM.

**Figure 2:**
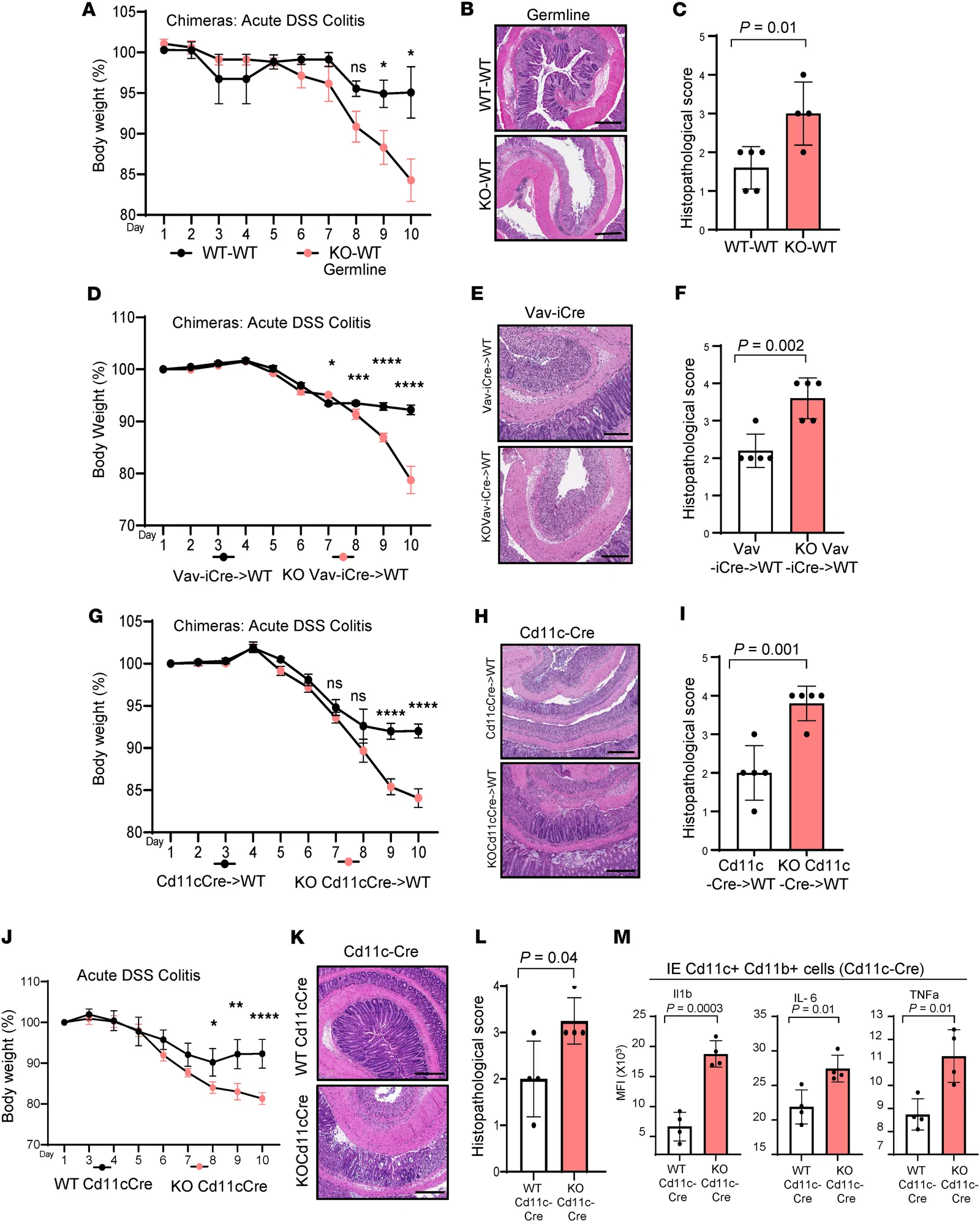
HECTD3 deficiency in hematopoietic system including in CD11c^+^ cells exacerbates DSS-induced colitis. (**A**–**L**) Percent body weight changes from the initial weight and histopathological scores for the indicated groups of mice treated with DSS colitis. (**A**–**I**) Bone marrow chimeras. (**A**–**C**) Donors: germline Hectd3 KO and WT. (**D**–**F**) Donors: Hecdt3^fl/fl^Vav-iCre and Vav-iCre. (**G**–**I**) Donors: HECTD3^fl/fl^Cd11c-Cre and CD11c-Cre. (**A**, **D**, and **G**) Percent body weight changes from initial body weight in the indicated chimera groups treated with 1.5% DSS for 7 days, followed by regular drinking water (*n* = 5/group) (*P < 0.05, ***P < 0.0005; ****P < 0.0001). *P* values by 2-way ANOVA followed by Šídák’s multiple-comparison test. (**B**, **E**, and **H**) Representative H&E-stained colon sections from each group. Scale bars: 300 μm. (**C**, **F**, and **I**) Histopathological scores based on criteria presented in Methods (*n* = 5/group). *P* values by Student’s unpaired *t* test. Data are presented as mean ± SEM. (**J**–**L**) Acute DSS-induced colitis in HECTD3^fl/fl^Cd11c-Cre and control Cd11c-Cre mice. (**J**) Percent body weight changes from the initial weight following 1.5% DSS treatment (*n* = 4/group). *P < 0.05; **P < 0.001; ****P < 0.0001. *P* values by 2-way ANOVA followed by Šídák’s multiple-comparison test. (**K**) Representative H&E-stained colon sections from each group. Scale bars: 300 μm. (**L**) Histopathological scores based on criteria presented in Methods (*n* = 4/group). (**M**) MFI for the indicated cytokines (IL-1b, IL-6, TNF-α) by FACS analysis in the Cd11c^+^Cd11b^+^ cells from the colonic IE fraction (*n* = 4/group). (**L** and **M**) *P* values by Student’s unpaired *t* test. Data are presented as mean ± SEM.

**Figure 3:**
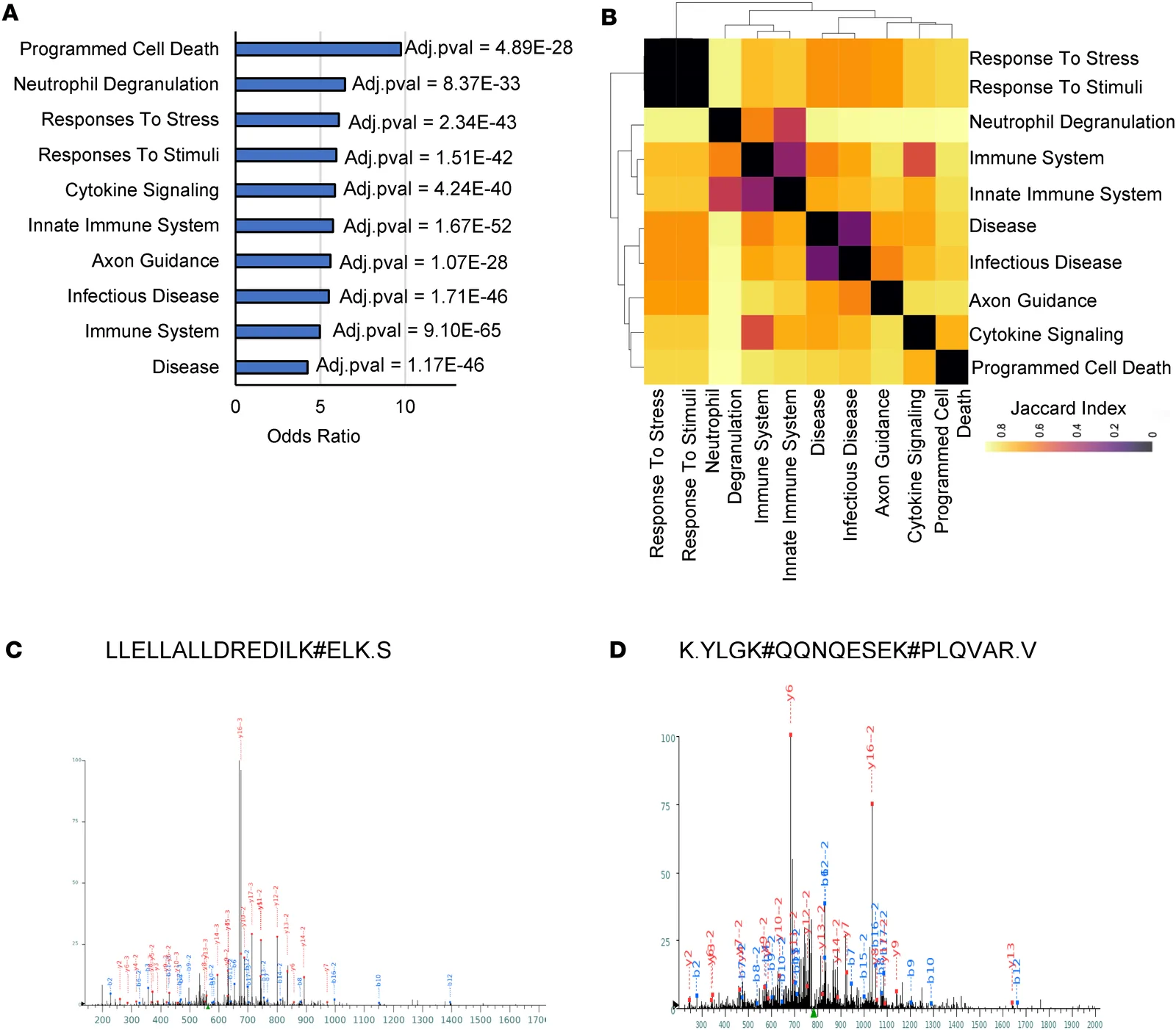
Ubiquitin remnant profiling and mass spectrometry analysis of extracts from HECTD3-KO and WT GM-CSF-BMD cells treated with fecal extracts from mice with colitis. (**A**) Bar plot showing the odds ratio of enriched REACTOME pathways performed by EnrichR. (**B**) Heatmap clustering performed via the pathway connectivity. Jaccard similarity index was calculated based on shared proteins enriched in each of the top 10 REACTOME pathways. (**C** and **D**) Fragmentation spectra of ubiquitinated LLELLALLDREDILK#ELKS peptide (K103 residue) and ubiquitinated KYLGK#QQNQESEK#PLQVARV peptide (K119 and K127 residues) of MyD88. Parent ions corresponding to LLELLALLDREDILK#ELKS and KYLGKQQNQESEKPLQVARV peptide masses have been subjected to higher-energy collision dissociation in mass spectrometer. The detected b-type (N-terminal) and y-type (C-terminal) fragment ion series generated by peptide backbone cleavage have been annotated.

**Figure 4:**
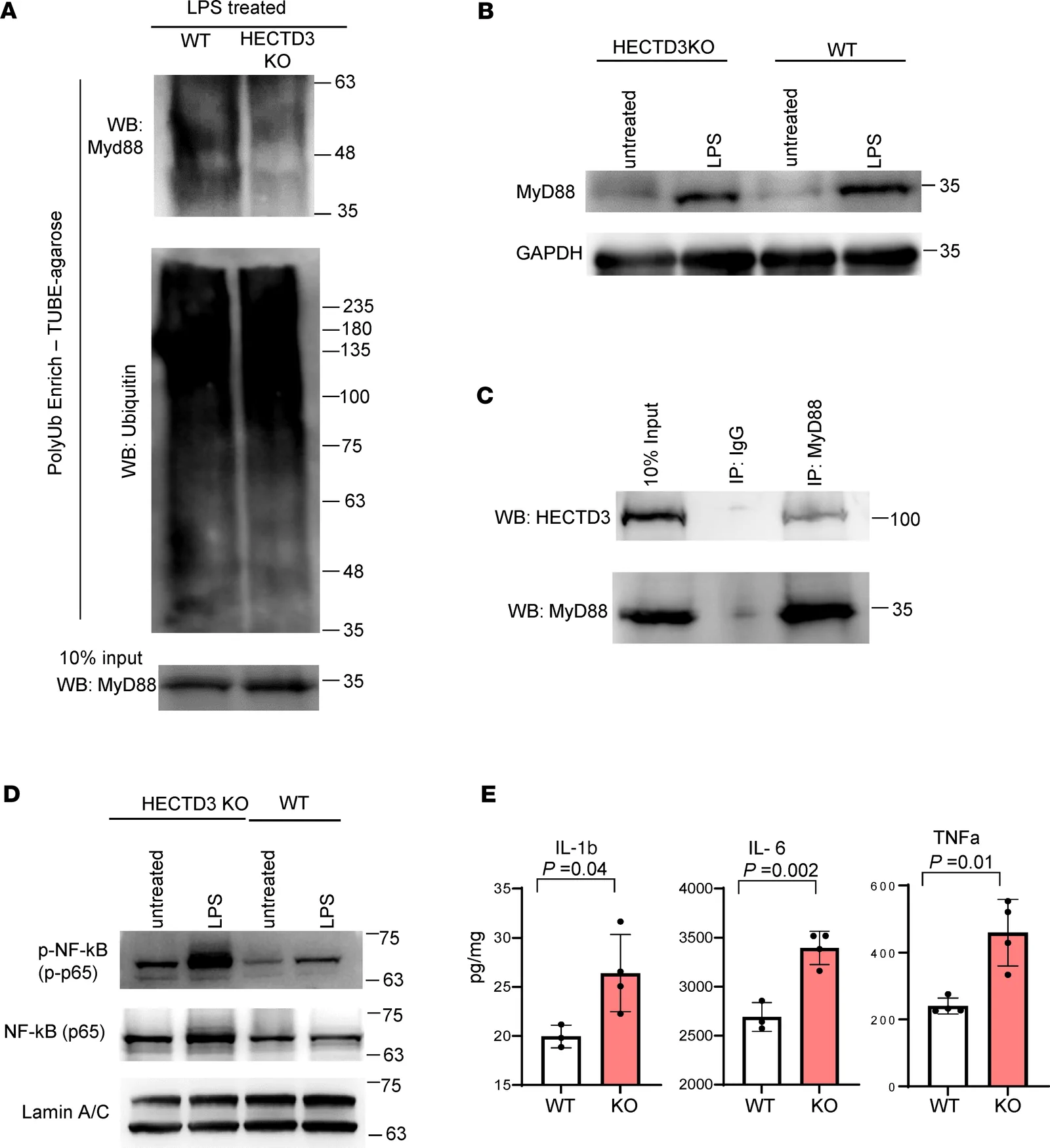
HECTD3 interacts with MyD88 and promotes its polyubiquitination dampening NF-κB activation and proinflammatory cytokine production in GM-CSF-BMD cells treated with LPS. (**A**) Immunoblot with anti-MyD88 and anti-ubiquitin antibodies following enrichment of polyubiquitinated proteins with TUBE-agarose. Protein extracts were derived from HECTD3-KO and WT GM-CSF-BMD cells treated with LPS. (**B**) Immunoblot for MyD88 and GAPDH in extracts from GM-CSF BMD cells treated with LPS. GM-CSF BMD cells derived from WT and HECTD3-KO mice. Data are representative of 2 independent experiments. (**C**) Immunoblot for HECTD3 and MyD88 following immunoprecipitation with anti-MyD88 or IgG antibodies of total protein extracts from HECTD3-KO and WT GM-CSF-BMD cells treated with LPS. (**D**) Immunoblot of p-NF-κB (p65), NF-κB (p65), and Lamin A/C in nuclear extracts from LPS-treated HECTD3-KO and WT GM-CSF-BMD cells. (**E**) Multiplex cytokines analysis for IL-1b, IL-6, and TNF-α (*n* = 4/group) in media of cultured GM-CSF-BMD cells treated with LPS (*n* = 4). (**A**–**D**) Data are representative of 2 independent experiments. Data are presented as mean ± SEM. *P* values by Student’s unpaired *t* test.

**Figure 5:**
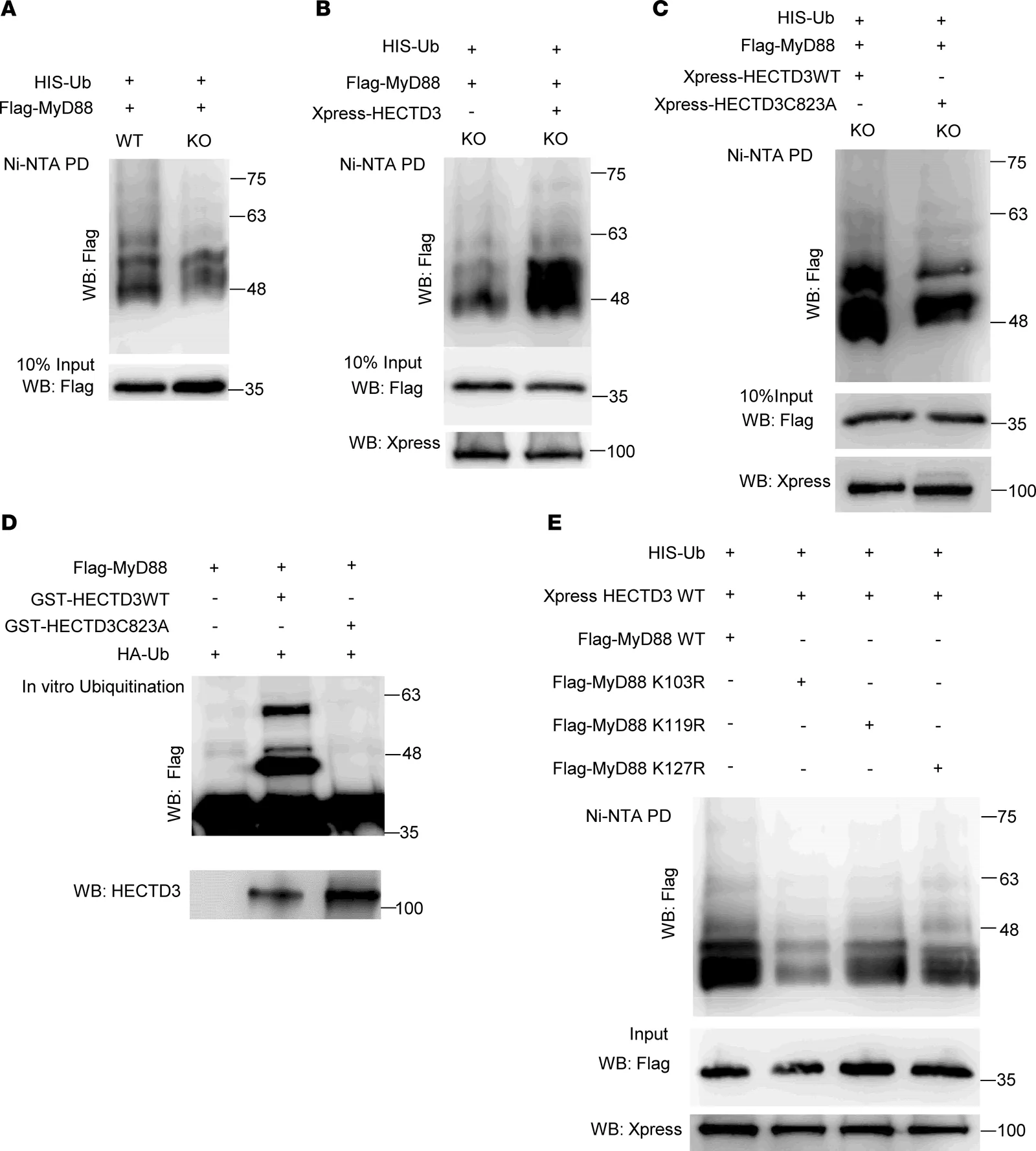
Molecular details of HECTD3-dependent ubiquitination of MyD88. (**A**) Anti-Flag immunoblot of Ni-NTA pulled-down (PD) protein extracts from WT and HECTD3 KO HEK293T cells cotransfected with HIS-Ub and Flag-MyD88. (**B** and **C**) Anti-Flag immunoblot of Ni-NTA PD protein extracts from HECTD3 KO HEK293T cells cotransfected with HIS-Ub, Flag-MyD88, and Xpress-HECTD3 WT (**B**) or Xpress-HECTD3 C823A (**C**). Input is shown for Flag-MyD88 and Xpress-HECTD3 (WT in **B** and HECTD3 WT and HECTD3 C823A mutant in **C**). (**D**) Anti-Flag immunoblot of in vitro ubiquitinated Flag-MyD88. Flag-MyD88 was incubated with E1 enzyme, E2 enzyme, HA-Ub and GST-HECTD3 WT, or GST-HECTD3 C823A mutant. (**E**) Anti-Flag immunoblot of Ni-NTA PD protein extracts from HECTD3 KO HEK293T cells cotransfected with HIS-Ub, Xpress-HECTD3, Flag-MyD88 WT, Flag-MyD88 K103R, Flag-MyD88 K119R, or Flag-MyD88 K127R. Data are the representative of the 2 independent experiments.

**Figure 6:**
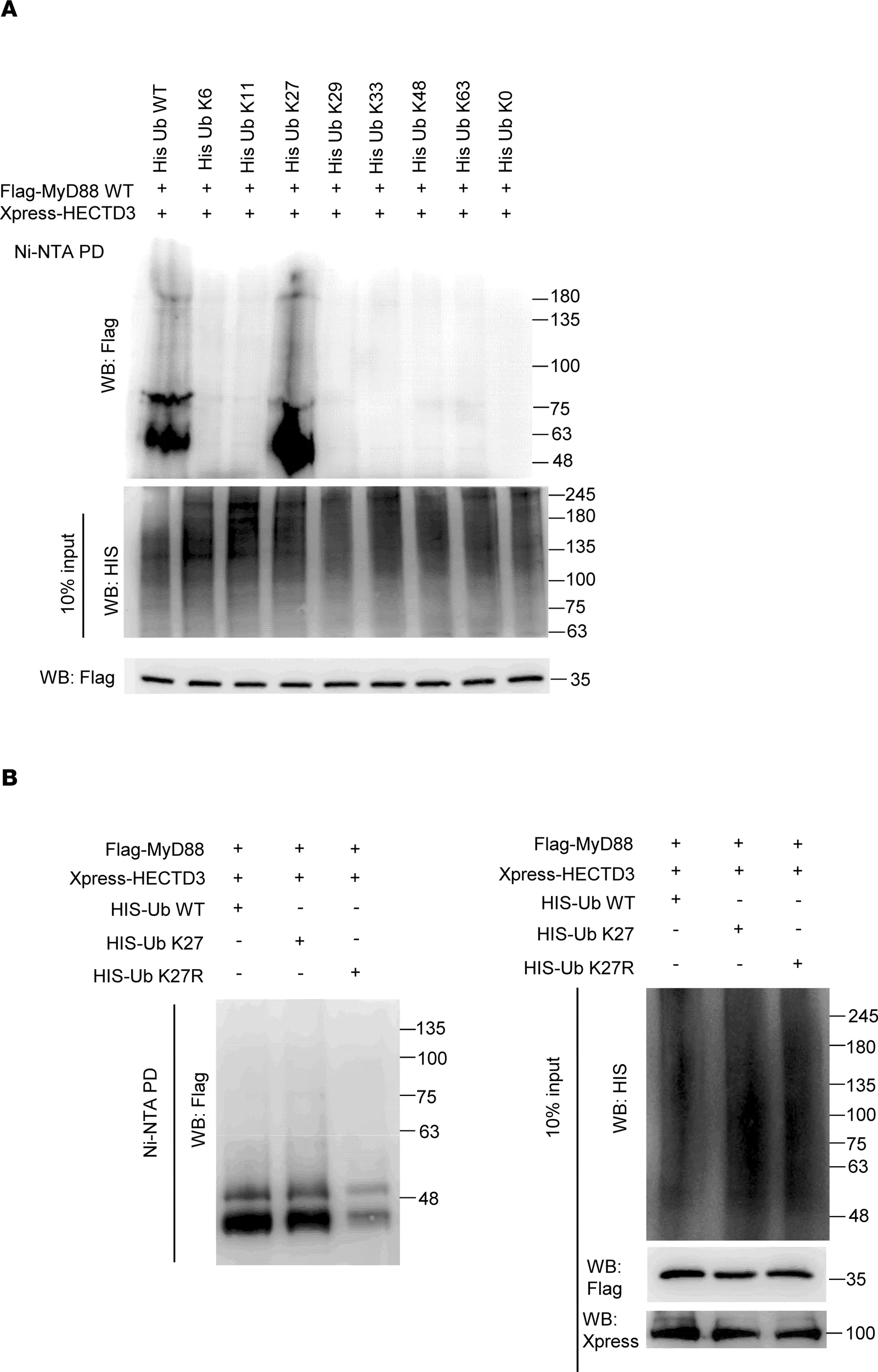
HECTD3 promotes polyubiquitination of MyD88 through K27-linked chains. (**A**) Anti-Flag immunoblot of Ni-NTA PD protein extracts from HECTD3 KO HEK293T cells cotransfected with Flag-MyD88, Xpress-HECTD3 and HIS-Ub WT or HIS-Ub K only mutants. HIS-Ub K only mutants denote that the only K in the HIS-Ub is at the indicated residue, and all other K residues are mutated to R. (**B**) Anti-Flag immunoblot of Ni-NTA PD protein extracts from HECTD3-KO HEK293T cells cotransfected with Flag-MyD88, Xpress-HECTD3 and HIS-Ub WT or HIS-Ub K27 ONLY or HIS-Ub K27R mutant. Data are representative of the 2 independent experiments. Input is shown for His-Ub, Flag-MyD88, and Xpress-HECTD3.

**Figure 7:**
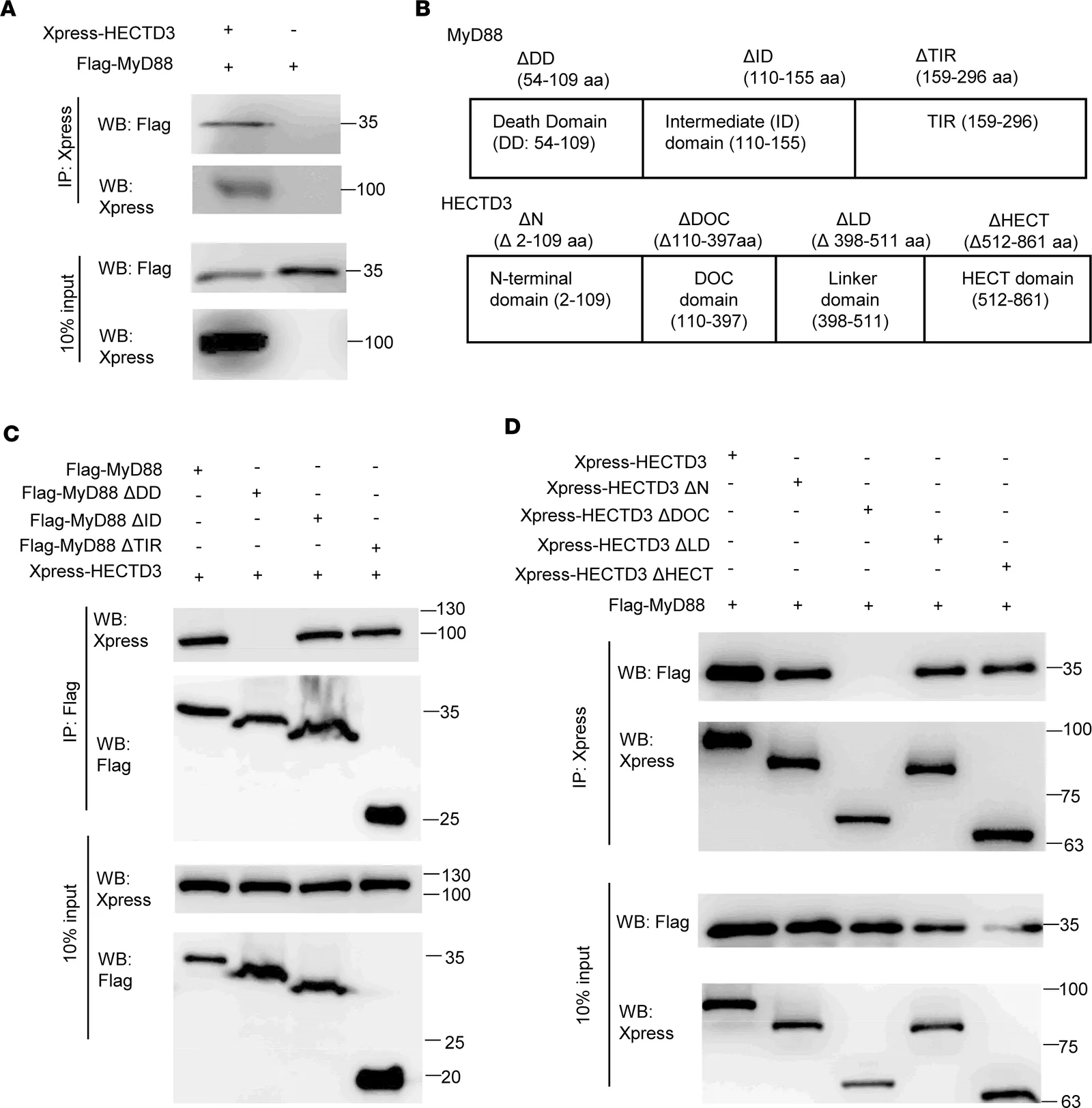
MyD88 death domain and HECTD3 DOC domain mediate the interaction between MyD88 and HECTD3. (**A**) Xpress and anti-Flag immunoblots of anti-Flag immunoprecipitated proteins from HECTD3-KO HEK293T cells cotransfected with Flag-MyD88 and Xpress-HECTD3. (**B**) MyD88 and HECTD3 domain representation. (**C**) Anti-Xpress and anti-Flag immunoblots of anti-Flag immunoprecipitated proteins from HECTD3-KO HEK293T cells cotransfected with Xpress-HECTD3 and Flag-MyD88 or Flag-MyD88 ΔDD, Flag-MyD88 ΔID, and Flag-MyD88 ΔTIR mutants (**D**) Anti-Xpress and anti-Flag immunoblots of anti-Flag–immunoprecipitated proteins from HECTD3-KO HEK293T cells cotransfected with Flag-MyD88 and either Xpress-HECTD3 or the Xpress-HECTD3 ΔN, ΔDOC, ΔLD, or ΔHECT mutants. Data are the representative of the 2 independent experiments.

**Figure 8:**
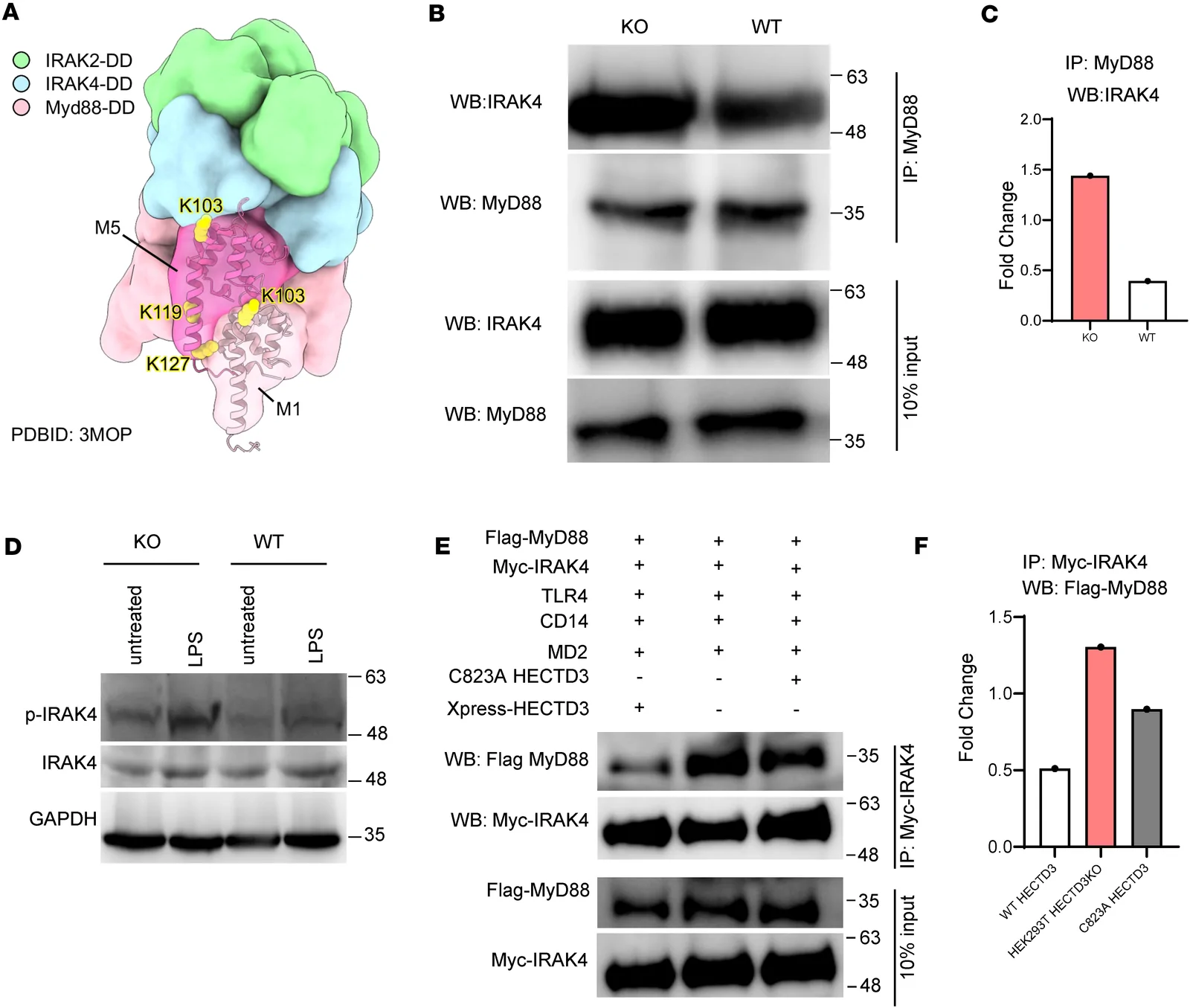
Absence of HECTD3 causes increased MyD88-IRAK4 interaction and IRAK4 phosphorylation. (**A**) A structural model of MyD88-IRAK4-IRAK2 DD domain complex based on PDBID 3MOP. Individual DD domain of MyD88 (pink), IRAK4 (blue), and IRAK2 (green) are shown in surface representation. Two illustrated models of mouse MyD88 DD domain are superimposed into the M1 and M5 units in the helical assembly. The location of K103, K119, and K127 are indicated using yellow spheres. (**B**) Immunoblot for IRAK4 and MyD88 following MyD88 immunoprecipitation of protein extracts from HECTD3-KO and WT GM-CSF-BMD cells treated with LPS. (**C**) Quantification of IRAK4 from **B**. (**D**) Immunoblot of p-IRAK4, IRAK4, and GAPDH from HECTD3-KO and WT GM-CSF-BMD cells treated with LPS. (**E**) HECTD3 ubiquitin ligase activity restricts constitutive MyD88-IRAK4 complex formation. HEK293T HECTD3-KO cells were cotransfected with Flag-MyD88, Myc-IRAK4, TLR4, CD14, and MD2, together with either WT HECTD3 (first lane) or the catalytically inactive HECTD3 mutant C823A (third lane). HECTD3-KO cells served as control (second lane). Cell lysates were subjected to immunoprecipitation with anti-Myc-IRAK4 followed by immunoblotting to assess MyD88-IRAK4 complex formation. (**F**) Quantification of Flag-MyD88 from **E**. (**B**–**F**) Data are representative of 2 independent experiments.

**Figure 9:**
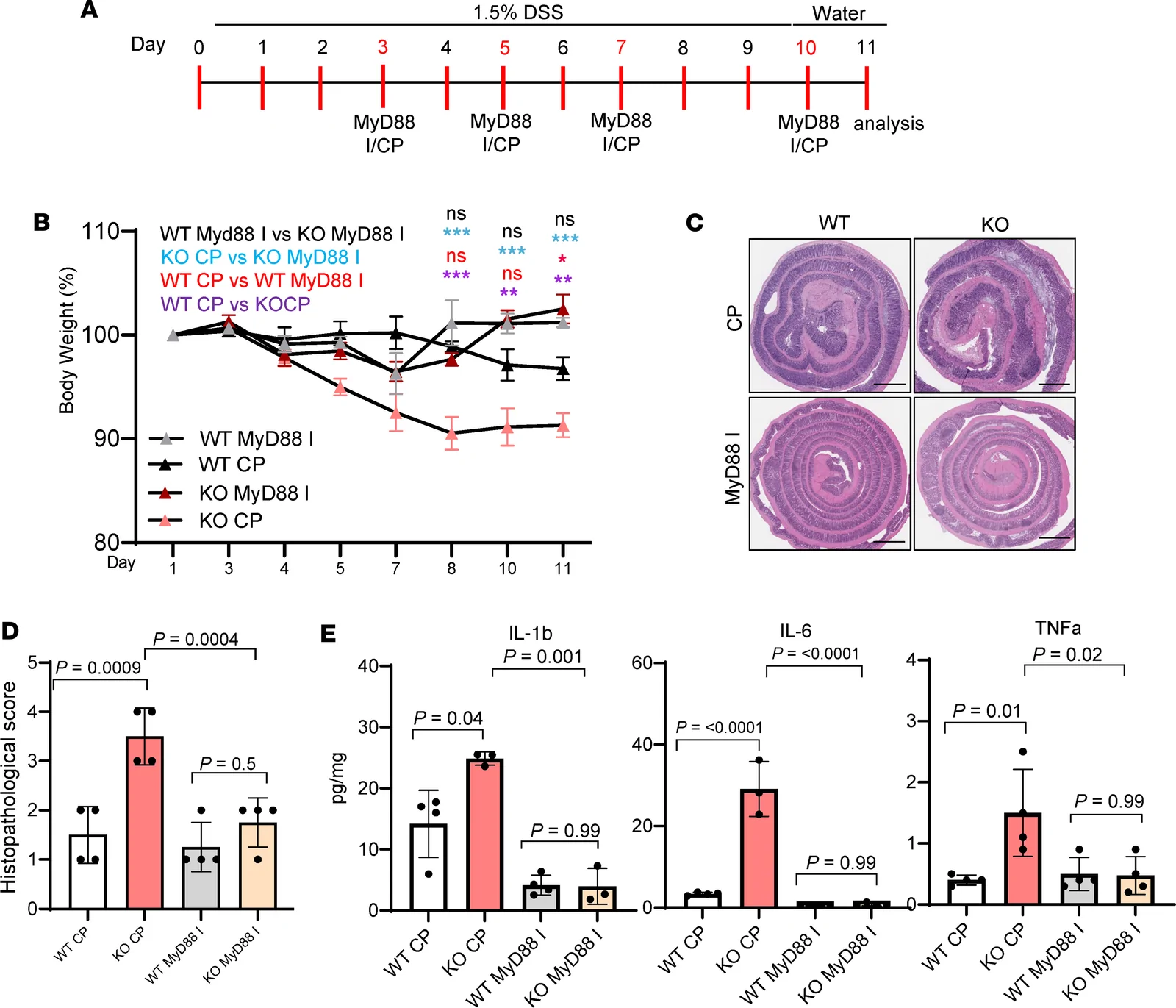
Inhibition of MyD88 activity rescues the exacerbation of DSS colitis in HECTD3-KO mice. (**A**) Experimental design. HECTD3-KO and WT mice were administered orally with 1.5% DSS for 10 days. On Days 3, 5, 7, and 10, mice were injected i.p. with the MyD88 inhibitor peptide (NBP2-29328) (MyD88 I) or control antennapedia peptide (CP). On day 11, mice were euthanized for analysis. (**B**) Percent body weight change from the initial weight of HECTD3-KO and WT mice following DSS treatment plus MyD88 I or CP (*n* = 4/group) (*P < 0.05; **P < 0.001; ***P < 0.0005). *P* values were calculated by 2-way ANOVA, followed by Šídák’s multiple-comparison test. (**C** and **D**) Representative H&E staining and histopathological scores (*n* = 4/group). Scale bar: 500 μm. (**E**) Multiplex cytokine analysis of IL-1b, IL-6, and TNF-α in colonic samples from HECTD3-KO and WT mice treated with MyD88 I or CP (*n* = 4/group). (**D** and **E**) Data are presented as mean ± SEM. *P* values by 2-way ANOVA followed Tukey’s multiple-comparison test.

## Version 1 09/24/2026

In-Press Preview

## Version 2 10/22/2026

Electronic publication
