# Supplementary material for: The E3 ubiquitin ligase HECTD3 controls immune colonic inflammation by restricting MyD88 activation and signaling
Source: JCI Insight. 2026 Sep 24;11(20):e185437. doi: 10.1172/jci.insight.185437 (PMC13726568; doi:10.1172/jci.insight.185437)
Supplement: Supplementary file 1 [file jciinsight-11-185437-s062.pdf]

Figure S1

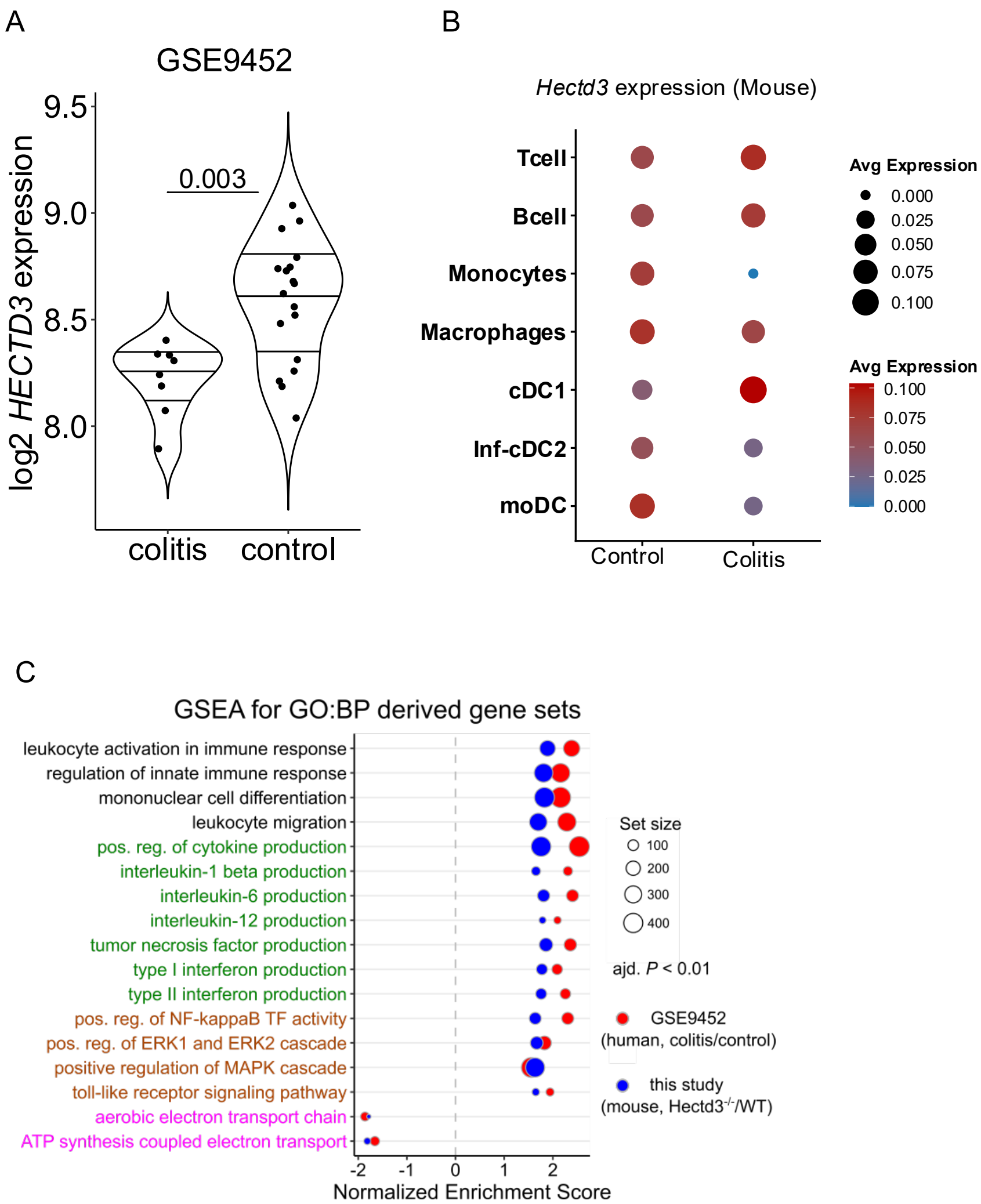
A
GSE9452
log2 HECTD3 expression
9.5
9.0
8.5
8.0
0.003
colitis
control
B
Hectd3 expression (Mouse)
Tcell
Bcell
Monocytes
Macrophages
cDC1
Inf-cDC2
moDC
Control
Colitis
Avg Expression
0.000
0.025
0.050
0.075
0.100
Avg Expression
0.100
0.075
0.050
0.025
0.000
C
GSEA for GO:BP derived gene sets
leukocyte activation in immune response
regulation of innate immune response
mononuclear cell differentiation
leukocyte migration
pos. reg. of cytokine production
interleukin-1 beta production
interleukin-6 production
interleukin-12 production
tumor necrosis factor production
type I interferon production
type II interferon production
pos. reg. of NF-kappaB TF activity
pos. reg. of ERK1 and ERK2 cascade
positive regulation of MAPK cascade
toll-like receptor signaling pathway
aerobic electron transport chain
ATP synthesis coupled electron transport
Set size
100
200
300
400
ajd. P < 0.01
GSE9452
(human, colitis/control)
this study
(mouse, Hectd3-/-/WT)
-2
-1
0
1
2
Normalized Enrichment Score

Figure S1. HECTD3 expression is reduced in inflamed colon of patients with ulcerative colitis (UC) and in colonic innate immune cells of mice with DSS colitis. (A) *HECTD3* mRNA in inflamed colonic mucosa samples from UC patients versus non-inflamed controls (log2 transformed microarray values). *p* values by unpaired Student's t test assuming equal variance. Data analyzed from GSE9452. **(B)** *HECTD3* mRNA expression across different mouse immune cell populations, including T cells, B cells, monocytes, macrophages, cDC1, inflammatory cDC2 (inf-cDC2) and monocyte-derived (moDCs). **(C)** Gene Set Enrichment Analysis (GSEA) using GO:BP-derived gene sets on ranked differentially expressed genes between inflamed colonic mucosa samples from patients with UC and non-inflamed controls retrieved from GSE9452, and between Hectd3-KO and WT murine $Cd11c^{+}$ cells from the colon of mice treated with DSS. Murine Hectd3-KO cells display deregulation of similar biological processes as the inflamed colonic mucosa samples from ulcerative colitis human patients. Four categories are highlighted, including upregulated innate immune response (black), cytokine production (green), NF-kappa B signaling (brown) and downregulated aerobic electron transport chain (magenta). All data points have FDR adjusted *p* values <0.01.

Figure S2

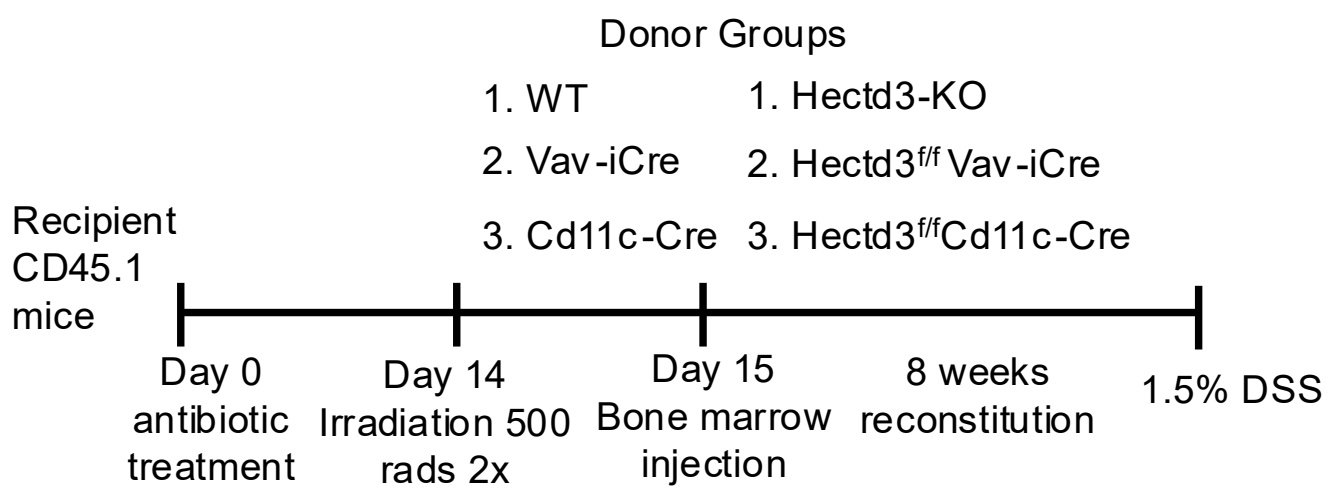
Donor Groups
1. WT
2. Vav-iCre
3. Cd11c-Cre
1. Hectd3-KO
2. Hectd3f/f Vav-iCre
3. Hectd3f/fCd11c-Cre
Recipient CD45.1 mice
Day 0 antibiotic treatment
Day 14 Irradiation 500 rads 2x
Day 15 Bone marrow injection
8 weeks reconstitution
1.5% DSS

Figure S2. Experimental design for bone marrow chimeras and DSS treatment to induce acute colitis post reconstitution. Recipient mice are CD45.1. Donors for the three experimental groups are indicated: (1) germline Hectd3-KO and WT, (2) Hectd3$^{f/f}$Vav-iCre and Vav-iCre; (3) Hectd3$^{f/f}$Cd11c-Cre and Cd11c-Cre, all CD45.2.

Figure S3

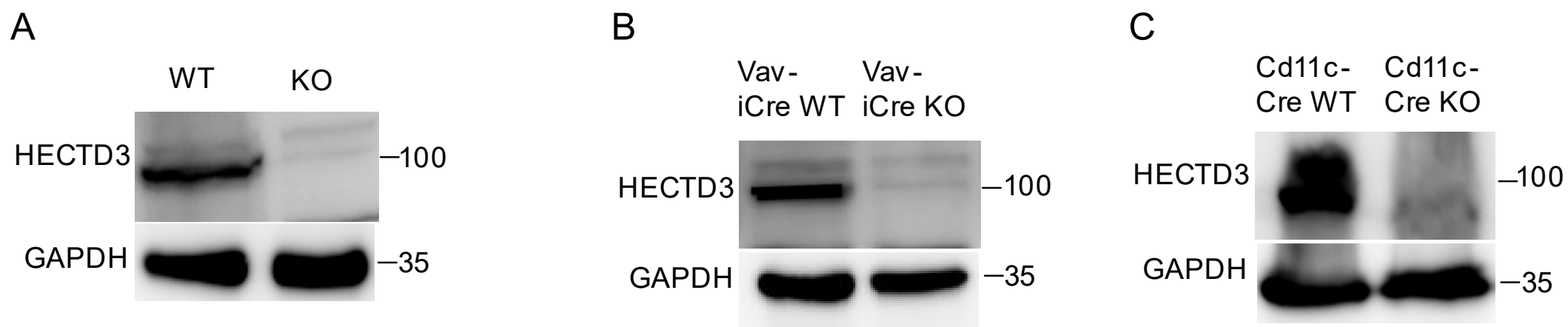
A
WT
KO
HECTD3
100
GAPDH
35
B
Vav-iCre WT
Vav-iCre KO
HECTD3
100
GAPDH
35
C
Cd11c-Cre WT
Cd11c-Cre KO
HECTD3
100
GAPDH
35

Figure S3. HECTD3 deletion in several murine experimental models. Western blot analysis of HECTD3 and GAPDH in: **(A)** spleen lysates of WT and Hectd3-KO (Germline); **(B)** spleen lysates of Vav-iCre WT and Hectd3$^{f/f}$Vav-icre mice; **(C)** bone marrow-derived cells from CD11c-Cre WT and Hectd3$^{f/f}$CD11c-Cre mice differentiated with 25 ng/mL GM-CSF.

Figure S4

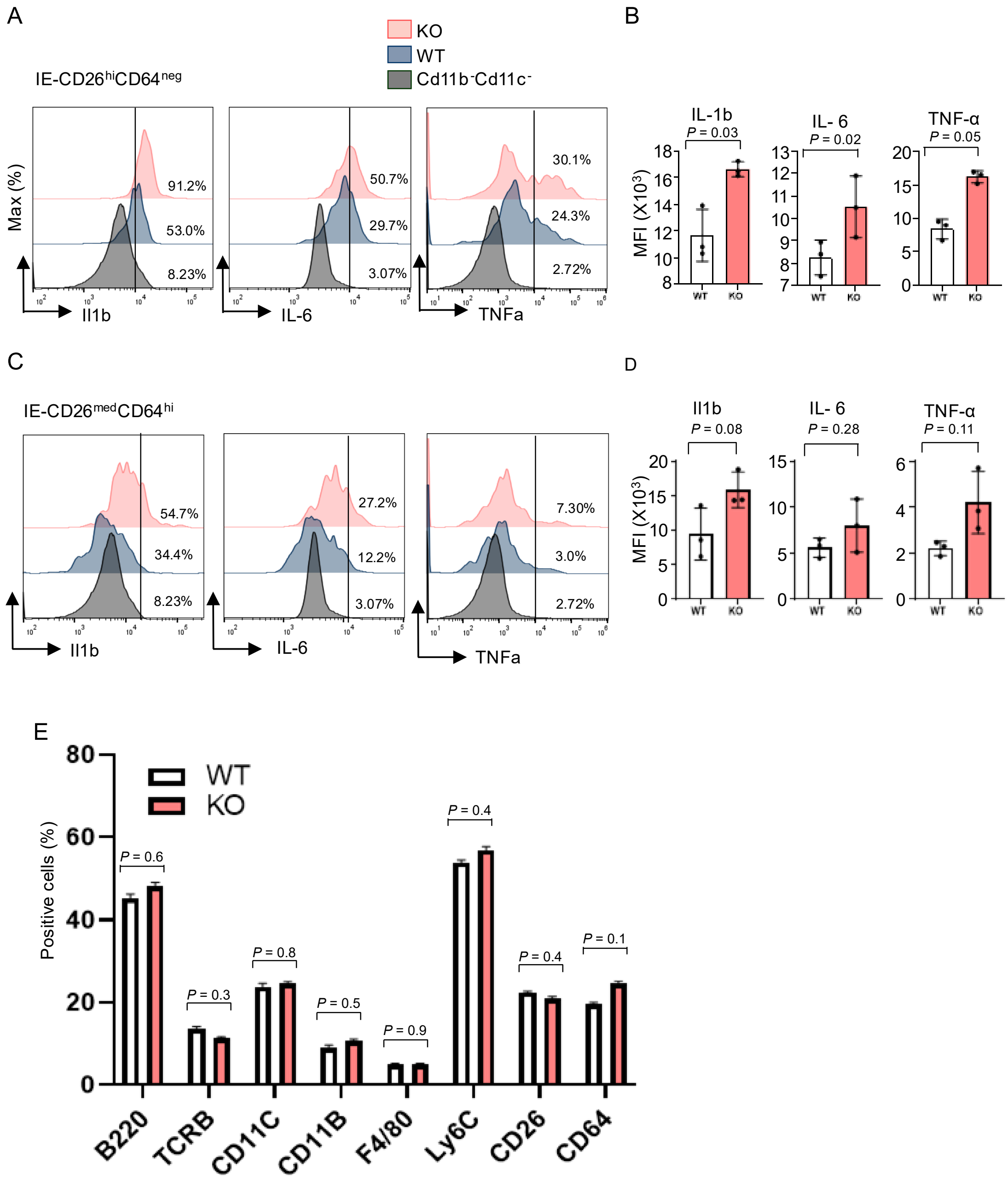
A
KO
WT
Cd11b-Cd11c-
IE-CD26hiCD64neg
Max (%)
91.2%
53.0%
8.23%
Il1b
50.7%
29.7%
3.07%
IL-6
30.1%
24.3%
2.72%
TNFa
B
IL-1b
P = 0.03
IL- 6
P = 0.02
TNF-α
P = 0.05
MFI (X10³)
WT
KO
C
IE-CD26medCD64hi
54.7%
34.4%
8.23%
Il1b
27.2%
12.2%
3.07%
IL-6
7.30%
3.0%
2.72%
TNFa
D
Il1b
P = 0.08
IL- 6
P = 0.28
TNF-α
P = 0.11
MFI (X10³)
E
WT
KO
Positive cells (%)
P = 0.6
P = 0.3
P = 0.8
P = 0.5
P = 0.9
P = 0.4
P = 0.4
P = 0.1
B220
TCRB
CD11C
CD11B
F4/80
Ly6C
CD26
CD64

Figure S4. Predominantly colonic intraepithelial (IE) $CD26^{hi}CD64^{neg}$ $Cd11b^{+}Cd11c^{+}$ population has elevated proinflammatory cytokines in Hectd3-KO mice with colitis. (A) Representative histograms of IL-1b, IL-6, TNF-α in colonic IE $CD26^{hi}CD64^{neg}$ $Cd11b^{+}Cd11c^{+}$ $MHCII^{+}$ cells from Hectd3-KO and WT mice following DSS treatment. $CD11c^{neg}CD11b^{neg}$ cells were used as negative control. (n= 3). **(B)** Mean Fluorescence Intensity (MFI) for IL-1b, IL-6, and TNF-α in colonic IE $CD26^{hi}CD64^{neg}Cd11b^{+}Cd11c^{+}MHCII^{+}$ cells from Hectd3-KO and WT mice following DSS treatment (n=3). **(C)** Representative histograms of IL-1b, IL-6, and TNF-α in colonic IE $CD26^{med}CD64^{hi}Cd11b^{+}Cd11c^{+}MHCII^{+}$ cells from Hectd3-KO and WT mice following DSS treatment. $CD11c^{-}CD11b^{-}$cells were used as negative control. **(D)** MFI for IL-1b, IL-6, and TNF-α in colonic IE $CD26^{med}CD64^{hi}Cd11b^{+}Cd11c^{+}MHCII^{+}$ cells from Hectd3-KO and WT mice following DSS treatment. (n=3). **(E)** Expression patterns of immune cell populations, including B cells (B220), T cells (TCRβ), dendritic cells (CD11c), monocytes/macrophages (CD11b), activated macrophages (F4/80), neutrophils (Ly6C), inflammatory cDC2s (inf-cDC2; CD26), and monocyte-derived DCs (moDCs; CD64). Percentage of marker-positive cells among the indicated immune cell populations. In B, D, and E, data are presented as mean ± SEM. p values were determined by Student's unpaired t-test.

Figure S5

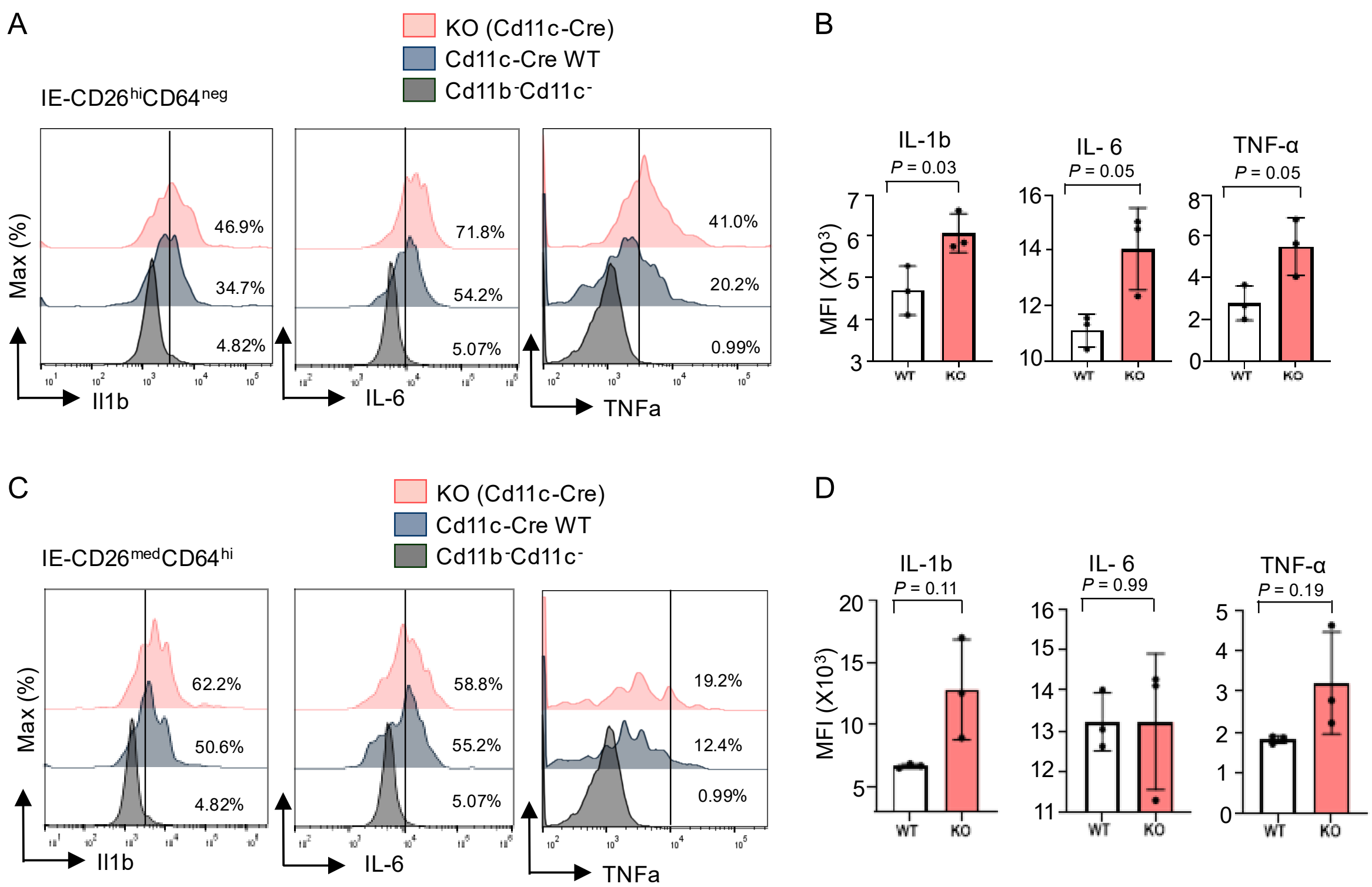

A
KO (Cd11c-Cre)
Cd11c-Cre WT
Cd11b-Cd11c-
IE-CD26hiCD64neg
Max (%)
46.9%
34.7%
4.82%
Il1b
71.8%
54.2%
5.07%
IL-6
41.0%
20.2%
0.99%
TNFa
B
IL-1b
P = 0.03
MFI (X10³)
WT
KO
IL- 6
P = 0.05
TNF-α
P = 0.05
C
KO (Cd11c-Cre)
Cd11c-Cre WT
Cd11b-Cd11c-
IE-CD26medCD64hi
Max (%)
62.2%
50.6%
4.82%
Il1b
58.8%
55.2%
5.07%
IL-6
19.2%
12.4%
0.99%
TNFa
D
IL-1b
P = 0.11
MFI (X10³)
IL- 6
P = 0.99
TNF-α
P = 0.19

Figure S5. Predominantly colonic IE $CD26^{hi}CD64^{-}$ $Cd11b^{+}Cd11c^{+}$ population has elevated proinflammatory cytokines in $Hectd3^{f/f}$CD11cCre mice. (A) Representative histograms of IL-1b, IL-6, and TNF-α in colonic IE $CD26^{hi}CD64^{neg}Cd11b^{+}Cd11c^{+}MHCII^{+}$ cells from $Hectd3^{f/f}$Cd11cCre mice and Cd11cCre WT mice following DSS treatment. $CD11c^{-}CD11b^{-}$ cells were used as negative control. (n= 3). **(B)** Mean Fluorescence Intensity (MFI) for IL-1b, IL-6, and TNF-α in colonic IE $CD26^{hi}CD64^{neg}Cd11b^{+}Cd11c^{+}MHCII^{+}$ cells from $Hectd3^{f/f}$Cd11cCre mice and Cd11cCre WT controls following DSS treatment. **(C)** Representative histograms of IL-1b, IL-6, TNF-αi n IE $CD26^{med}CD64^{hi}Cd11b^{+}Cd11c^{+}MHCII^{+}$ cells from $Hectd3^{f/f}$Cd11cCre mice and Cd11cCre WT controls following DSS treatment. $CD11c^{-}CD11b^{-}$ cells were used as negative control. (n= 3). **(D)** Mean Fluorescence Intensity (MFI) for IL-1b, IL-6, and TNF-α IE $CD26^{med}CD64^{hi}Cd11b^{+}Cd11c^{+}$ $MHCII^{+}$ cells from $Hectd3^{f/f}$Cd11cCre mice and WT mice following DSS treatment. (n=3). In B and D, Data are presented as mean ± SEM. *p* values by Student's unpaired t-test**.**

Figure S6

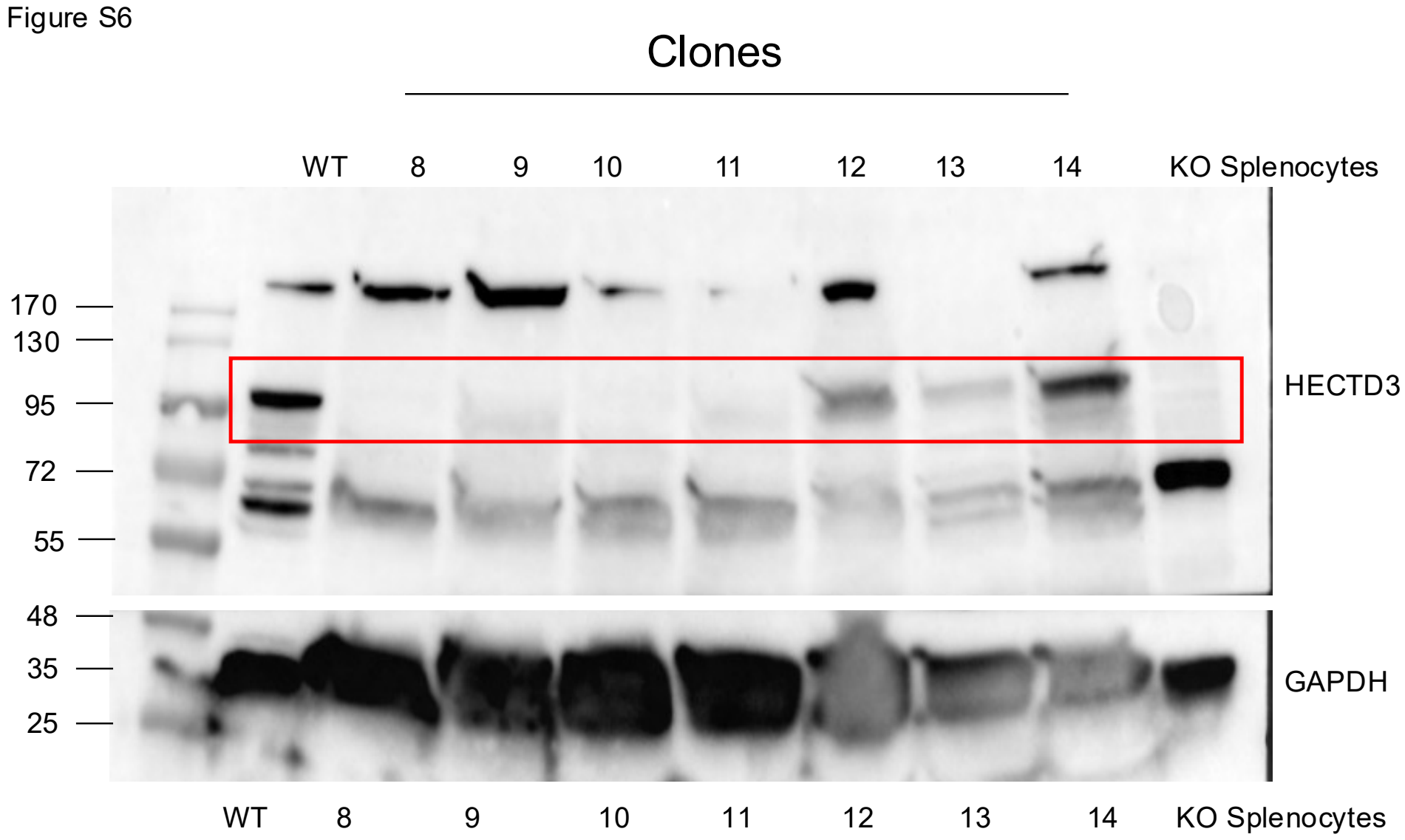
Clones
WT 8 9 10 11 12 13 14 KO Splenocytes
170
130
95
72
55
48
35
25
HECTD3
GAPDH
WT 8 9 10 11 12 13 14 KO Splenocytes

Figure S6. HECTD3-KO HEK293 cells generated by CRISPR-editing. Western blot analysis of several HECTD3-KO single cell clones generated by CRISPR editing, as described in the Material and Methods. Clone 11 was chosen for further studies.

Figure S7

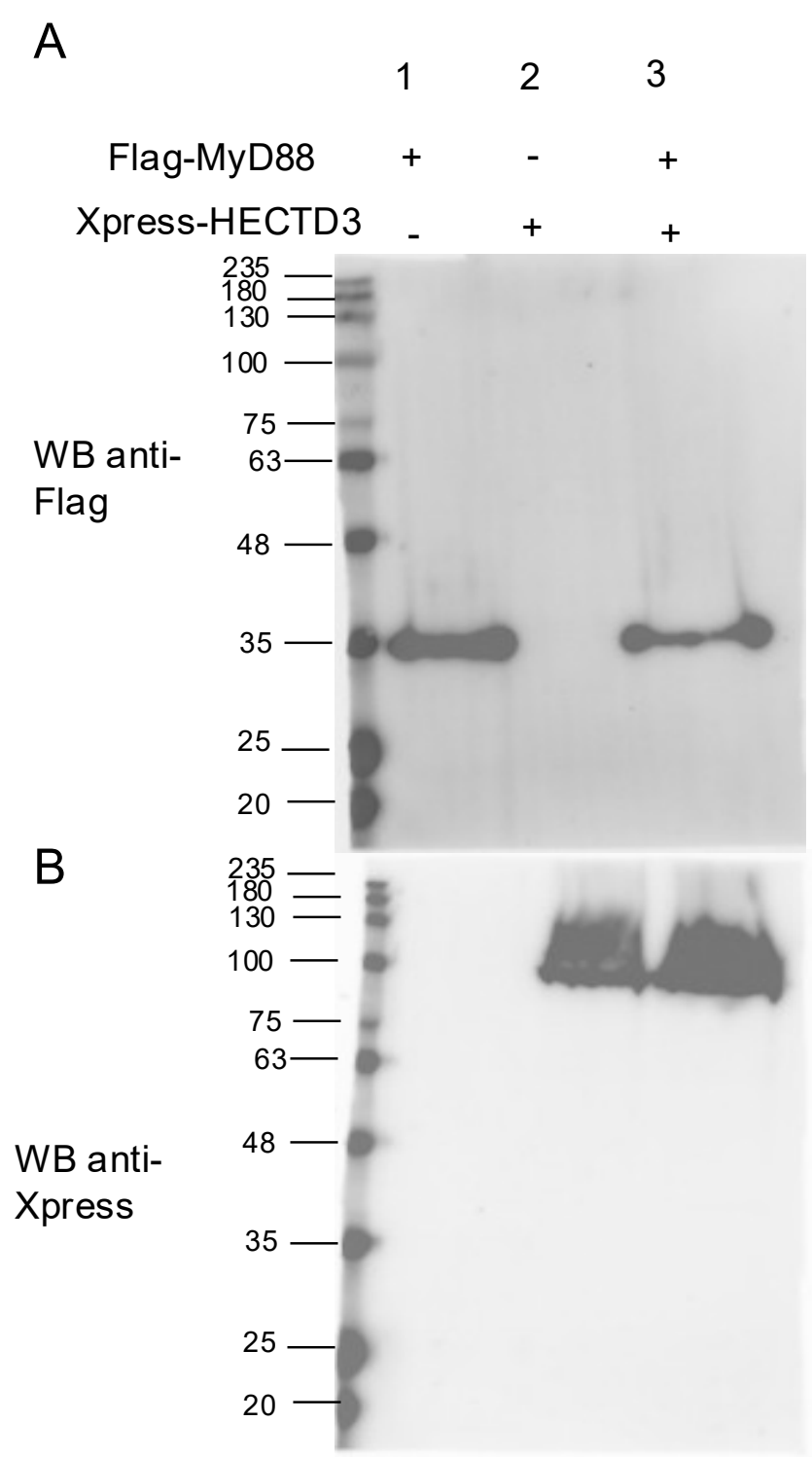
A
1
2
3
Flag-MyD88
+
-
+
Xpress-HECTD3
-
+
+
WB anti-Flag
235
180
130
100
75
63
48
35
25
20
B
235
180
130
100
75
63
48
35
25
20
WB anti-Xpress

Figure S7: Anti-Flag and anti-Xpress antibody specificity and lack of cross-reactivity. (A-B) HECTD3-KO HEK293 cells were transfected with Flag-MyD88 only (lane 1), Xpress- HECTD3 only (lane 2), or both Flag-MyD88 and Xpress- HECTD3 (lane 3). **(A)** Immunoblot with anti-Flag antibody showing that it only recognizes the ~35 kDa Flag-MyD88 band (lanes 1 and 3) and does not detect the Xpress- HECTD3 (~100 kDa) (lanes 2 and 3). **(B)** Immunoblot with anti-Xpress antibody showing that it only detects the ~100 kDa band corresponding to Xpress- HECTD3 (lanes 2 and 3) and does not detect the ~35 kDa Flag-MyD88 band (lanes 1 and 3).

Figure S8

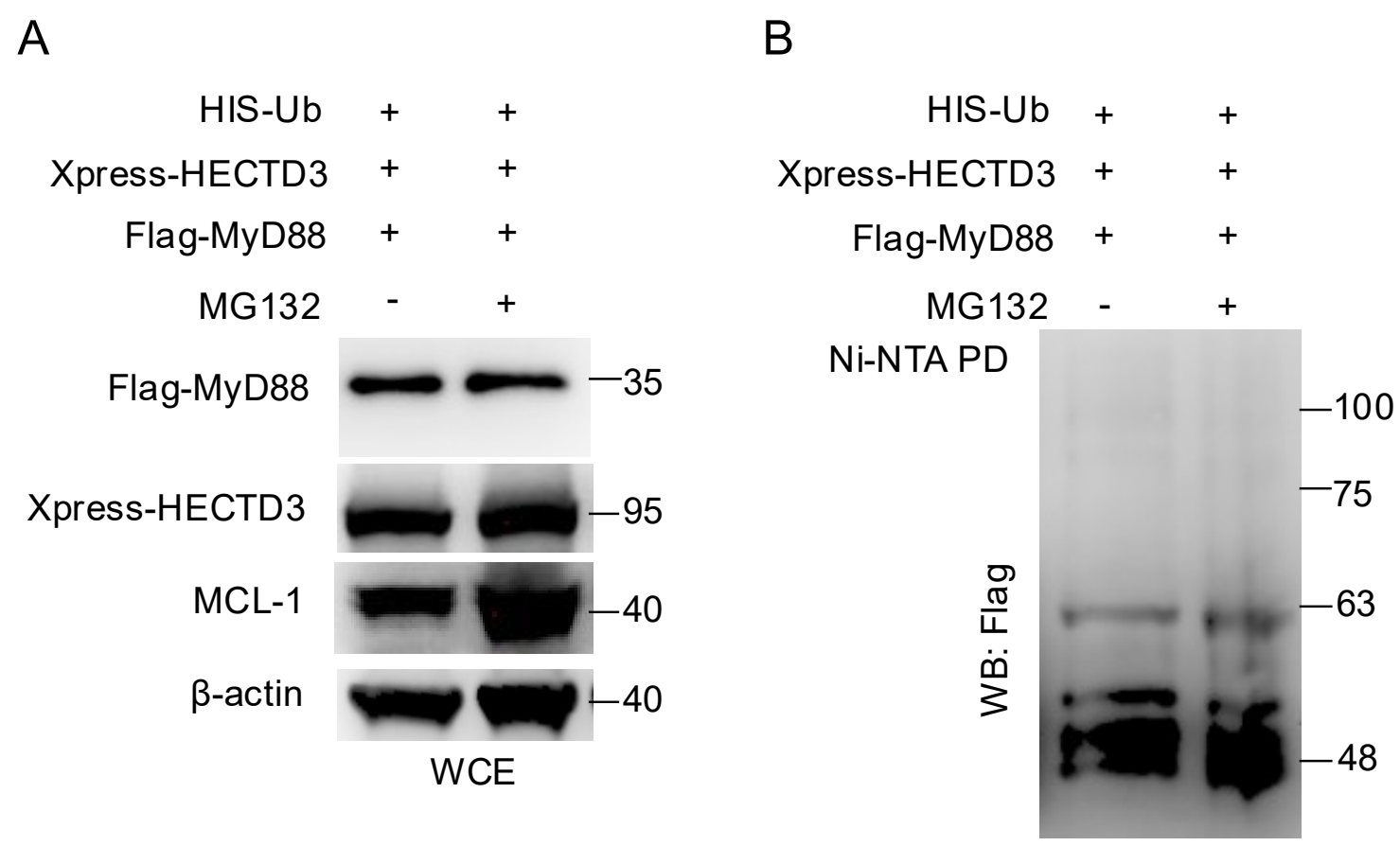
A
HIS-Ub + +
Xpress-HECTD3 + +
Flag-MyD88 + +
MG132 - +
Flag-MyD88
35
Xpress-HECTD3
95
MCL-1
40
β-actin
40
WCE
B
HIS-Ub + +
Xpress-HECTD3 + +
Flag-MyD88 + +
MG132 - +
Ni-NTA PD
WB: Flag
100
75
63
48

Figure S8. HECTD3 does not target MyD88 for proteasomal degradation. **(A)** Anti-Xpress, anti-Flag, anti-MCL1 and anti-β-actin immunoblots of whole cell extracts (WCE) from HECTD3-KO HEK293T cells co-transfected with Flag-MyD88, Xpress- HECTD3 and HIS-Ub, and treated or not with 20μM MG132 for 4 hours prior to protein extraction. **(B)** Anti-Flag immunoblot of Ni-NTA PD protein extracts from HECTD3-KO HEK293T cells co-transfected with Flag-MyD88, Xpress- HECTD3 and HIS-Ub, and treated or not with 20μM MG132 for 4 hours prior to protein extraction.

Supplementary Table 1

Primers used for Site directed mutagenesis

Name of primer	Sequence
MyD88 Δaa54-109 forward	5’-GAGGAGGACTGCCAGAAATACTTAG-3’
MyD88 Δaa54-109 reverse	5’-CTCCTCCGCCAGCAAGGT-3’
MyD88 Δaa110-155 forward	5’-CAAACGCCGGAACTTTTC-3’
MyD88 Δaa110-155 reverse	5’-GATGCGCGACTTCAGCTC-3’
MyD88 Δaa159-296 forward	5’- TGAAGATGACCCTGGGAACC -3’
MyD88 Δaa159-296 reverse	5’- CGGCGTTTGTCCTAGGGG -3’
MyD88 K103R forward	5’-GAGTCCGAGAGGCCTTTACAG-3’
MyD88 K103R reverse	5’-CTGGTTCTGCTGCTTACC-3’
MyD88 K119R forward	5’-GATATACTGAGGGAGCTGAAGTC-3’
MyD88 K119R reverse	5’-CTCACGGTCTAACAAGGC-3’
MyD88 K127R forward	5’-TACTTAGGTAGGCAGCAGAAC-3’
MyD88 K127R reverse	5’-TTTCTGGCAGTCCTCCTC-3’
Hectd3 C823A forward	5’-GTCTTCTACCGCATCCAGCACGCTCTTCCTCC-3’
Hectd3 C823A reverse	5’-TCAGGCAGGGCGTCTGTG-3’

Supplementary Table 2

Antibodies used in this study

Antibody	Antibody dilution	SOURCE	IDENTIFIER
Anti-mouse CD11b BV480, M1/70	1:100	BD Biosciences	cat: 566117
Anti-mouse CD11c BV650, N418	1:50	BD Biosciences	cat: 117339
anti-mouse CD45.2 APC,104	1:200	Biolegend	cat: 109814
Anti-mouse F4/80 BUV 615, T45-2342	1:50	BD Biosciences	cat: 751454
anti-mouse IL-6 perCp-eFluor 710, MP5-20F3	1:75	eBioscience	46-7061-82
anti mouse IL-1β FITC, NJTEN3	1:50	eBioscience	11-7114-80
Anti-mouse MHCII BV 421, M5/114.15.2	1:800	Biolegend	107631
Anti-mouse TCRb BUV805, H57-597	1:400	BD Biosciences	748405
Anti-mouse CD86 BUV563, GL1	1:100	BD Biosciences	741259
Anti-mouse CD64 PE/Dazzle 594, X54-5/7.1	1:50	Biolegend	139319
B220, Anti-mouse BV750, RA3-6B2	1:100	Biolegend	103261
Anti-mouse CD26 PE/Cy7, G194-112	1:50	Biolegend	137810
anti-mouse TNF-α BV605, MP6-XT22	1:200	eBioscience	Cat# 506329
Phospho-NF-κB p65 (Ser536) (93H1)	1:1000	Cell Signaling Technology, MA, USA	Cat# 3033S
NF-κB p65	1:1000	Cell Signaling Technology, MA, USA	Cat# 8242S
Anti-mouse LY6C APC/Cy7 HK1.4	1:1000	Biolegend	Cat# 128026
Anti-Flag M2 antibody	1:1000	Sigma, Millipore-Sigma, MO, USA	Cat#F1804
Anti-Xpress antibody	1:500	Invitrogen, Thermo Fisher Scientific, PA, USA	Cat# 460528
GAPDH (14C10)	1:3000	Cell Signaling Technology, MA, USA	Cat# 2118L
Hectd3	1:2000	Bethyl Laboratories, TX, USA	Cat# A304-924A
Anti HIS	1:1000	Santa Cruz	Cat# 8036

Anti MyD88	1:1000	Cell Signaling Technology, MA, USA	Cat#4283
MCL-1	1:2000	Cell Signaling Technology, MA, USA	Cat#4572
IRAK4	1:1000	Cell Signaling Technology, MA, USA	Cat#4363
Phospho-IRAK4 (Thr345/Ser346)	1:1000	Cell Signaling Technology, MA, USA	Cat#11927
Lamin A/C	1:2000	Cell Signaling Technology, MA, USA	Cat#4777S
Peroxidase AffiniPure Goat Anti-Mouse IgG (H + L)	1:4000	Jackson ImmunoResearch, PA, USA	Cat#115-035-003
Peroxidase AffiniPure Goat Anti-Rabbit IgG (H + L)	1:4000	Jackson ImmunoResearch, PA, USA	Cat#111-035-003
Peroxidase AffiniPure Goat Anti-Rat IgG (H + L)	1:4000	Jackson ImmunoResearch, PA, USA	Cat#112-035-003
