# Supplementary material for: The E3 ubiquitin ligase HECTD3 controls immune colonic inflammation by restricting MyD88 activation and signaling
Source: JCI Insight. 2026 Sep 24;11(20):e185437. doi: 10.1172/jci.insight.185437 (PMC13726568; doi:10.1172/jci.insight.185437)

Original image with molecular weight marker

Full unedited gel

Figure 4

A

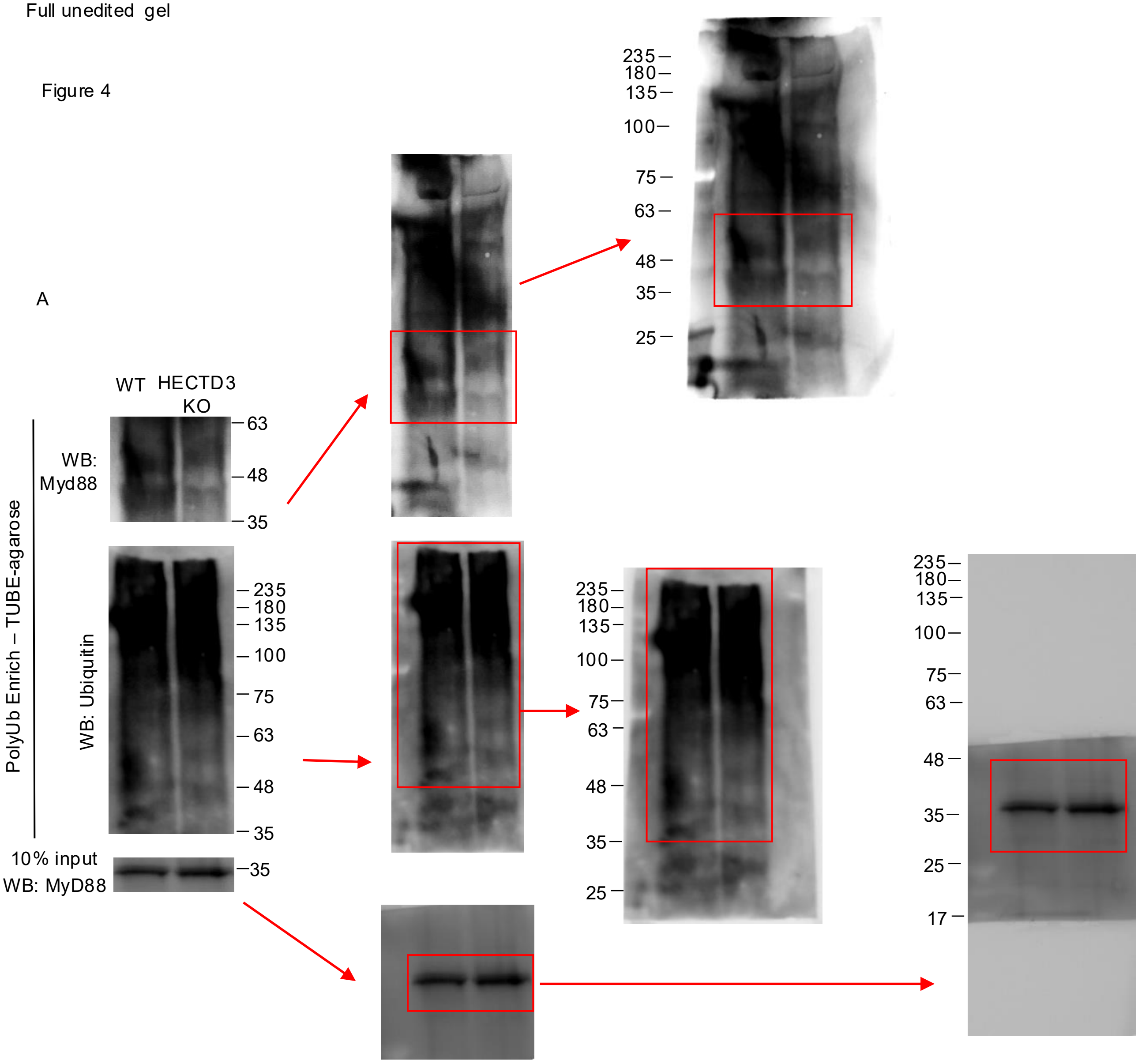

Original image with molecular weight marker

Full unedited gel

Figure 4

B

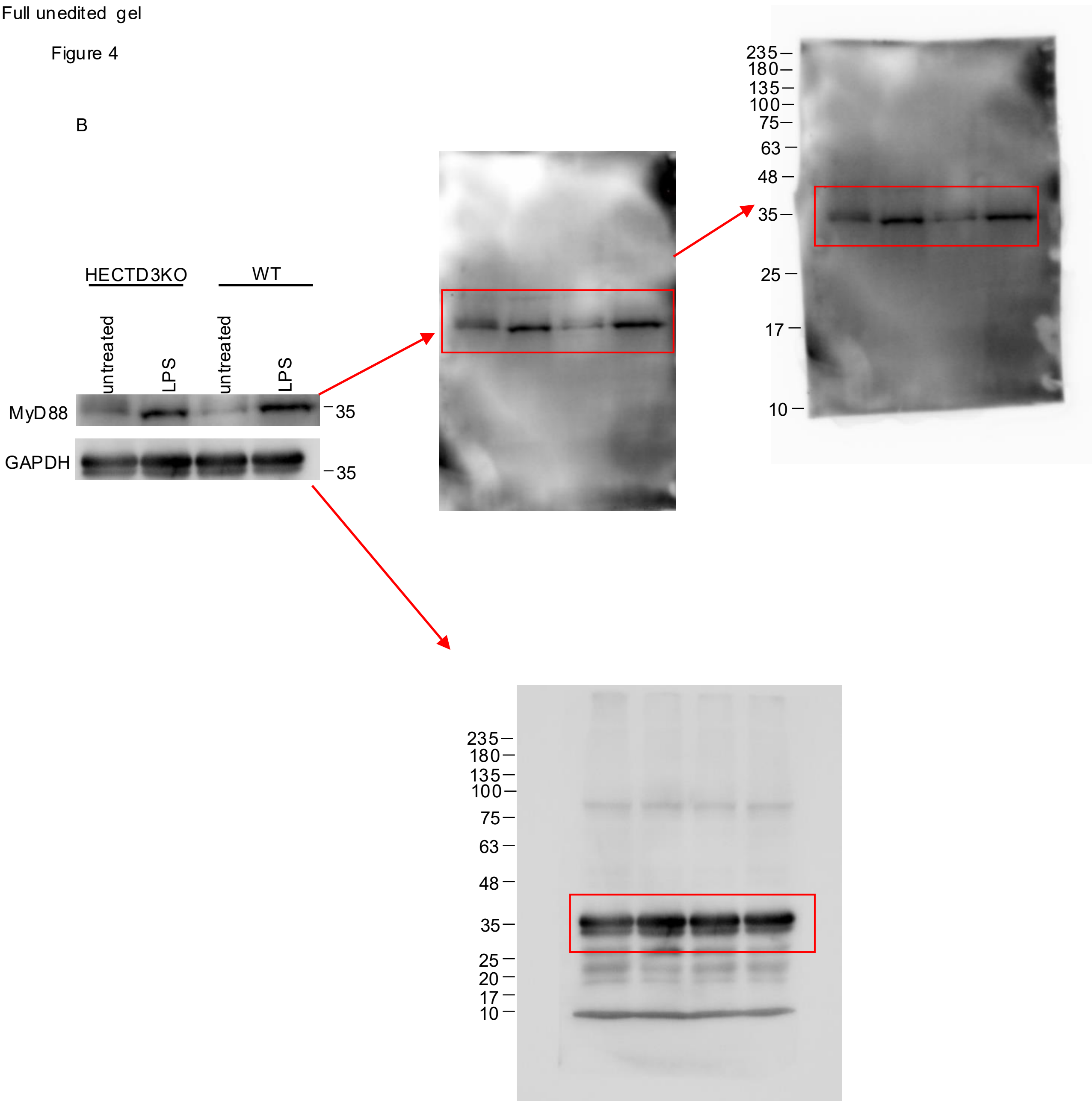

Original image with molecular weight marker

Full unedited gel

Figure 4

C

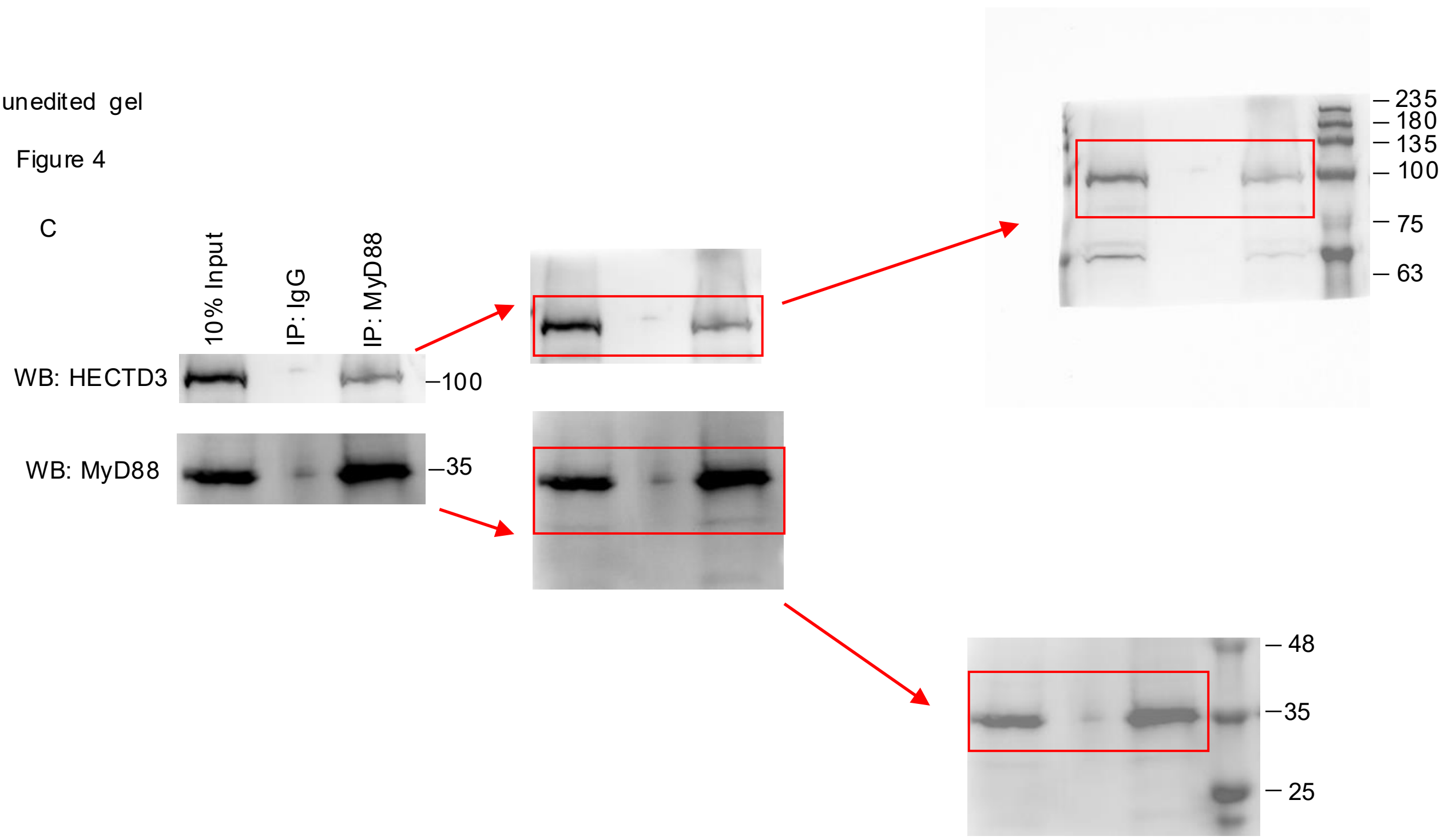

Original image with molecular weight marker

Full unedited gel

Figure 4

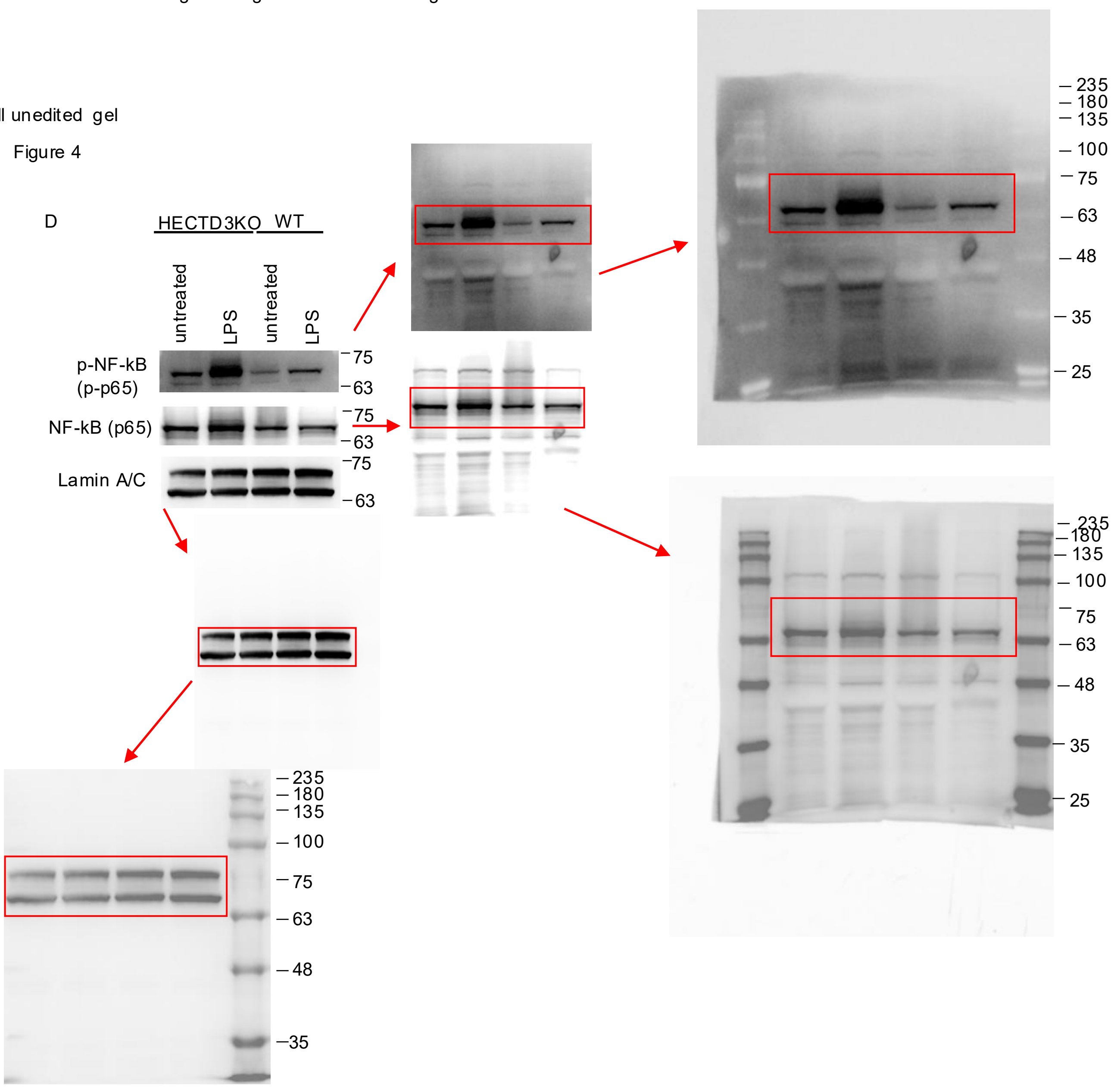

Original image with molecular weight marker

Full unedited gel

Figure 5

A

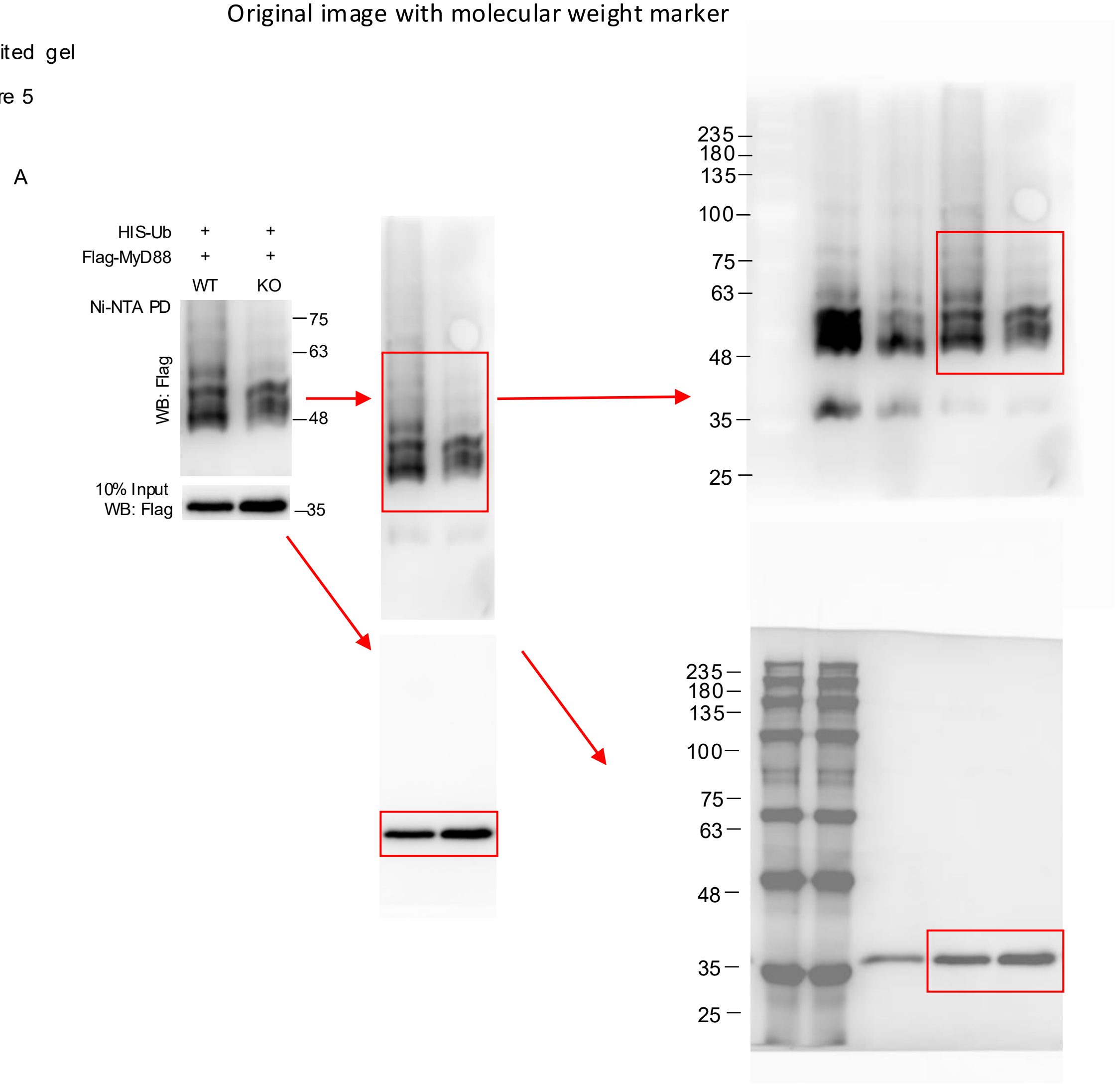

Original image with molecular weight marker

Full unedited gel

Figure 5

B

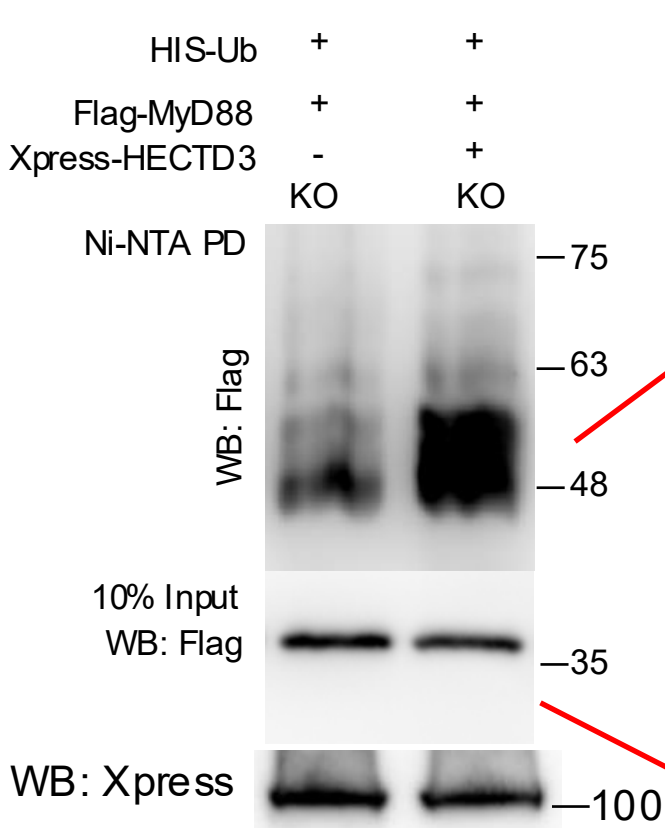


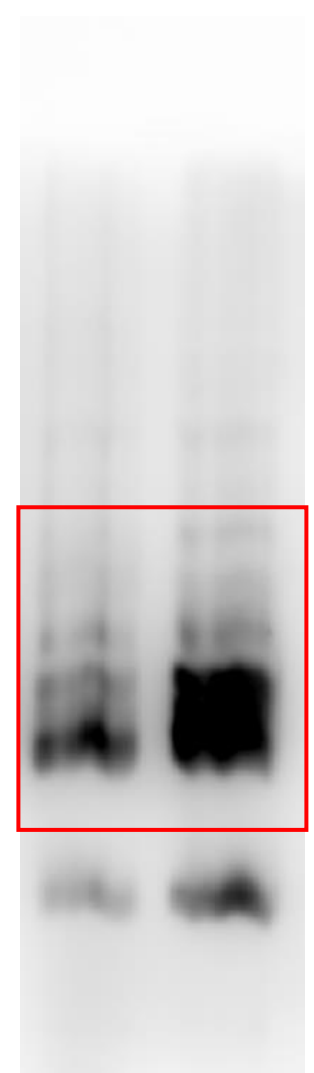

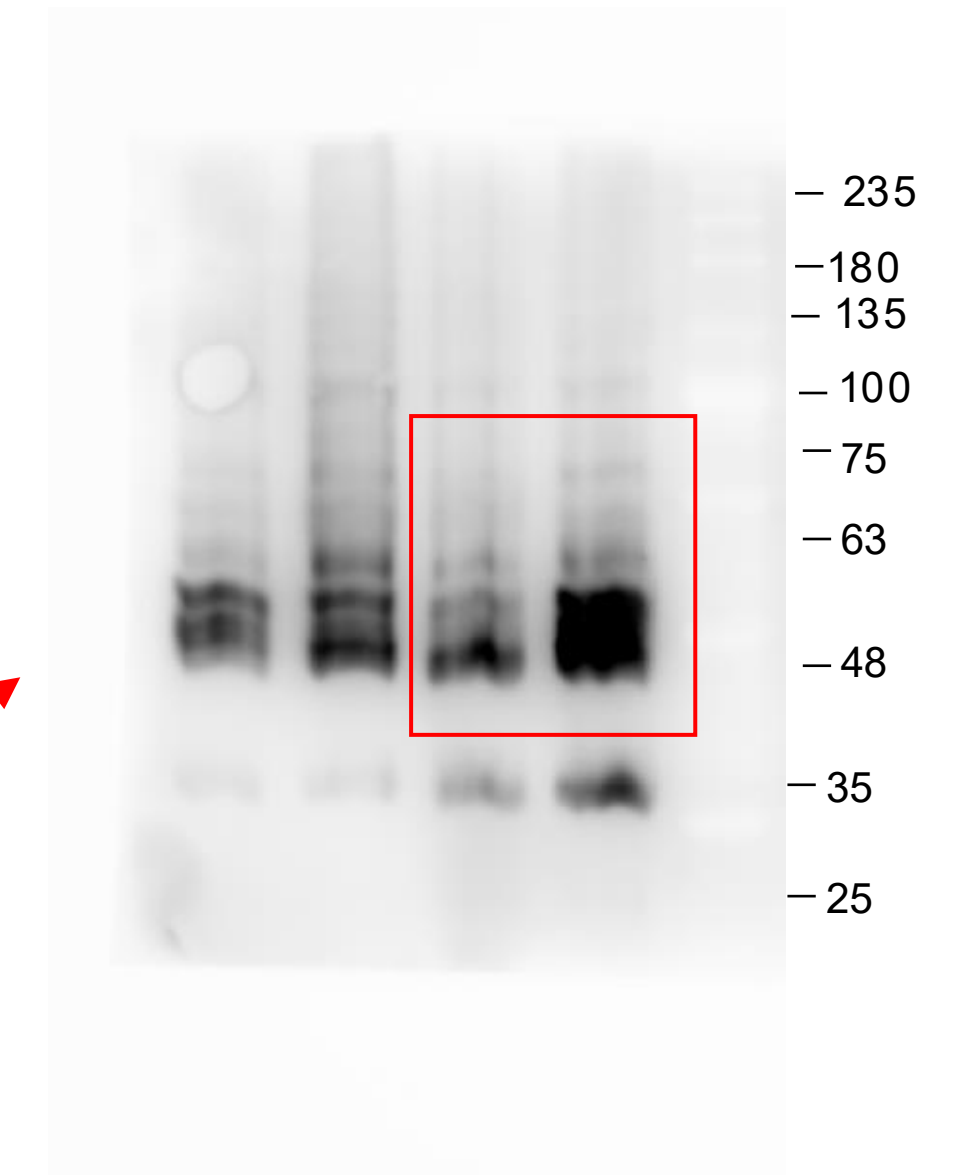


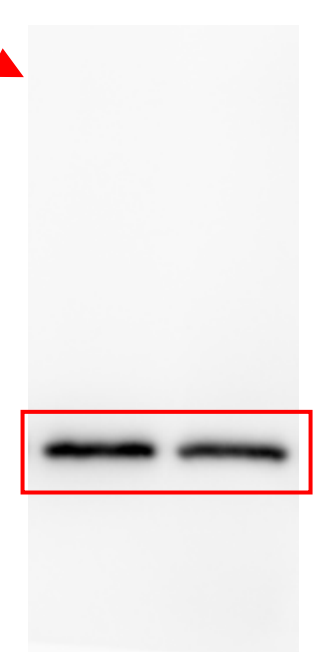

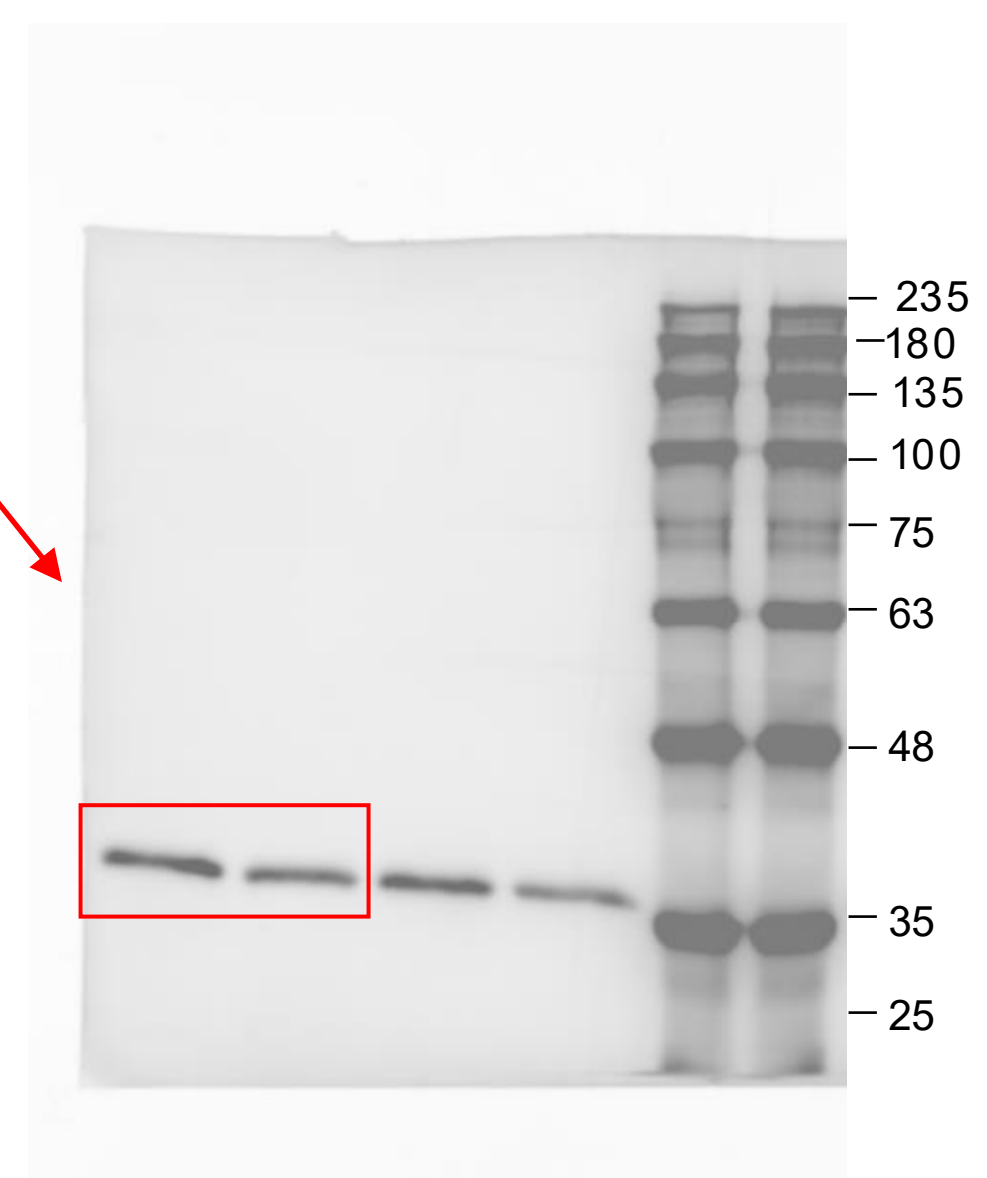


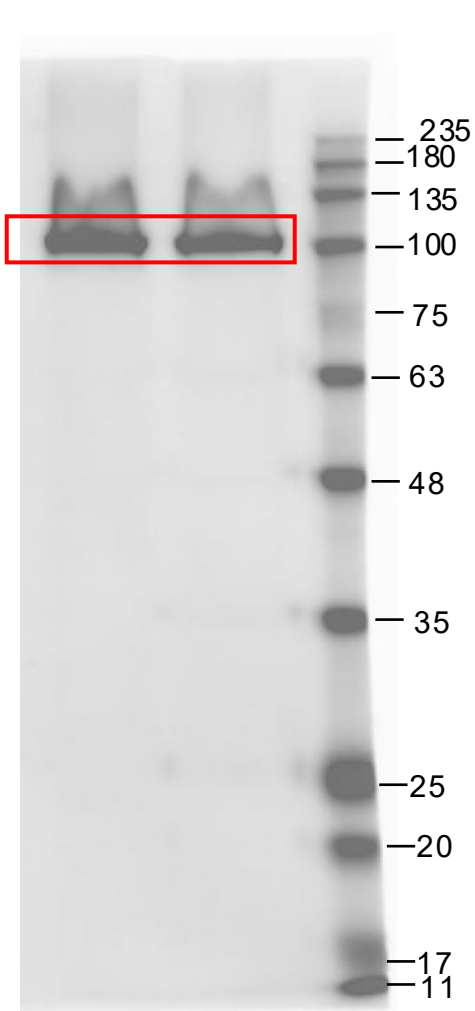

Original image with molecular weight marker

Full unedited gel

Figure 5

C

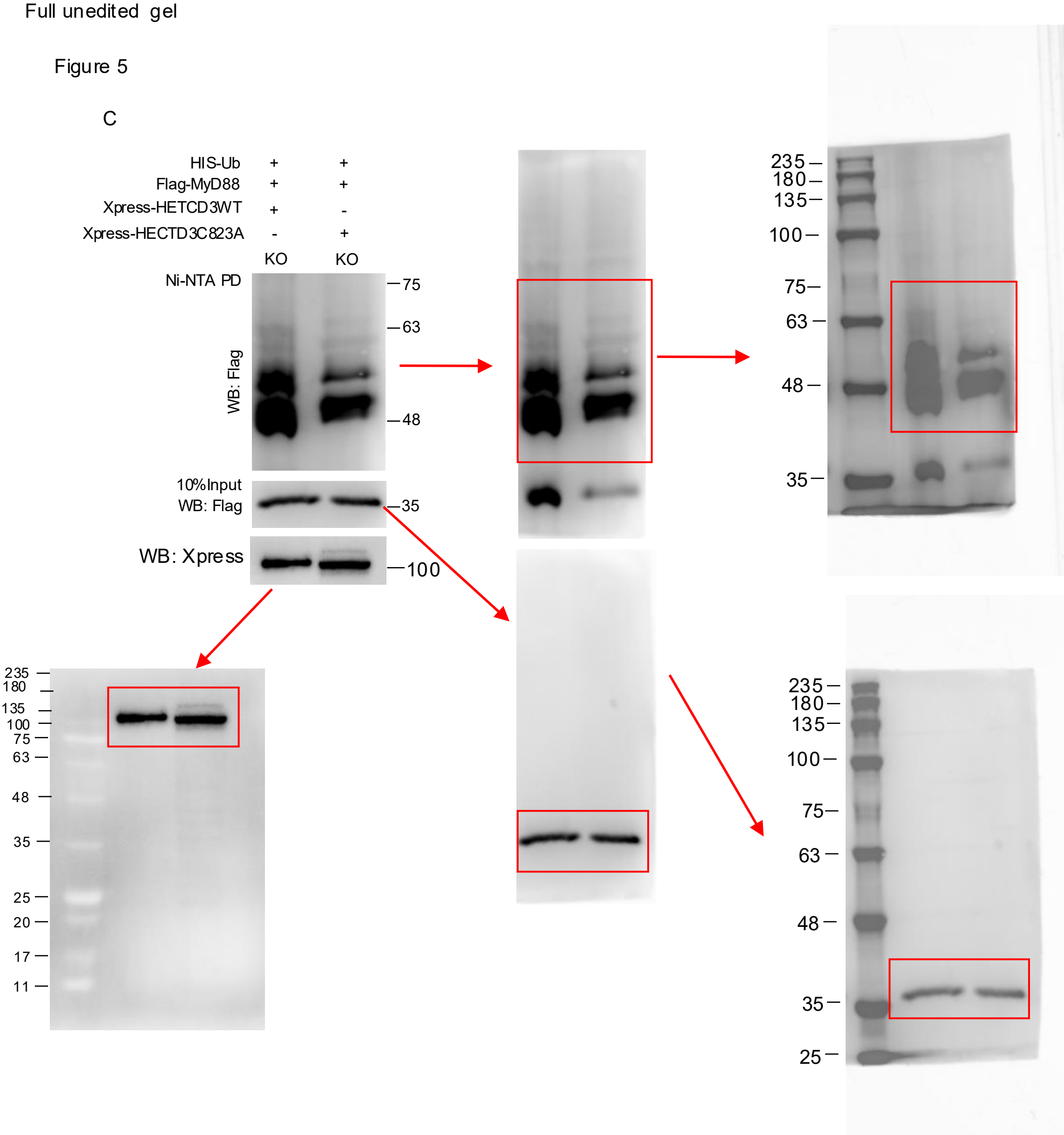

Original image with molecular weight marker

Full unedited gel

Figure 5

D

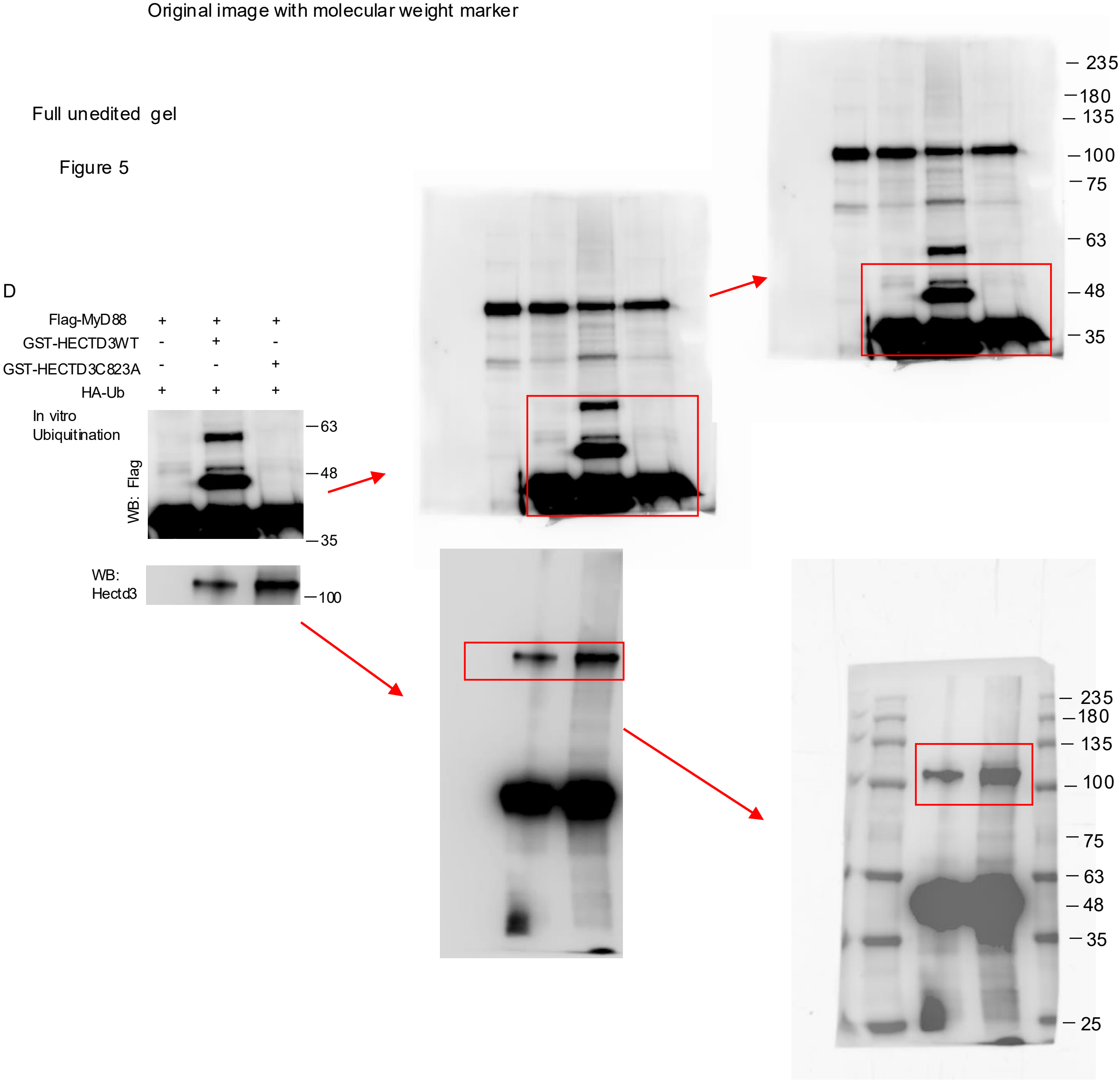

Original image with molecular weight marker

Full unedited gel

Figure 5

E

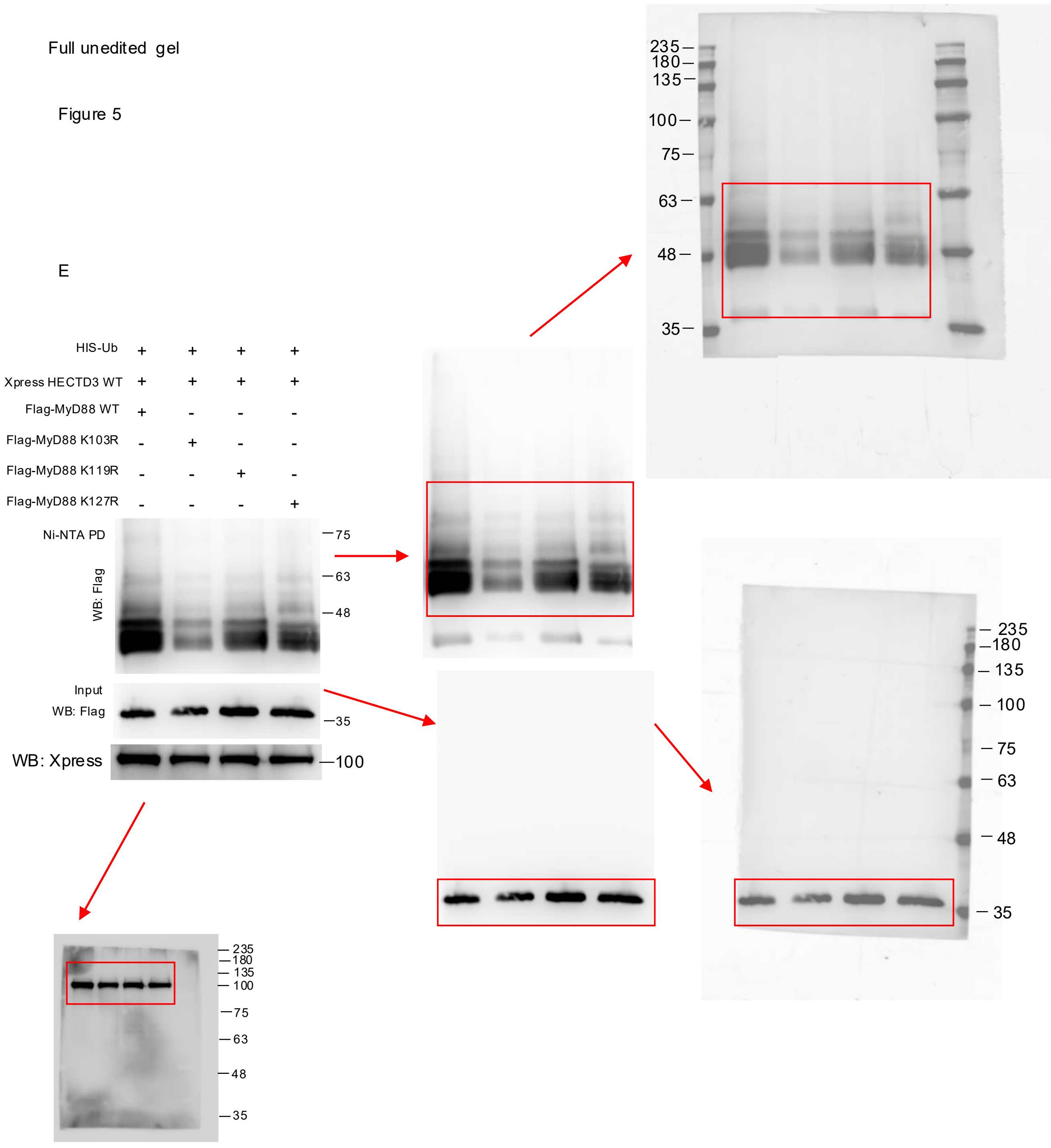

Original image with molecular weight marker

Full unedited gel

Figure 6

A

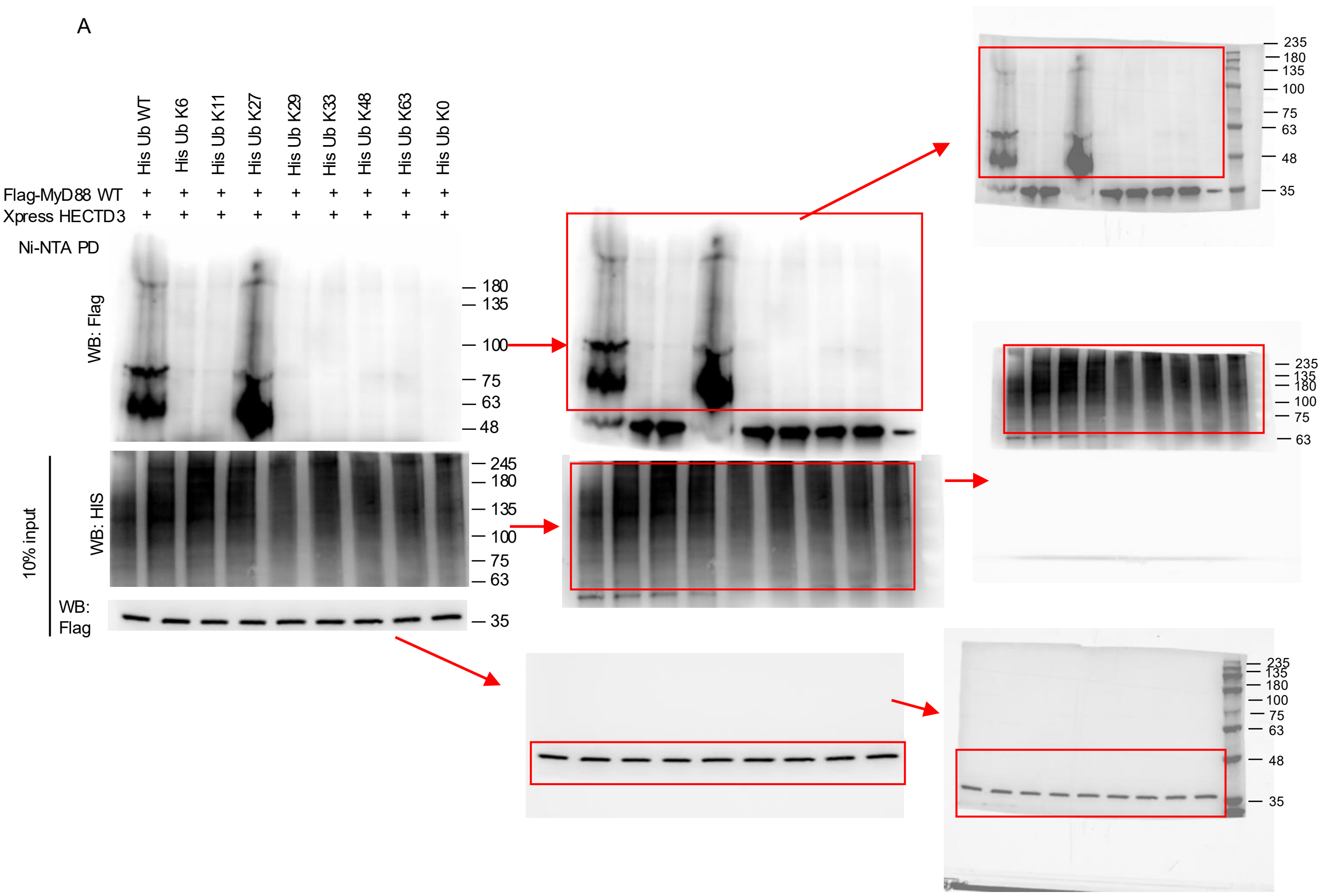

Full unedited gel

Original image with molecular weight marker

Figure 6

B

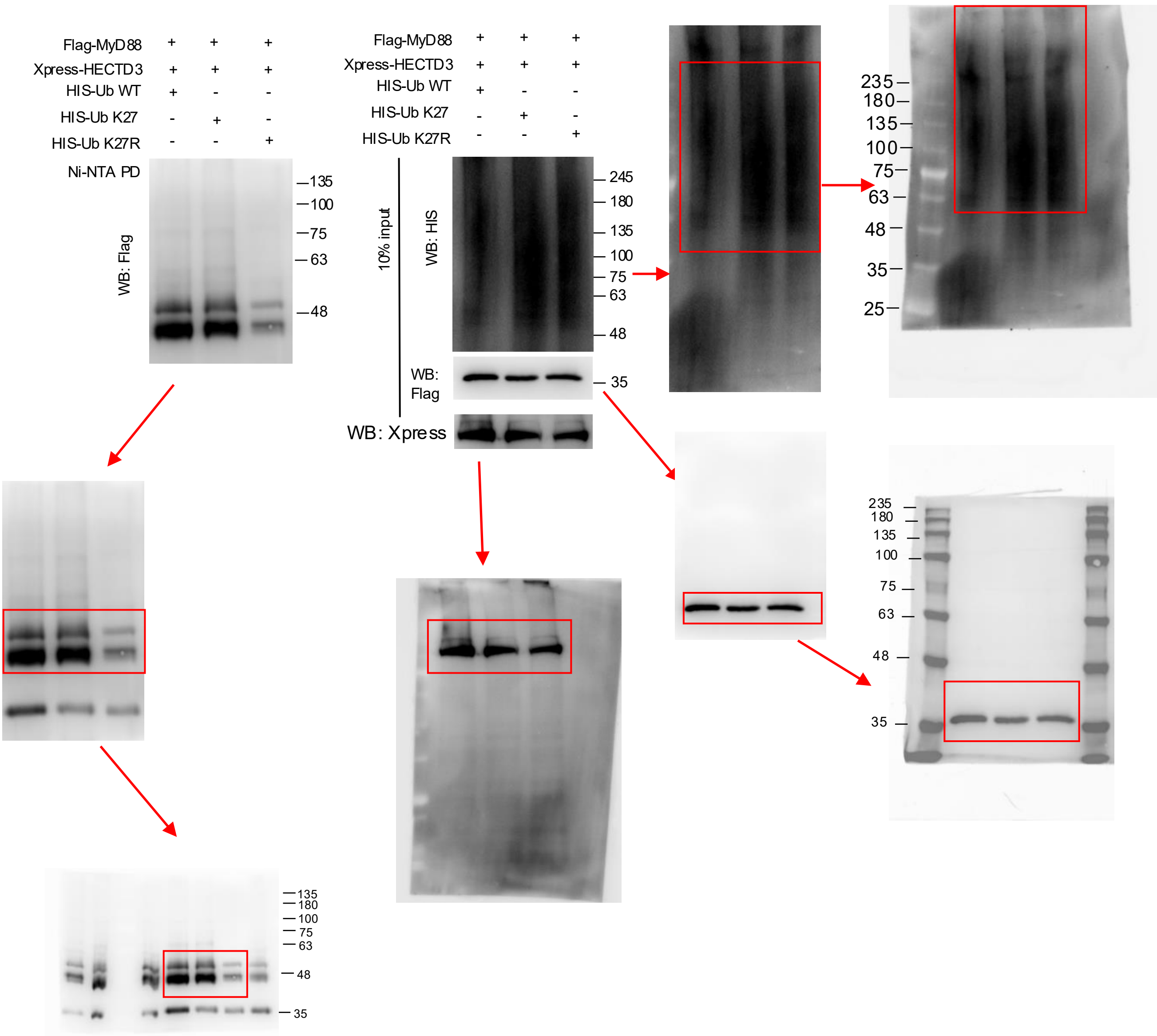

Original image with molecular weight marker

Full unedited gel

Figure 7

A

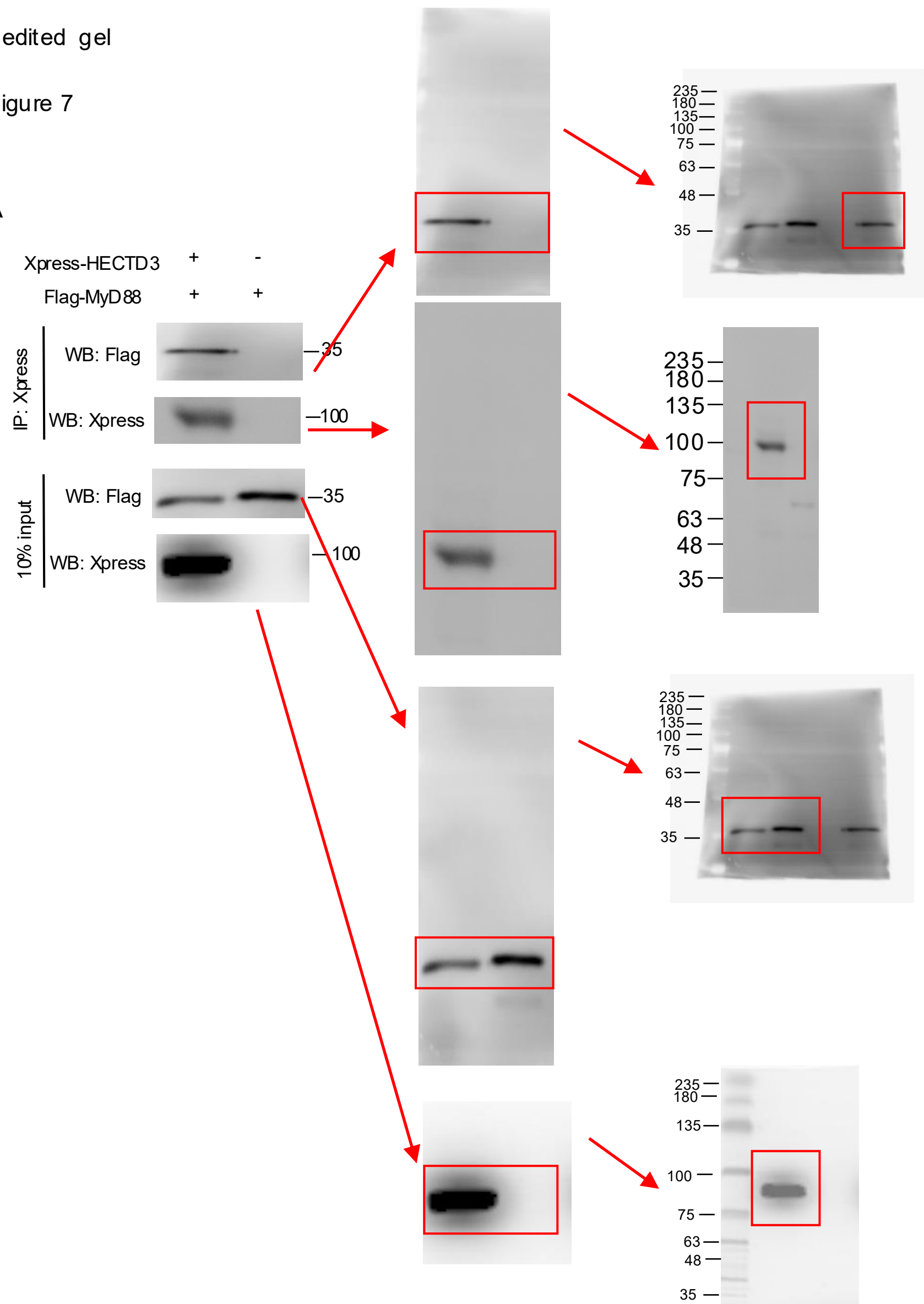

Original image with molecular weight marker

Full unedited gel

Figure 7

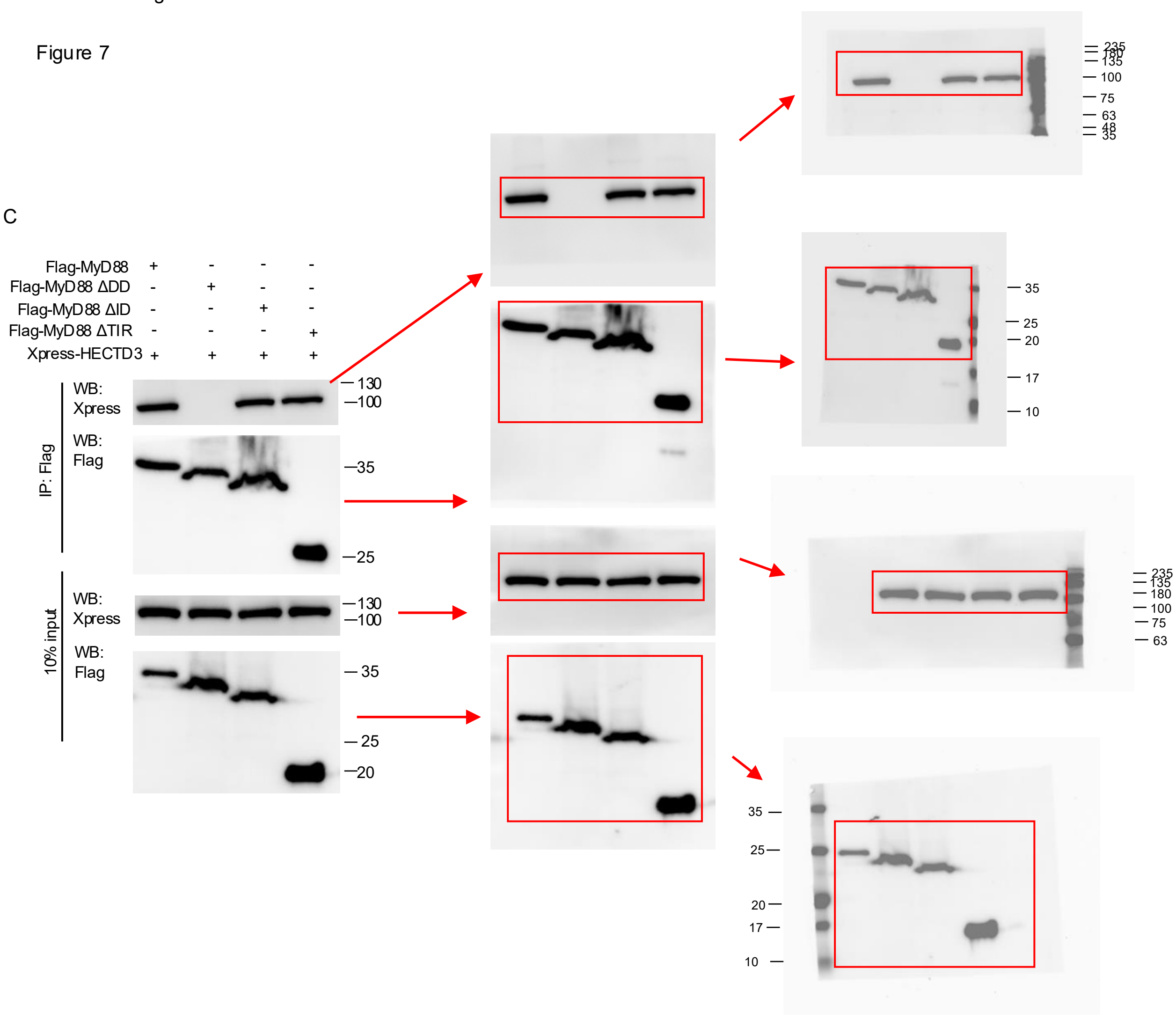

Original image with molecular weight marker

Full unedited gel

Figure 7

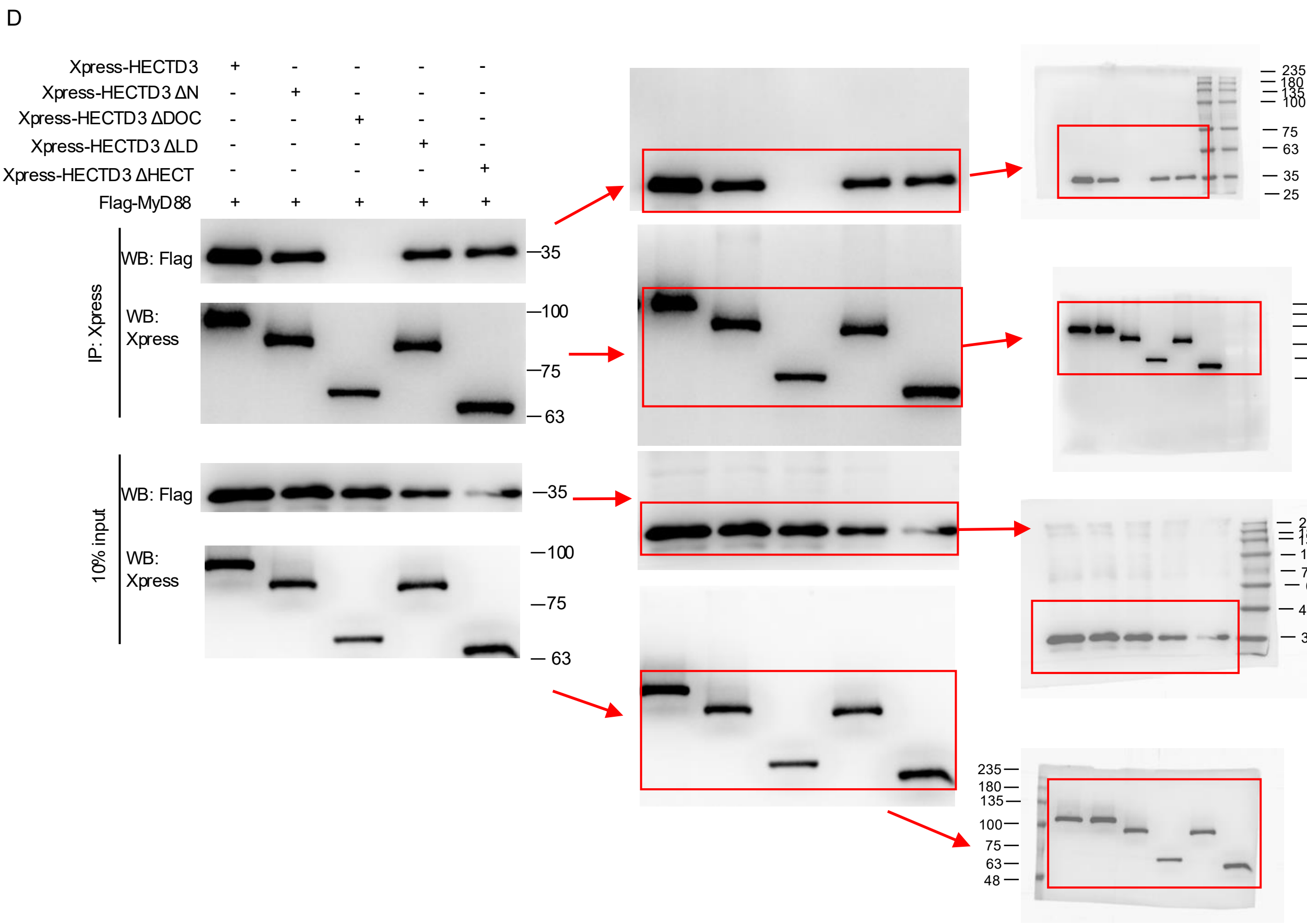

Original image with molecular weight marker

Full unedited gel

Figure 8

B

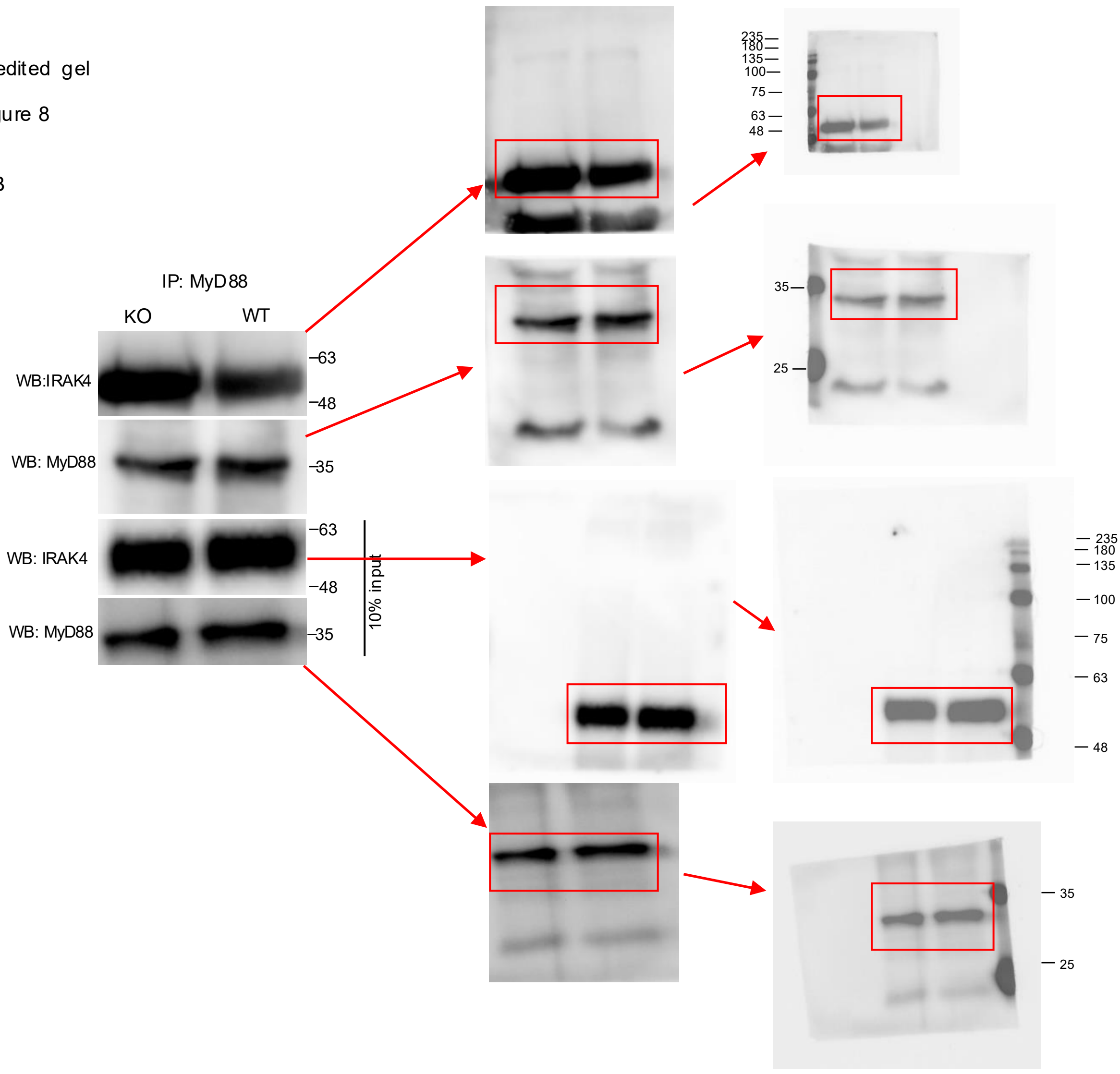

Original image with molecular weight marker

Full unedited gel

Figure 8

C

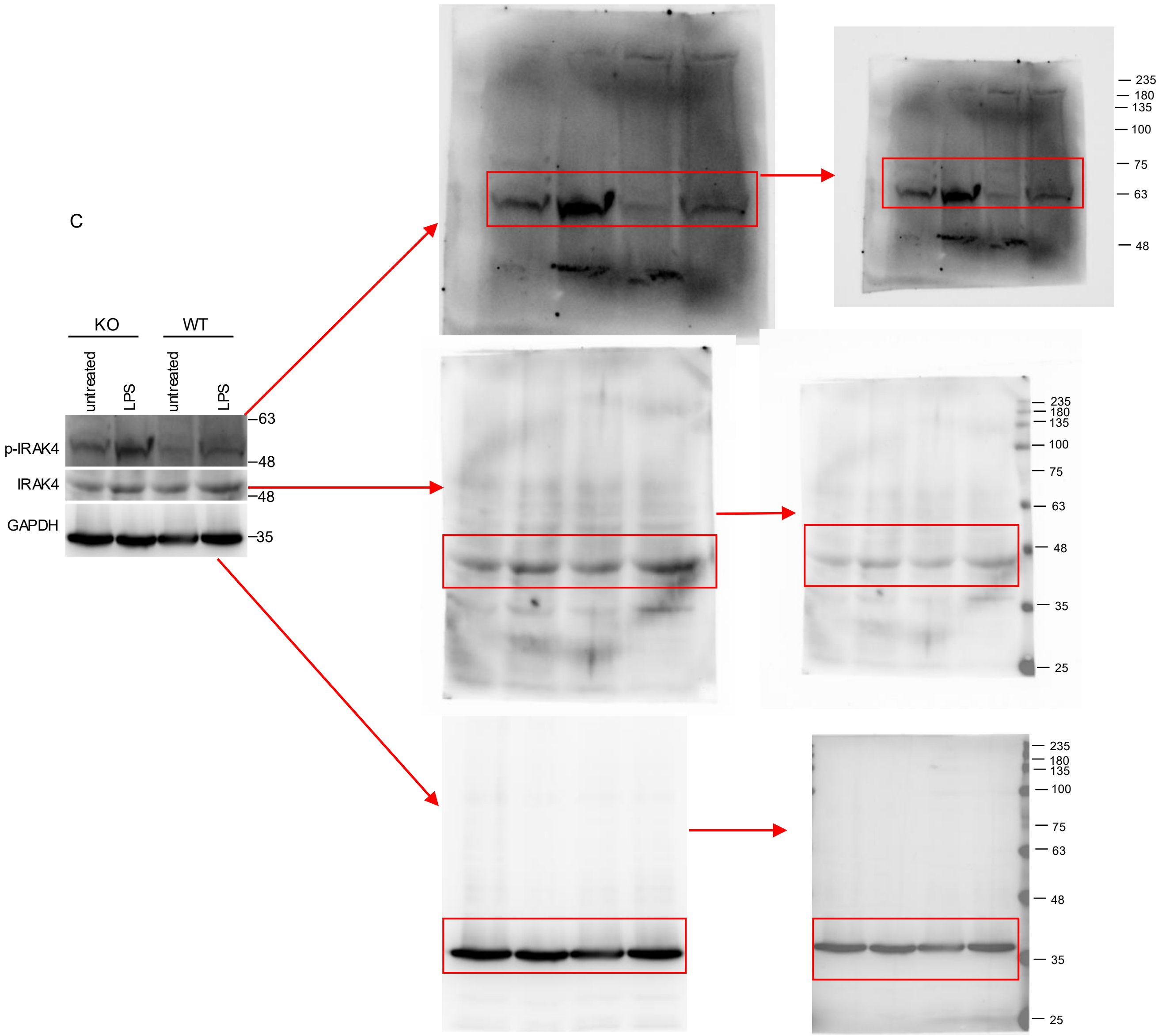

Original image with molecular weight marker

Full unedited gel

Figure 8

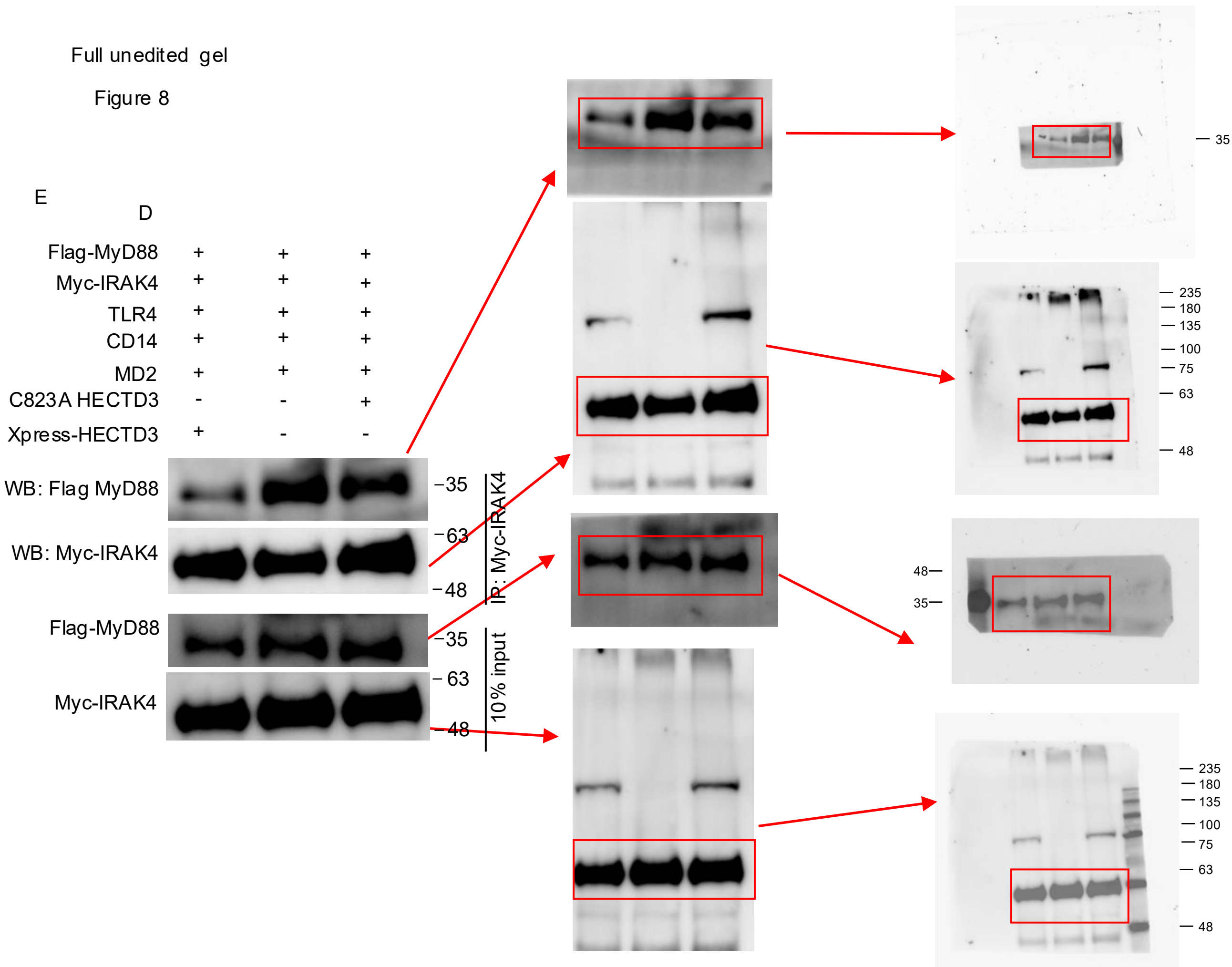

Original image with molecular weight marker

Figure S3

Full unedited gel

A

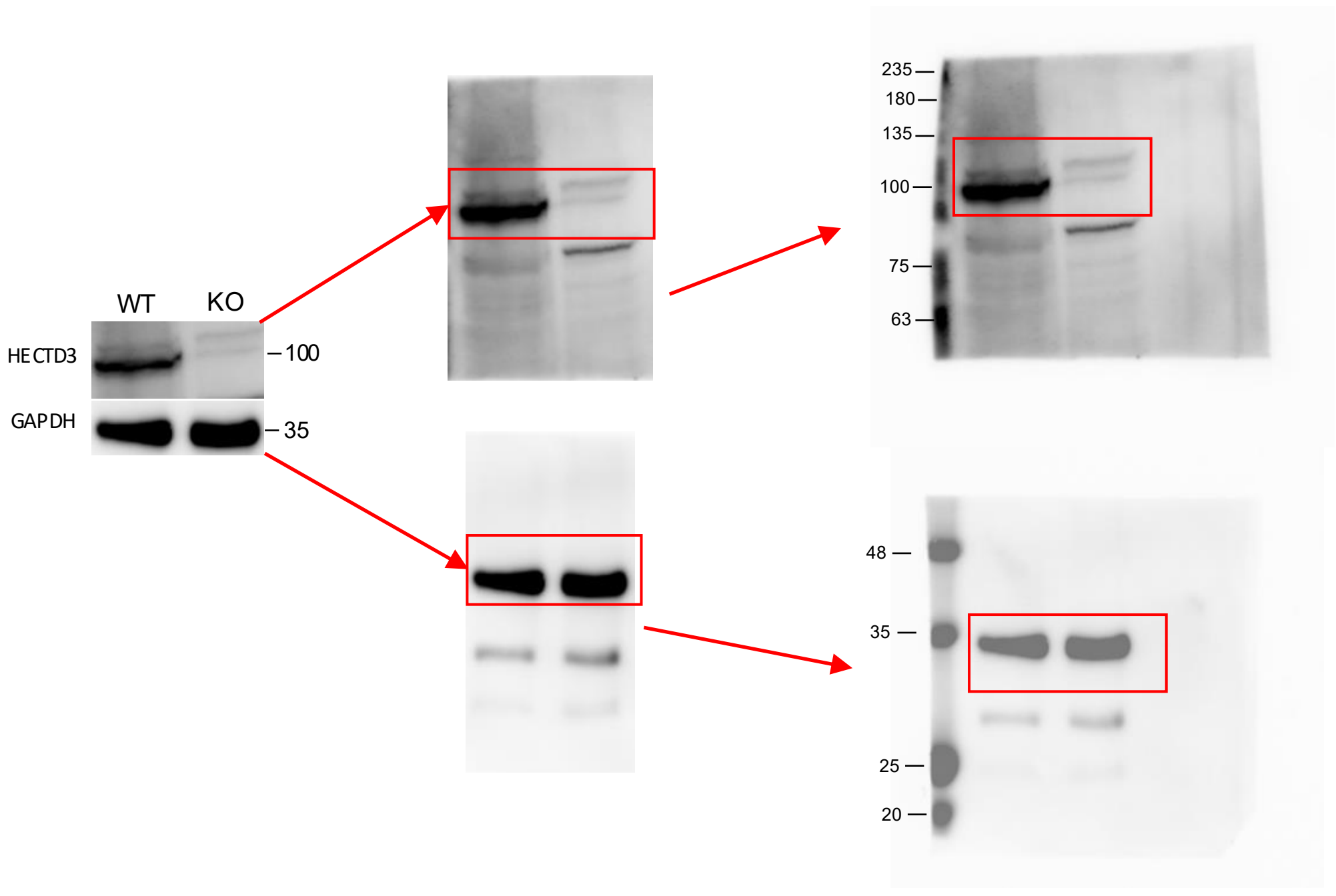

Full unedited gel

Original image with molecular weight marker

Figure S3

B

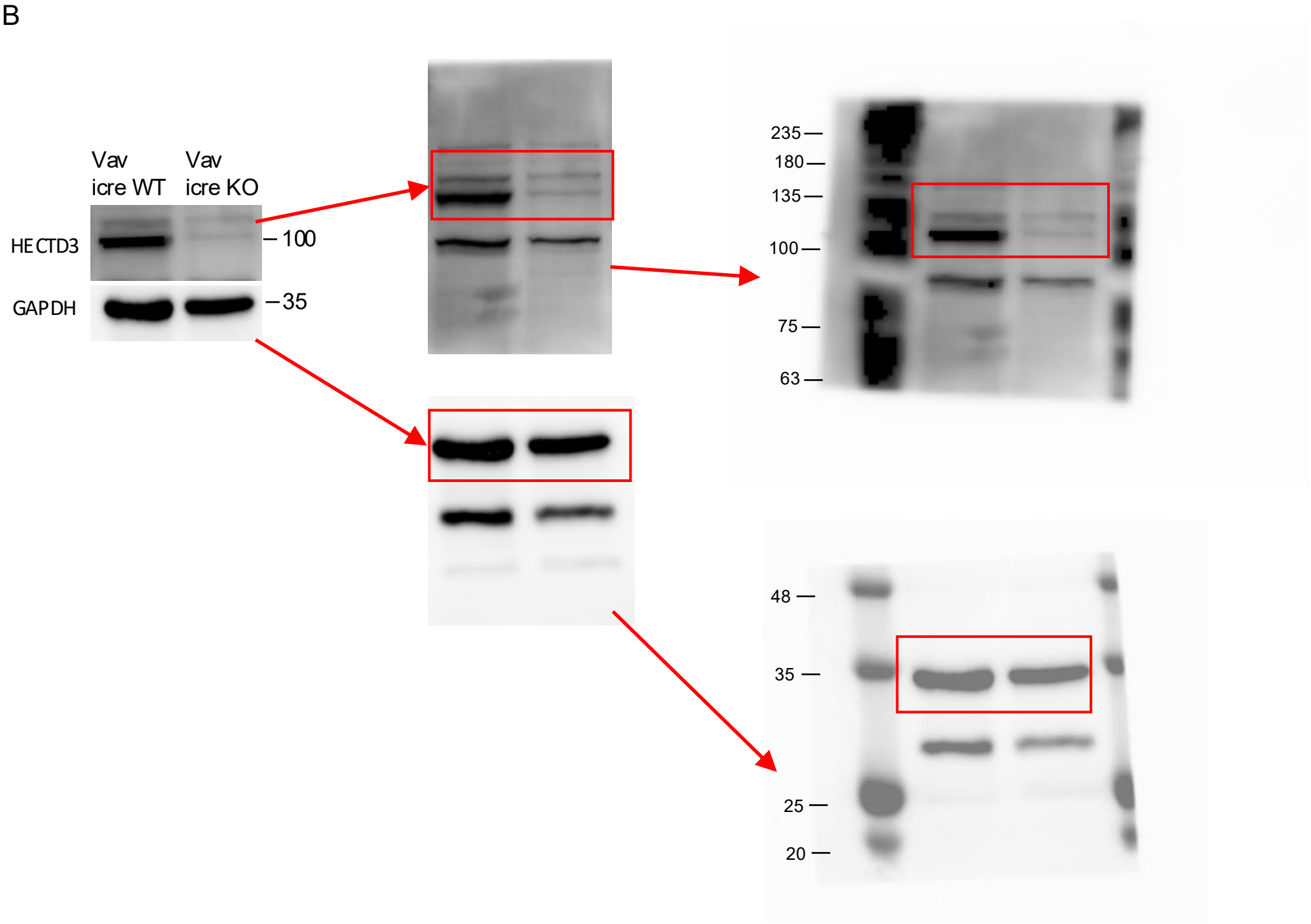

Original image with molecular weight marker

Full unedited gel

Figure S3

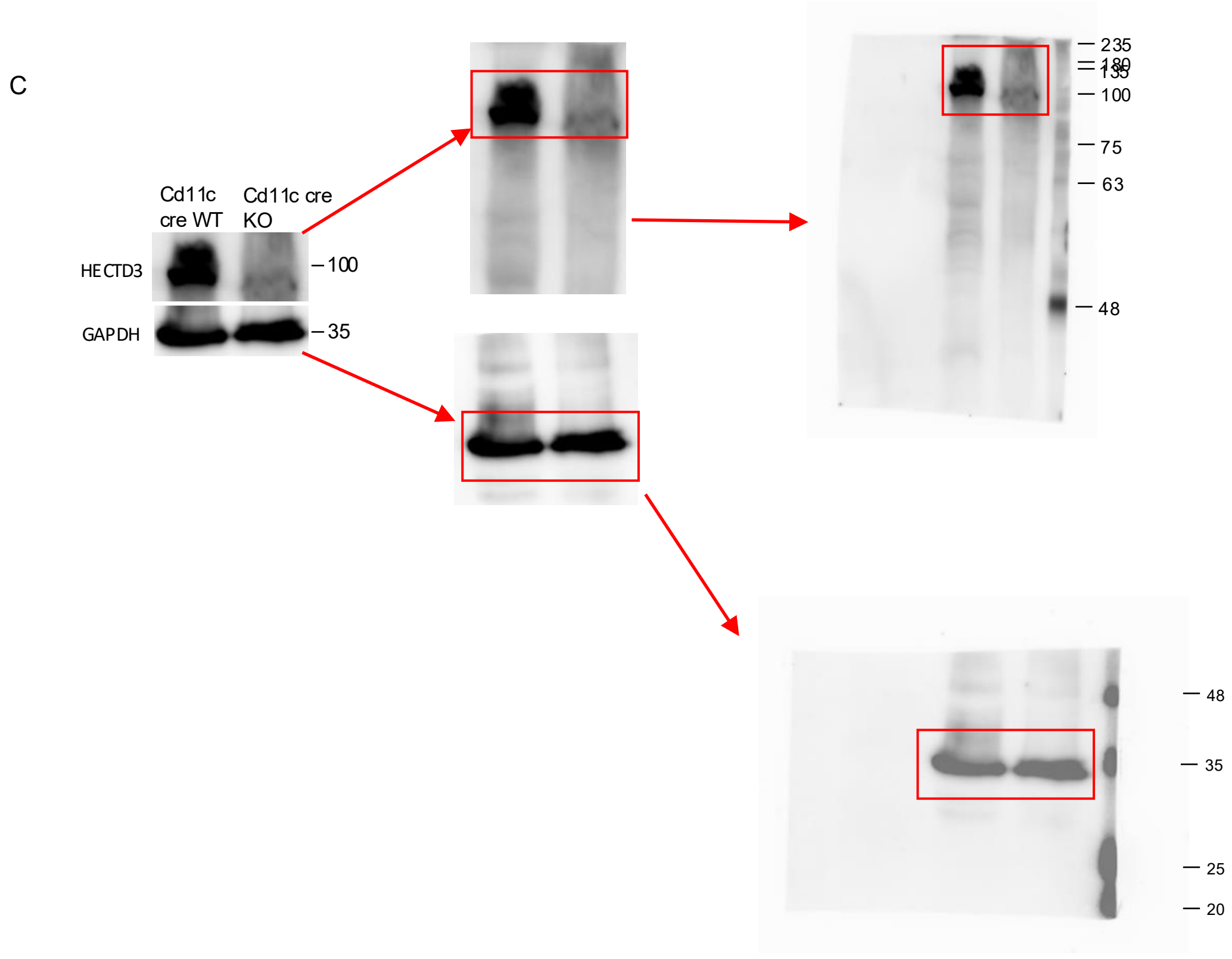

Original image with molecular weight marker

Full unedited gel

Figure S6

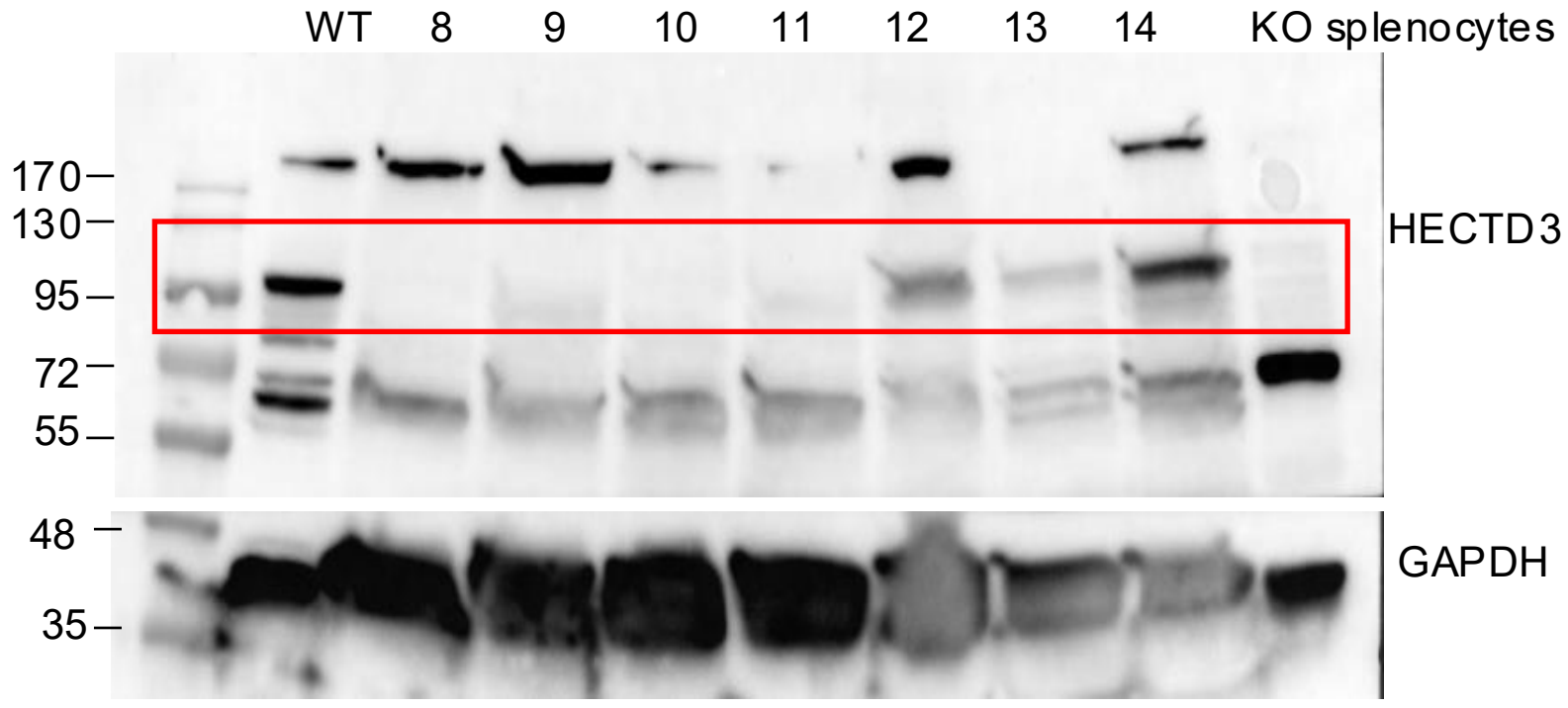

Original image with molecular weight marker

Full unedited gel

Figure S7

A

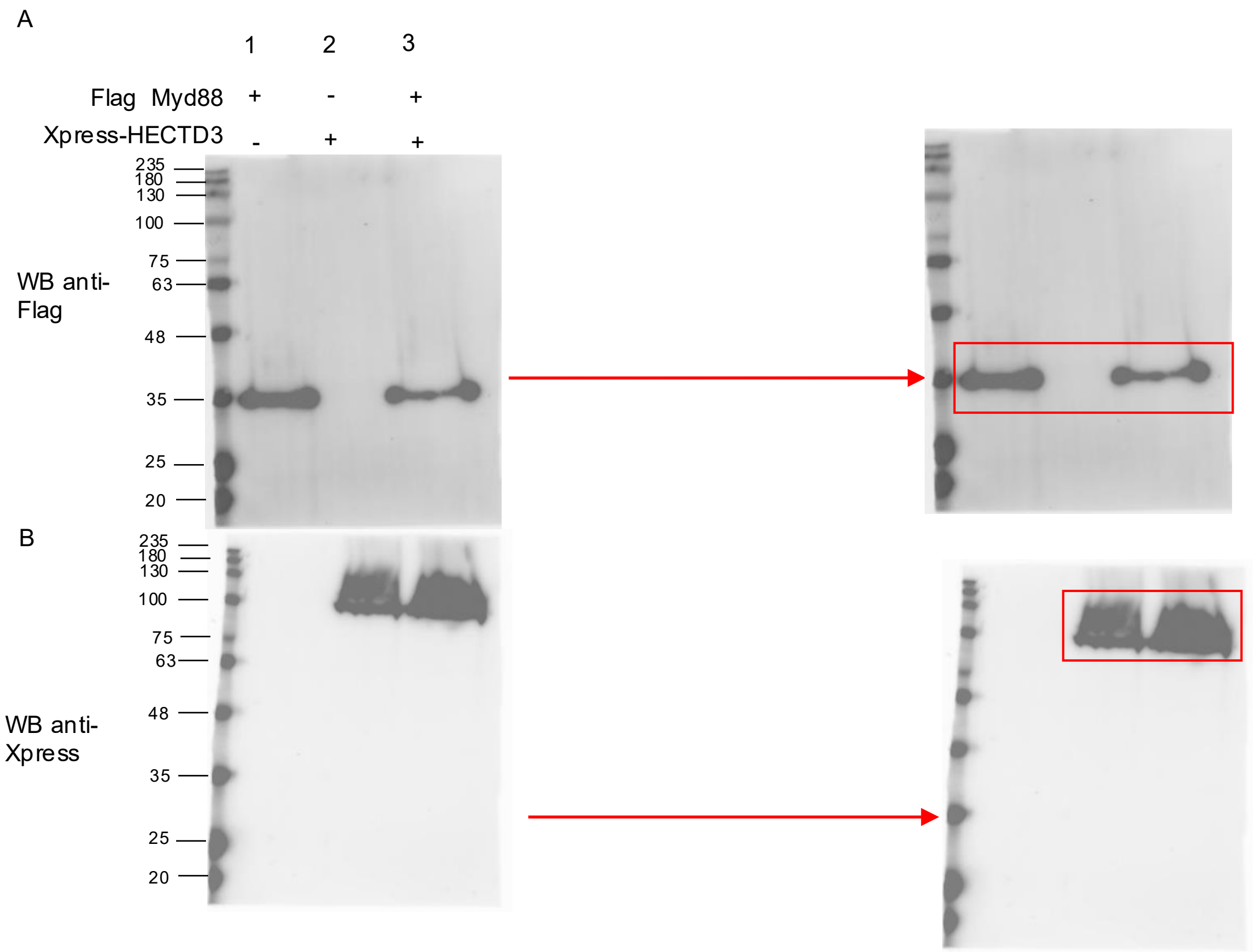

Original image with molecular weight marker

Full unedited gel

Figure S8

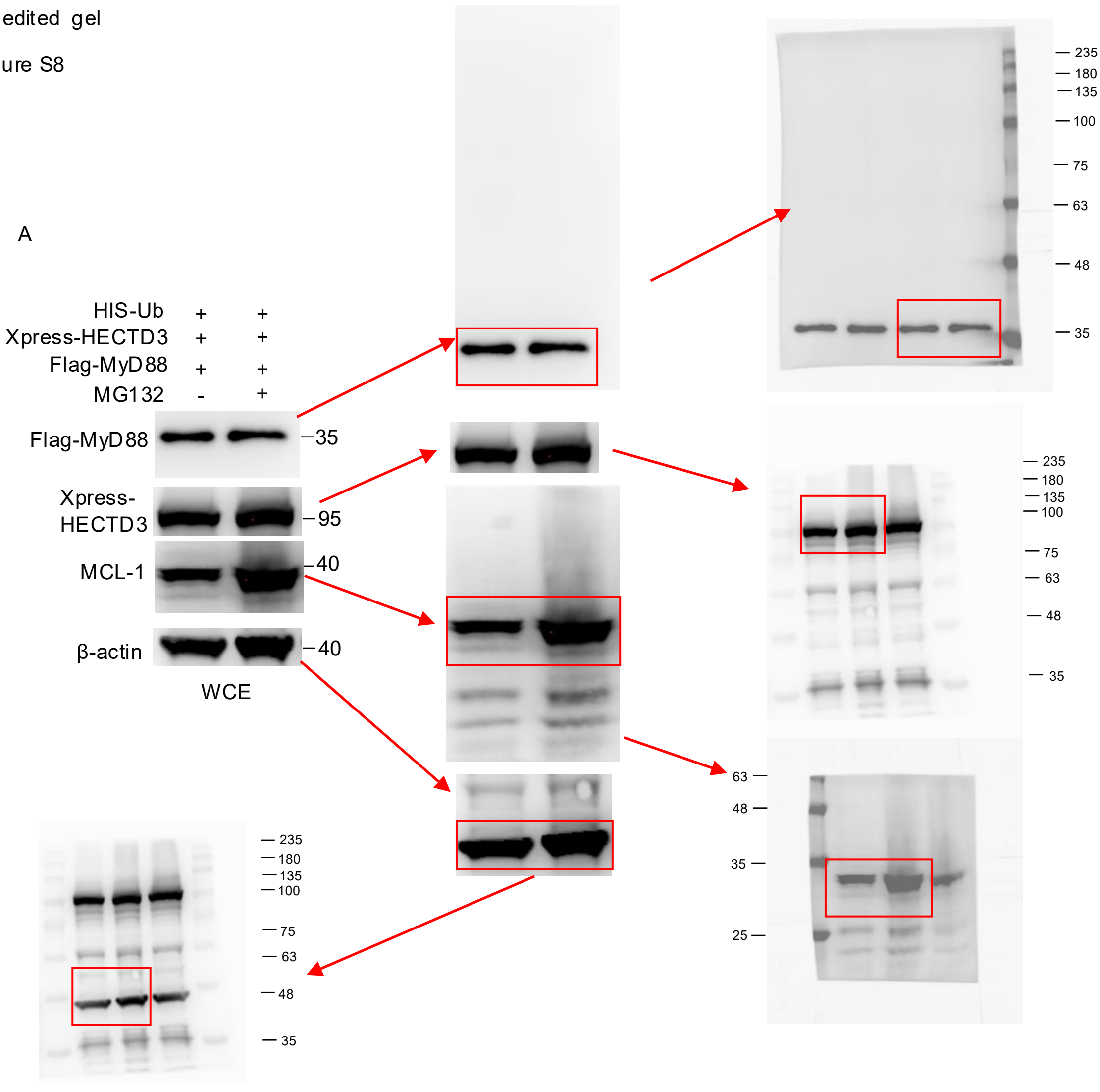

Original image with molecular weight marker

Full unedited gel

Figure S7

B

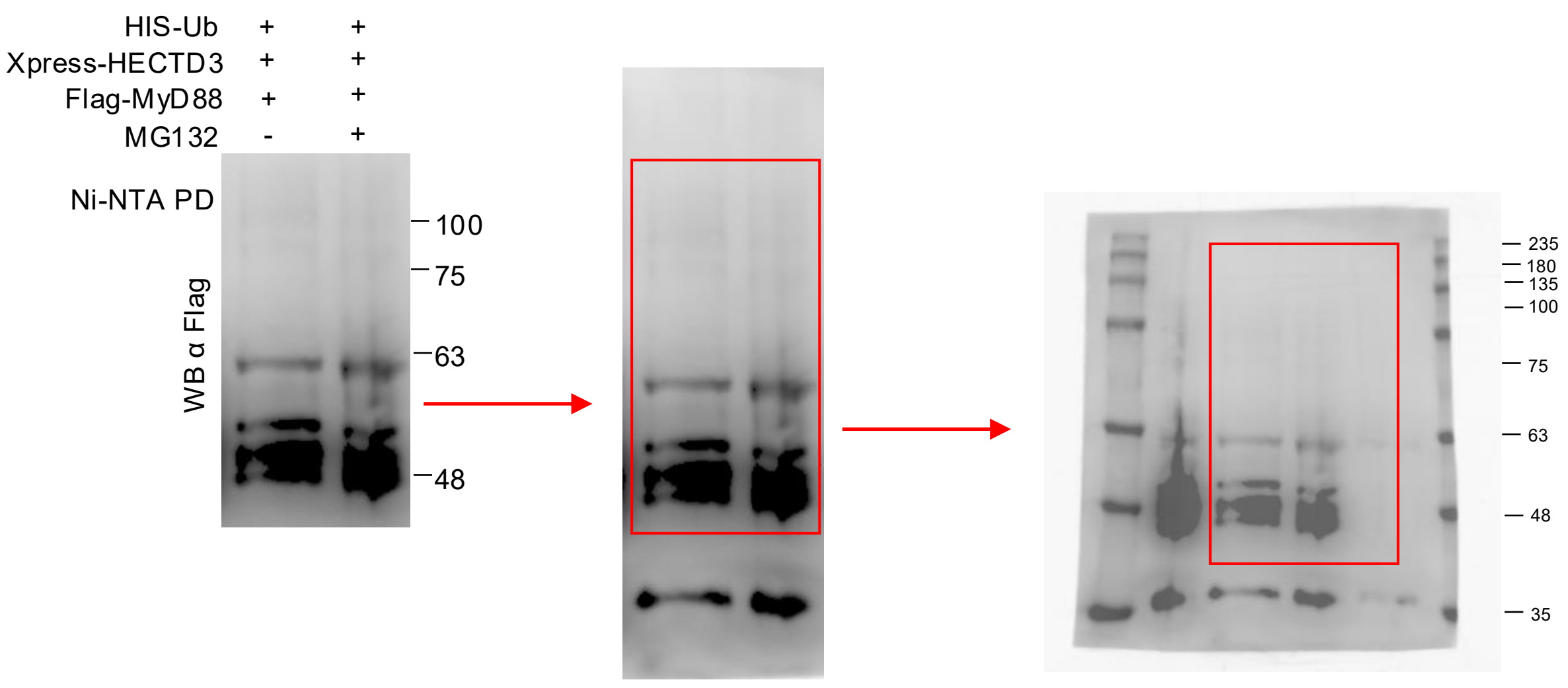

Supplement: Supplementary file 2 [file jciinsight-11-185437-s063.pdf]
